# Management of adult insomnia in Australia: a joint position statement of the Australasian Sleep Association, Sleep Health Foundation, Royal Australian College of General Practitioners, Australian Psychological Society, Pharmaceutical Society of Australia, and Australian Primary Health Care Nurses Association

**DOI:** 10.1093/sleepadvances/zpag086

**Published:** 2026-07-29

**Authors:** Alexander Sweetman, Stacey Putland, Hailey Meaklim, Sara Winter, Gerard Kennedy, Leon Lack, Delwyn Bartlett, James Szeto, Sarah Blunden, Henrique Salles, Vikki White, Amelia Scott, Cele Richardson, Nicole Grivell, Jenny Haycock, Daniel Sullivan, Erin Oldenhof, Shani Pickering, Moira Junge, Bianca Cannon, Linda De George-Walker, Yaqoot Fatima, Wayne Williams, Eron Cripps, Janet M Y Cheung, Camilla Hoyos, Darren Mansfield, Joel Aizenstros, David Cunnington, Melissa Ree

**Affiliations:** Australasian Sleep Association, Sydney Australia; School of Psychological Science, Perth, University of Western Australia, Western Australia, Australia; Bond University, Gold Coast, Queensland, Australia; Australasian Sleep Association, Sydney Australia; University of Melbourne, Melbourne, Victoria, Australia; Institute for Breathing and Sleep, Austin Health, Melbourne, Victoria, Australia; Flinders Health and Medical Research Institute, Sleep Health, Flinders University, Bedford Park, South Australia, Australia; Allied Health Research Collaborative, The Prince Charles Hospital, Queensland, Australia; Faculty of Medicine, The University of Queensland, Queensland, Australia; School of Applied Psychology, Griffith University, Queensland, Australia; School of Health and Biomedical Sciences, College of Science, Engineering & Health, RMIT University, Bundoora, Victoria, Australia; Flinders Health and Medical Research Institute, Sleep Health, Flinders University, Bedford Park, South Australia, Australia; Centre for Sleep and Chronobiology, Woolcock Institute of Medical Research, Macquarie University, Sydney, New South Wales, Australia; Reconnexion, Melbourne, Victoria, Australia; Appleton Institute of Behavioural Science, Central Queensland University, Australia; School of Educational Psychology and Counselling, Monash University, Melbourne, Australia; Reconnexion, Melbourne, Victoria, Australia; Level Up Psychology, Melbourne, Victoria, Australia; Centre for Sleep and Chronobiology, Woolcock Institute of Medical Research, Macquarie University, Sydney, New South Wales, Australia; School of Psychological Sciences, Macquarie University, Sydney, New South Wales, Australia; School of Psychological Science, Perth, University of Western Australia, Western Australia, Australia; Institute for Breathing and Sleep, Austin Health, Melbourne, Victoria, Australia; Flinders Health and Medical Research Institute, Sleep Health, Flinders University, Bedford Park, South Australia, Australia; Department of Physiotherapy, Melbourne School of Health Sciences, University of Melbourne, Heidelberg, Victoria, Australia; Flinders Health and Medical Research Institute, Sleep Health, Flinders University, Bedford Park, South Australia, Australia; School of Applied Psychology, Griffith University, Queensland, Australia; Thompson Institute, University of the Sunshine Coast, Queensland, Australia; Child Health Research Centre, University of Queensland, Queensland, Australia; Reconnexion, Melbourne, Victoria, Australia; School of Psychology, Deakin University, Melbourne, Victoria, Australia; Pharmaceutical Society of Australia, Australia; Sleep Health Foundation, Australia; Monash University, Melbourne, Victoria, Australia; Royal Australian College of General Practitioners, Australia; University of Sydney, Sydney, New South Wales, Australia; Australian Psychological Society, Australia; University of the Sunshine Coast, Queensland, Australia; Indigenous Health Education, Academic Lead Indigenous Health, The University of Queensland Rural Clinical School, Australia; Consumer representative, Australia; School of Pharmacy, Faculty of Medicine and Health, The University of Sydney, Sydney, New South Wales, Australia; Centre for Sleep and Chronobiology, Woolcock Institute of Medical Research, Macquarie University, Sydney, New South Wales, Australia; Epworth Sleep Centre, Melbourne, Victoria, Australia; Monash University, Melbourne, Victoria, Australia; Victoria Clinic, Melbourne, Victoria, Australia; Sunshine Coast Respiratory and Sleep, Queensland, Australia; Sleep Matters, Perth, Western Australia, Australia

**Keywords:** sleeplessness, difficulties initiating and maintaining sleep, sleep disorder, nonpharmacological, pharmacotherapy, CBTi, behavioral sleep medicine, primary care, models of care

## Abstract

This joint position statement of Australian sleep and primary care organizations provides information to healthcare providers on the management of adults with insomnia symptoms in Australia. Perspectives of sleep researchers, psychologists, sleep physicians, general practitioners, pharmacists, nurses, advocacy and implementation experts, and people with lived experience of insomnia are represented. Insomnia may present as an acute disorder (lasting up to 3 months) or a chronic disorder (lasting for 3 months or longer). Untreated insomnia is associated with lowered mood, daytime function, productivity, and quality of life. Insomnia assessment and diagnosis is based on self-reported symptoms. Insomnia occurring in the context of other conditions should be considered a “comorbid condition” that requires concurrent and targeted assessment and management. Recommendations about healthy sleep practices (sleep hygiene) are not an adequate stand-alone treatment for chronic insomnia. Multi-component Cognitive Behavioral Therapy for insomnia (CBTi) is the recommended “first-line” treatment for insomnia. There is evidence that CBTi is effective in a range of settings, in the presence of comorbid conditions, and when delivered in different modalities (e.g. individual sessions, group settings, tele-health, self-guided digital programs). Other evidence-based psychological and behavioral therapies may be appropriate for specific presentations, and in people that do not respond to CBTi. Medicines used for insomnia management are not recommended as the “first-line” treatment for insomnia but may be appropriate in conjunction with CBTi or as second-line treatment if CBTi is not effective or appropriate. Sleep medications may be considered in people with acute insomnia that is caused by a clear precipitant (e.g. bereavement, medical crisis, psychosocial stressors) and resulting in significant distress and/or functional impairment. If used, specific benzodiazepines and “z-drugs” may be indicated for up to 2–4 weeks of use at the lowest effective dose, melatonin may be used for up to 13 weeks, and dual orexin receptor antagonists are generally only recommended for chronic insomnia. Sedating antihistamines, antidepressants, antipsychotics, cannabinoids, and complementary medications are not recommended. Collaboration between people with insomnia and a range of clinicians is important for the management of insomnia and associated sleep, mental, and physical health conditions. Collaboration between sleep and primary care organizations has led to the development and implementation of healthcare provider education programs on insomnia assessment and management.

**Statement of Significance**

Insomnia disorder is highly prevalent in Australian adults, costing $19.1 billion AUD annually and increasing risks of mental and physical illness. Many people with insomnia are initially identified and managed in primary care settings. This joint position statement from six Australian sleep and primary care organizations therefore aims to provide updated, contextualized guidance on insomnia identification, assessment, treatment, and referral in the Australian healthcare system, with a particular focus on primary care. The statement integrates the perspectives of people with lived experience of insomnia, clinicians, researchers, and representatives of professional sleep and primary care organizations.

## Development and Aims of this Position Statement

Insomnia disorder is a prevalent sleep disorder in Australia that is associated with substantial individual, societal, and economic consequences. For example, insomnia is associated with reduced daytime function, mental health, physical health and quality of life, and costs Australia $19 billion annually in direct healthcare costs, reduced productivity, and quality of life [1–3]. Insomnia is among the most common conditions managed in primary care settings, and it is important to provide primary care clinicians and other healthcare professionals up to date information on the etiology, consequences, identification, diagnosis, and management of insomnia in Australia.

This Insomnia Position Statement was developed in 2024–2026 by representatives of the Australasian Sleep Association (ASA), Sleep Health Foundation (SHF), Royal Australian College of General Practitioners (RACGP), Australian Psychological Society (APS), Pharmaceutical Society of Australia (PSA), Australian Primary Health Care Nurses Association (APNA), and sleep researchers and clinicians in Australia. It aims to update and expand on a 2017 insomnia position statement developed by Ree and colleagues on behalf of the ASA [4]. Development of this Position Statement was supported by a 2022–2026 Australian Commonwealth Government Health Peak and Advisory Bodies grant and a 2023–2025 Quality Use of Diagnostics, Therapeutics and Pathology grant coordinated by the ASA. The Position Statement has been reviewed and approved by the ASA Clinical Committee, ASA Board, APS, PSA, APNA, and is officially recognized as an Accepted Clinical Resource by the RACGP. Further information on the development of the position statement can be found in the Supplement.

## Key Points and Recommendations

Key points and recommendations regarding the prevalence, consequences, assessment, diagnosis, treatment, and referral of adults with insomnia in Australia are displayed in Table 1.

**Table 1:** Key points

Prevalence and consequences	
1	Insomnia is the most prevalent clinical sleep disorder in Australia. Insomnia is more prevalent in females and older adults; however, it can occur in all genders throughout the lifespan.
2	Untreated insomnia is associated with significant individual, societal, and economic consequences including impaired daytime function, increased risks of mental and physical health conditions, reduced quality of life, and increased economic costs.
**Assessment and diagnosis**	
3	Diagnosis of acute (<3-month duration) and chronic (≥3-month duration) insomnia disorder is based on patient self-reported symptoms guided by; structured clinical interview, questionnaires, and a sleep diary. Primary care clinicians should consider using brief insomnia screening tools in patients with suspected insomnia.
4	Insomnia occurring in the presence of mental and physical health conditions should be viewed as a comorbid condition. This is because chronic insomnia is often responsive to targeted assessment and treatment in the presence of other chronic conditions.
5	Insomnia frequently co-occurs with other sleep–wake disorders. It is important to consider the presence of other underlying sleep disorders that could be contributing to insomnia symptoms, or operating as a comorbid disorder.
6	Referral for an overnight sleep study or review by a sleep physician may be considered if a comorbid sleep disorder is suspected, or if the patient does not respond to CBTi.
**Management and referral**	
7	Cognitive Behavioral Therapy for insomnia (CBTi) is the recommended “first-line” treatment for insomnia. CBTi can be delivered via a range of modalities including; individual or group delivery, in-person or tele-health, and via self-guided or clinician-guided digital CBTi programs. Clinicians should discuss CBTi options with all patients with insomnia as the first-line management approach.
8	Information about general healthy sleep habits (also called *sleep hygiene*) is not a sufficient stand-alone treatment for chronic insomnia.
9	Sleep medications (including specific benzodiazepines, “z-drugs,” melatonin, and dual orexin receptor antagonists) may be appropriate for short-term or intermittent management of *acute insomnia* in the following context; Insomnia symptoms are caused by an obvious underlying precipitating factor (e.g. acute grief/bereavement, injury), Insomnia symptoms are resulting in significant distress or functional impairment, and The person with insomnia is not appropriate for management with CBTi, and/or has engaged but not responded to CBTi. Some sleep medications may also be appropriate for *chronic insomnia* where in addition to the above, the patient is not appropriate for CBTi or has not responded to CBTi. In either case, careful consideration of the potential effectiveness of medications against possible side-effects, adverse events, age, informed patient preference, comorbid conditions, and patterns of dependence is encouraged. If used, benzodiazepines and “z-drugs” should be used for a maximum of 2–4 weeks at the lowest effective dose. Melatonin can be used for up to 13 weeks and there are different recommendations in Australian guidelines for duration of specific dual orexin receptor antagonists.

CBTi, Cognitive Behavioral Therapy for insomnia.

## Insomnia Disorder

### Definition

Diagnostic criteria for insomnia disorder are included in the International Classification of Sleep Disorders (Table 2) [5], Diagnostic and Statistical Manual of Mental Disorders [6], and International Classification of Diseases [7]. Insomnia disorder typically presents as frequent self-reported difficulty initiating sleep, maintaining sleep, and/or undesired early morning awakenings from sleep [5]. For a diagnosis of insomnia disorder, these sleep symptoms should be present despite adequate opportunity for sufficient sleep (e.g. sleep symptoms are not directly attributable to environmental factors causing awakenings, such as waking for overnight caring responsibilities). Common daytime symptoms include: fatigue, lethargy, mood disturbances, cognitive impairment, and diminished performance or engagement in work or social activities. Insomnia disorder can be defined as an acute disorder (lasting up to 3 months), or a chronic disorder (lasting 3 months or longer) [5]. Many individuals who present to healthcare providers with insomnia will have unsuccessfully tried many strategies to improve their sleep and may express frustration or anxiety about their persistent sleep difficulties. Unhelpful coping behaviors and/or preoccupation with sleep may develop in individuals experiencing insomnia for a long duration.

**Table 2:** Adapted international classification of sleep disorders (3rd Ed, text revision) insomnia disorder diagnostic criteria

A) The patient reports one or more of the following: Difficulty initiating sleep Difficulty maintaining sleep Final awakening earlier than desired B) The patient reports one or more of the following related to the nighttime sleep difficulty: Fatigue/malaise Attention, concentration, or memory impairment Impaired social, family, occupational, or academic performance Mood disturbance/irritability Daytime sleepiness Reduced motivation/energy/initiative Proneness for errors/accidents Concerns about or dissatisfaction with sleep C) The reported sleep/wake complaints cannot be explained purely by inadequate opportunity (i.e. enough time is allotted for sleep) or inadequate circumstances (i.e. the environment is safe, dark, quiet, and comfortable) for sleep D) The sleep disturbance and associated daytime symptoms occur at least three times per week E) (*Chronic insomnia disorder*) The sleep disturbance and associated daytime symptoms have been present for at least 3 months. (*Acute insomnia disorder*) The sleep disturbance and associated daytime symptoms have been present for less than 3 months. F) The sleep disturbance and associated daytime symptoms are not solely due to another current sleep disorder, medical disorder, mental disorder, or medication/substance use.

Regarding criterion F, it is often difficult to determine from baseline factors if insomnia is “solely due to” other sleep, medical, mental, or medication/substance use factors. Therefore, insomnia should be viewed as a “comorbid” condition in the presence of other mental, physical, and sleep conditions.

From International Classification of Sleep Disorders, third edition, text revision. Copyright American Academy of Sleep Medicine. Used with permission. This reproduction has been modified from the original to exclude pediatric specifications.

Insomnia is diagnosed according to self-reported symptoms [5, 6]. An overnight sleep study is not required for a diagnosis of insomnia. However, insomnia frequently co-occurs with other sleep disorders (e.g. obstructive sleep apnea, periodic limb movements of sleep. See Table S1). Referral to a sleep physician or direct referral by a general practitioner (GP) for an overnight sleep study may be appropriate for patients with both insomnia and a suspected comorbid sleep disorder.

Diagnostic schema previously sub-categorized insomnia as a “primary” or “secondary” condition based on the absence or presence of other mental/physical/sleep conditions. Insomnia frequently occurs in the presence of other chronic conditions. Recent schema [5, 6] recommend that insomnia occurring in the presence of other conditions should be viewed and managed as a *comorbid* condition. This is partly due to substantial evidence that co-occurring insomnia is often responsive to targeted assessment, diagnosis, and treatment [5, 6]. Insomnia should not be routinely viewed as a “secondary symptom” when occurring in the presence of other conditions. See Supplement for information on differential diagnoses and Table S1 for comorbid sleep disorders associated with insomnia.

#### Acute and chronic insomnia

Diagnostic schema distinguish between “acute” and “chronic” insomnia (Table 2) [5], and historically, this distinction may have been used to guide different management approaches (e.g. using pharmacotherapy for “acute insomnia” and CBTi for “chronic insomnia”). However, the “3-month” threshold is arbitrary in practice and does not reflect a binary change in the psycho-physiology of insomnia, the underlying causal mechanisms, or most appropriate management approach in each individual. In this position statement, recommendations about different interventions for insomnia are also based on the underlying causes of insomnia in each individual. For example, one individual may have experienced insomnia disorder for 2 months (i.e. “acute insomnia”) that is maintained by underlying psycho-behavioral factors for which CBTi is indicated [8], while another individual may have experienced insomnia disorder for 2 months (i.e. “acute insomnia”) that is directly maintained by bereavement, injury, or work/relationship precipitating stressors for which consideration of pharmacotherapy may be indicated [9]. Therefore, this position statement not only includes reference to both “acute” and “chronic” insomnia (e.g. when describing epidemiological evidence or recommendations of other guidelines) but also encourages assessment and consideration of precipitating factors and underlying perpetuating (maintaining) factors of insomnia when determining the appropriate management approach (see Etiology).

### Insomnia prevalence

Insomnia is the most prevalent sleep disorder in Australia. At any given time, approximately 30%–50% of adults experience acute insomnia symptoms (<3 month duration), and 10%–15% fulfill conservative diagnostic criteria for chronic insomnia disorder (≥3 month duration) [5, 10]. Insomnia can occur in all genders throughout the lifespan but is more prevalent in females, older adults, and those with comorbid sleep conditions, physical and/or psychiatric conditions [1, 11–14].

Insomnia is a frequent presentation in Australian primary care. The RACGP Health of the Nation survey has reported that psychological problems including sleep disturbance are among the most common reasons that patients present to general practitioners [15]. Sleep problems are reported in approximately 80%–90% of people with Major Depressive Disorder and 70%–80% of people with anxiety disorders [16], and are frequently managed in psychological practice [17, 18] and pharmacy settings [19].

### Consequences

Untreated insomnia is associated with substantial individual, societal, and economic consequences in Australia [3, 20]. Symptoms of daytime fatigue, lethargy, concentration difficulties, and memory problems are often cited as the reason that people with insomnia seek treatment, rather than the difficulties initiating or maintaining sleep per se [21]. Over time, the combination of sleep difficulties; increased concern and anxiety about sleep; and reduced energy, mood, and motivation can culminate in reduced quality of life [22]. Insomnia is one of the most consistent predictors for the development of mental health conditions [23].

Epidemiological studies have reported associations between untreated insomnia and incident symptoms and/or diagnoses of mental health problems including depression, anxiety, alcohol abuse, psychosis, and risks of self-harm and suicidal ideation [23–25]. The relationship between sleep and mental health is likely bi-directional [17]. Insomnia is also associated with a higher prevalence of pain [26], hypertension [27], and risks of workplace and motor-vehicle accidents [28, 29]. In 2021, insomnia resulted in economic costs of $19.1 billion AUD annually through reduced work productivity, increased use of health resources, and reduced quality of life [1–3]. Insomnia is not associated with an increased risk of all-cause mortality [30, 31]. However, increased risk of mortality has been reported in association with the management of insomnia with hypnotics [32, 33], daytime impairments associated with insomnia [34], and insomnia comorbid with untreated obstructive sleep apnea [35–37].

### Etiology

**Figure 1:**
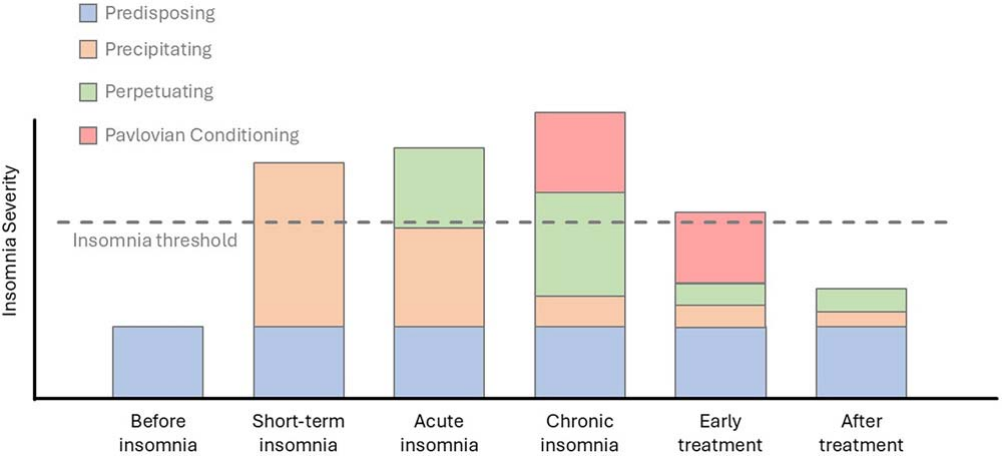
4P model of insomnia aetiology and impact of cognitive behavioral therapy for insomnia. Adapted from Perlis and colleagues [39]. Chronic insomnia is proposed to result from a combination of pre-disposing (risk) factors, precipitating (trigger) factors, perpetuating (maintaining) factors, and a state of Pavlovian (conditioned) insomnia.

Chronic insomnia is proposed to result from a combination of predisposing, precipitating, perpetuating, and Pavlovian operant conditioning factors (Figure 1) [38, 39]:

- **Predisposing factors** include any bio-psycho-social traits that increase a person’s risk of experiencing sleep disturbance (e.g. trait anxiety, female sex, a family history of insomnia). Predisposing factors are relatively stable over time and, in isolation, are not sufficient to cause or maintain insomnia disorder.
- **Precipitating factors** represent the initial cause of short-term sleep disturbance. These can include acute mental stressors (e.g. bereavement, work/study stress), physical stressors (e.g. injury, pain), physiological factors (e.g. hormonal changes, the side effect of a new medication), or factors that cause internal body clock desynchrony (e.g. jet lag, shift work). Precipitating factors may result in short-term insomnia symptoms (e.g. for 1–2 weeks).
- **Perpetuating factors** include any psychological and behavioral factors that maintain the insomnia over time and allow the insomnia to develop independence from the initial precipitating factors. Often, people with insomnia may engage in specific behaviors with the intent to improve their sleep or compensate for lost sleep, but these can exacerbate the overall insomnia condition. For example, people with insomnia often extend the amount of time that they spend in bed in an attempt to obtain as much sleep as possible. However, this more often results in increased time spent *awake* in bed, which can reinforce insomnia. Alternatively, reducing activity after a night of poor sleep (e.g. canceling daytime activities, extended naps) can help people function during the day but interferes with their subsequent sleep quality, thereby perpetuating the insomnia. Different forms of “sleep effort” (e.g. rituals, thoughts and behaviors that are used with the intention of falling asleep) can become counter-productive and delay sleep onset or prolong awakenings [40].
- **Pavlovian and operant conditioning factors** refer to a conditioned (learned) association between the bed, bedroom or pre-sleep routine, and a state of mental and physical alertness. People with insomnia may spend extended time in bed experiencing feelings of worry, frustration, alertness, and tension. Over repeated episodes of being awake and alert in bed, a conditioned association can form between the bed/bedroom and a state of alertness or arousal. The bedroom environment or the act of getting into bed can consequently become a conditioned stimulus that elicits a state of alertness, worry, anxiety, and frustration (i.e. conditioned insomnia), rather than a state of rest and relaxation that is compatible with sleep.

#### Course of insomnia

Approximately 30%–50% of adults report acute insomnia symptoms (<3-month duration) at any given time [10, 41]. Longitudinal studies indicate that acute insomnia remits in approximately 70%–80% of people, with only 2%–20% experiencing progression from acute to chronic insomnia (≥3-month duration) [41, 42]. A large number of people who do not progress to fulfil criteria for chronic insomnia disorder may experience intermittent periods of acute insomnia, remission, persistent poor sleep, or relapse of acute insomnia symptoms over time [42]. Although this pattern of persistent poor sleep may not fulfil conservative diagnostic criteria for “chronic insomnia disorder” (i.e. unbroken period of frequent sleep difficulties for at least 3 months), management of sleep difficulties should be considered based on the recurring course of their sleep problems.

After chronic insomnia disorder develops, it often persists over time. For example, Morin and colleagues [43] investigated the incidence, remission, and persistence of insomnia symptoms in a sample of 3073 adults over a 5-year period. Although only 14% of those defined as “good sleepers” at baseline developed insomnia disorder across the 5-year follow-up, 37.5% of those with insomnia at baseline reported persistent symptoms at each of the five annual follow-up intervals. Other population-based studies have reported higher insomnia persistence rates of 44–74% at 1-year follow-up [44–47].

### Comorbid insomnia

Insomnia frequently co-occurs with other mental, physical, and sleep conditions. Although insomnia has historically been viewed as a “secondary symptom” when occurring with other chronic conditions [48], there is now overwhelming evidence that insomnia can rapidly develop independence of other conditions and that bi-directional relationships between sleep difficulties and other health problems exist [48]. Psychological and behavioral treatment of insomnia often improves insomnia symptoms in the presence of co-morbid conditions [49–51], and may improve symptoms and/or “downstream” management of the comorbidity (e.g. depression, anxiety, pain) [52]. Therefore, insomnia should be viewed as a “comorbid” condition in the presence of other mental, and physical health symptoms/conditions, to promote targeted assessment and management of the insomnia alongside the management of comorbid conditions. See Supplement for prevalence and management recommendations for comorbid insomnia with different comorbid conditions.

## The Current Landscape of Insomnia Management in Australia

Medications, medical devices, and other treatments for insomnia in Australia are regulated by the Therapeutic Goods Administration. Access to medical and hospital services for eligible residents is supported by Medicare; Australia’s publicly funded universal health system. The Pharmaceutical Benefits Scheme (PBS) and Medicare Benefits Schedule (MBS) operate alongside Medicare, and respectively subsidize the cost of specific listed medications, and specific medical services for eligible patients (e.g. consultations, diagnostics, procedures, and treatments). Diagnostics and treatments for insomnia may also be accessed privately (without subsidization through these services), including private payment for consultations/medications/therapy, over-the-counter medications, and medications or care that are not listed on the PBS and MBS. Together, the regulatory body and these mechanisms for subsidized access to diagnostics and treatments influence the ways that insomnia is managed in Australia (see the Supplement for a description of these systems).

Australian evidence-based guidelines from the ASA [4, 53], RACGP [54], APS [55], and PSA [56] recommend Cognitive Behavioral Therapy for insomnia (CBTi) as the “first-line” treatment for chronic insomnia. However, it is estimated that only 1% of Australian adults with insomnia access CBTi [33, 57]. There are multiple system-level barriers that limit CBTi access and uptake in Australia that are presently being addressed in different ways [58] (see section on CBTi Access and Modalities). These barriers have been described in Australian quantitative survey studies [57], qualitative interview studies from the perspectives of people with insomnia [59–61], general practitioners [62], psychologists [63], nurses [64] and pharmacists [65], and an Australian CBTi implementation report [58].

People with insomnia often attempt self-guided remedies/approaches to improve sleep before presenting for management in primary care [66], and most people with insomnia do not access appropriate evidence-based care. There is a large amount of information, remedies, devices, and over-the-counter medications promoted for insomnia management in Australia. Despite limited or no evidence of effectiveness, some remedies may have sophisticated marketing campaigns that may be very convincing to people that are desperate for resolution of insomnia symptoms (See *Complementary and Alternative Medicines* section, Supplement). People that experience persistent insomnia symptoms despite attempting a large number of non-evidence-based approaches may gradually develop a state of “learned helplessness” [57]. This may also perpetuate insomnia and reinforce patterns of medication use for sleep. People with insomnia may be unaware that behavioral treatments are recommended for insomnia. Furthermore, some people may not discuss insomnia with their primary care clinician, if they believe that they have exhausted all possible treatment options other than prescription medications [67]. Asking patients about sleep is important, and opportunistic screening for insomnia in primary care can be useful. Increased public awareness of the importance of sleep and psychological treatments of insomnia is needed to ensure that more people know that there are evidence-based behavioral treatments available and that there is reason to discuss sleep concerns with their general practitioner or other clinicians.

The Australian Department of Health, Disability and Ageing have confirmed that insomnia is an eligible condition for psychological treatment under the Better Access initiative [68] (See below section on CBTi Access and Modalities). This allows general practitioners to prepare a Mental Health Treatment Plan and refer patients with insomnia to a psychologist or general practitioner with Level 2 Focussed Psychological Strategies training for psychological and behavioral management of insomnia. With a Mental Health Treatment Plan, patients can access a rebate for up to 10 in-person or tele-health consultations each calendar year. As many people with insomnia are unaware of behavioral treatments for insomnia, they may not understand why a Mental Health Treatment Plan and referral to a psychologist is needed, creating a barrier to treatment. The overall cost of psychologist/general practitioner consultations (set by providers) is often higher than the Medicare rebate received by patients, and the cost of psychological treatment can be a barrier to treatment access [57, 63]. Although 10 psychological sessions are sufficient for delivery of most CBTi programs, people with multiple mental health conditions may quickly use the 10 available annual sessions that include a Medicare rebate and consequently would be required to privately fund any additional sessions (e.g. patients with insomnia comorbid with depression, anxiety, post-traumatic stress disorder). Hence, additional barriers to CBTi access may be experienced by people with comorbid mental health conditions.

Digital CBTi programs have been found to improve symptoms of insomnia and associated mental health symptoms [69–71]. Several static and interactive digital CBTi programs with scientific evidence are becoming increasingly available in Australia [72–78]. Digital mental health programs (including programs that deliver CBTi) that conform to accepted clinical practice guidelines and present relevant evidence-based guidelines to users are presently exempt from Therapeutic Goods Administration (TGA) regulatory approval (as of January 2025). For appropriate patients, digital CBTi potentially represents a resource-efficient, effective, and rapidly accessible option to improve CBTi access and relieve pressure on the health system, when integrated alongside existing healthcare professionals and systems.

## Overall Management Considerations

Treatment for insomnia aims to improve the presenting self-reported sleep difficulties and consequences of poor sleep on daytime feeling, functional, social, and/or occupational factors of each individual patient. A person-centered approach is recommended to include informed patient choices and treatment preferences in decisions for pharmacological and non-pharmacological management approaches. Insomnia disorder presentation is heterogeneous, and a person-centered approach is recommended. Randomized controlled trials of pharmacological and non-pharmacological interventions for insomnia often assess self-reported and objectively measured outcomes including global insomnia severity, sleep (e.g. sleep diary or objective sleep metrics), daytime function and feelings (e.g. fatigue, concentration, sleepiness), mental health symptoms (e.g. depression, anxiety, stress), quality of life, and sleep medication use [79, 80].

Although CBTi is the recommended “first-line” treatment for chronic insomnia, it does not result in immediate improvements in sleep and may not be appropriate for all patients with insomnia. For example, short-term management with sleep medications may be appropriate for patients who have used but not responded to CBTi previously, and patients with short-term insomnia resulting from an obvious precipitating factor that is causing significant distress or functional impairment (i.e. people that require more immediate relief) [9]. CBTi may not be appropriate in patients experiencing insomnia as a direct result of external stimuli that prevents sleep (e.g. environmental factors such as noise, temperature, caring responsibilities, or light), or obvious precipitating factors (e.g. bereavement, pain, injury). Furthermore, standard delivery of some CBTi components such as bedtime restriction therapy may not be appropriate in patients with comorbid bipolar, schizophrenia, or epilepsy, as acutely reduced sleep duration may exacerbate risks associated with these comorbid disorders. Additionally, nuanced management considerations may be appropriate in the presence of other specific comorbid conditions, work/lifestyle factors, and other concurrent treatments.

A comprehensive assessment of insomnia symptoms, history, previous, and current treatment/s, suspected and confirmed comorbid conditions, and person-centered goals of treatment should be conducted to guide the most appropriate management approach (see Table 3).

**Table 3:** Sleep history and symptoms that may be discussed to inform insomnia diagnosis

Type of factor	Specific discussion points/questions
Reason for seeking care	Reasons for seeking treatment to establish person-centred goals. Assess motivation and readiness/stage of change. In particular, CBTi is an effective treatment for insomnia, but requires sustained effort from patients and may temporarily reduce sleep duration and/or increase sleepiness before insomnia symptoms improve.
Sleep and functional symptoms of insomnia	Difficulties falling asleep. Difficulties maintaining sleep (frequent or long awakenings, timing, and any known causes of awakenings). Early morning awakenings. See Table 2 for examples of daytime symptoms associated with insomnia disorder.
Sleep pattern/s and environment	Sleep timing, regularity, consistency, before workdays vs non-workdays. Normal sleep duration. Sleep quality (including average time to fall asleep, number, timing, and duration of awakenings, and total wake time). Discuss strategies that the patient uses when they can’t sleep. General sleep difficulties (e.g. non-restorative sleep, poor sleep quality, fragmented sleep, etc.). Discuss sleep environment (bedroom temperature, light, noise, etc.). Comorbid sleep disorder symptoms, diagnoses, and treatments (see Table S1).
History of sleep problem and previous treatment/s	Family history of insomnia. Duration, onset, and course of insomnia symptoms, including any obvious factors that initially triggered insomnia, and patterns of insomnia remission and relapse over time. Any other current treatment/s for sleep problems, including sleep medications, and their patterns of use. Previous treatment/s that have been used for insomnia, other sleep disorder/s, and short-term and long-term impact of these treatment/s. Things that make insomnia symptoms better or worse. Any strongly held or anxiety-inducing beliefs about sleep, the immediate consequences of sleep loss, beliefs about “normal” or “healthy” sleep patterns, and long-term impacts of insomnia. Psycho-behavioral perpetuating factors of insomnia (see Aetiology). Patterns of “sleep effort,” including thoughts or behaviors carried out with the intention of inducing sleep (e.g. “trying hard” to fall asleep, which can be incompatible with rest/sleep). Assess for a state of “conditioned” insomnia, whereby the bed or bedroom environment has become an automatic trigger for a state of alertness, worry, anxiety, or mental/physiological arousal. Ask about sleep difficulties when sleeping in a different environment, to identify patterns of insomnia associated with the home sleep environment through a conditioned association. Ask about a sensation of “alertness” or an “overactive mind” triggered by the bedtime environment or routine.
Health and lifestyle factors	Work, life and contextual factors (e.g. work/study patterns and timing, shift work, family, or social factors related to sleep). Comorbid mental and physical disorders (including symptoms, risk factors, and treatment/s). Consider a self-harm risk assessment and management plan. Consider potential risks associated with bedtime restriction therapy in specific samples (e.g. comorbid bipolar, schizophrenia, epilepsy, occupation as commercial driver/pilot for work, work in emergency care, or shift work settings).

## Assessment

Diagnosis of insomnia is based on detailed clinical evaluation of patient self-reported history/symptoms (Table 3) and may be guided by questionnaires and sleep diary information (see Table 4). Understanding sleep history is important in the assessment of insomnia, and should include questions about insomnia subtypes, onset, previous treatments, other sleep-related symptoms, lifestyle factors, and daytime consequences (Table 4). It may be beneficial to map different features of a person’s insomnia symptoms onto the “4P model” to establish a person-centered model of insomnia development and maintenance. Several other sleep disorders can also present with insomnia symptoms, or occur comorbid with insomnia, and may require an overnight sleep study (see Table 4). Referral for a sleep study or consultation with a sleep physician may be considered in those with a suspected comorbid sleep disorder, excessive daytime sleepiness (high likelihood of unintentionally falling asleep during the day), general sleep difficulties that are non-specific to insomnia (e.g. chronically non-restorative sleep), and people who have used and not responded to CBTi.

**Table 4:** Insomnia and sleep assessment options

Modality	Description	Recommendation
Questionnaires	Questionnaires are useful in assessing severity of insomnia and associated daytime function/mental health symptoms before and after treatment. Brief insomnia screening questionnaires; Insomnia Severity Index [135], a 7-item measure of global insomnia severity. Sleep Condition Indicator [136], an 8-item measure of insomnia that maps to DSM-5 criteria. Athens Insomnia Scale [137], an 8-item measure of insomnia that maps to ICD-10 criteria. Daytime function questionnaires; Flinders Fatigue Scale [138] Epworth Sleepiness Scale [139] Top-End Sleepiness Scale [140] Daytime Insomnia Symptom Scale [141] The Dysfunctional Beliefs and Attitudes about Sleep scale [142] may be used to assess for potentially unhelpful beliefs about sleep that can perpetuate insomnia, and represent a target for cognitive therapy approaches. Assessing for “sleep effort” is also important, to identify any cognitive/behavioral patterns/rituals that are enacted with the intent of promoting sleep (but which can be counter-productive) [40]. It is also helpful to assess for mental health symptoms during the assessment of insomnia. There are many standardized mental health questionnaires that may be used [143–145].	Brief insomnia screening questionnaires are recommended for assessment of patients with suspected insomnia.
Sleep–wake diary	Digital or paper-based tools that allow users to self-report sleep and wake information from the previous night upon awakening and getting out of bed each morning for 1–2 weeks (at baseline), and as needed throughout treatment to guide CBTi recommendations.	Indicated for insomnia assessment.
Structured interview	A structured diagnostic interview for assessment and diagnosis of insomnia.	Gold standard for insomnia diagnosis.
Consumer sleep trackers	Consumer health trackers (including “wearable” and “nearable” sleep monitoring devices) have become increasingly available and used to monitor and track sleep patterns [146]. Examples include watches, rings, arm-bands, nearable devices, and mobile phone applications. The number and type/s of sensors used in sleep trackers differ considerably [146]. Consequently, the accuracy of algorithms used to infer sleep information also varies. The accuracy of information also differs considerably based on the parameters of interest (e.g. time in bed, sleep duration, sleep onset latency, sleep staging information, heart rate data, respiratory data). Sleep stage information (i.e. time spent in “light” versus “deep” sleep) is rarely validated. In people with insomnia, sleep trackers may contribute to increased anxiety about sleep (i.e. Orthosomnia [147]).	Not indicated for routine use in insomnia assessment. May be used in combination with self-report measures to guide assessment and management (e.g. identification of co-morbid sleep disorder symptoms).
Overnight sleep study	Sleep studies may be categorized into different “levels” according to the location of the study, and the specific information that is collected; Level 1: In-lab polysomnography sleep study. Level 2: Home-based polysomnography sleep study. Level 3: Portable limited 3–6 channel sleep study. Level 4: Portable limited 1–2 channel sleep study. For a description of overnight sleep studies, see Sleep Central (https://sleepcentral.org.au/Central/Contents/OSA/OSA-Sleep-studies.aspx).	Indicated in patients with insomnia and a suspected co-morbid sleep disorder.

## Psychological and Behavioral Management

There are several psychological and behavioral management approaches for insomnia that are supported by empirical evidence. CBTi has received substantial research attention and is consistently recommended as the “first-line” treatment for insomnia in evidence-based guidelines [4, 53, 79, 81–84]. Other psychological and behavioral approaches to manage insomnia symptoms have been studied, including healthy sleep habits, mindfulness-based therapies, intensive sleep retraining, and Acceptance and Commitment Therapy. See Supplement for a review of evidence and recommendations regarding these other psychological and behavioral insomnia management approaches. Several of these alternative psychological and behavioral therapies for insomnia may be considered for specific presentations or in patients who do not show adequate response to CBTi alone.

### Cognitive behavioral therapy for insomnia

Evidence-based insomnia management guidelines developed by sleep, primary care, and medical societies worldwide strongly recommend CBTi as the “first-line” treatment for insomnia [4, 53, 79, 81–84]. CBTi is a multi-component therapy that aims to identify and target the precipitating and underlying perpetuating factors of insomnia to improve sleep and daytime function (Table 5). Because CBTi treats the psychological and behavioral factors believed to maintain insomnia and identify/reduce the impacts of future precipitating factors, it is associated with moderate-to-large sustained improvements in sleep, daytime function, quality of life, and mental health [85, 86]. Hundreds of randomized controlled trials and over 40 meta-analyses worldwide [79, 87] have found that CBTi is an effective, durable, and safe treatment for insomnia, including when insomnia occurs in the presence of mental health symptoms/disorders [52, 88], pain [50], cardiovascular disease [89], sleep disorders [51], and in patients using sleep medications [90, 91]. Moderate-to-large and sustained improvements have been observed in symptoms of insomnia (global questionnaire measures), sleep parameters, mental health, daytime function, and reduced sleeping pill use following CBTi [79, 85, 87]. See Supplement for evidence investigating the effectiveness of CBTi for acute insomnia; comorbid insomnia; concurrent sleep medication use; risks/side-effects associated with CBTi; and stepped-care models for insomnia management in Australia.

**Table 5:** Components of Cognitive Behavioral Therapy for insomnia (CBTi)

Component	Type of component	Description
Psychoeducation about sleep	Educational	Information about the factors that control the timing and quality of sleep (sleep pressure, body clock), normal sleep patterns, and normal changes in sleep with age. Aims to provide a rationale and encourage engagement with behavioral therapy components. Psychoeducation and discussion about thoughts/behaviors representing “sleep effort” that may contribute to insomnia maintenance.
Healthy sleep habits [148] (sleep hygiene)	Educational	Information about behavioral and environmental factors that can “help” or “harm” our sleep (e.g. recommendations about screen use in bed; wind-down routine; timing of diet; exercise; caffeine and alcohol; and bedroom noise, light, and temperature). Not sufficient as a stand-alone intervention but often included in multi-component CBTi programs.
Stimulus control therapy [149]	Behavioral	Set of instructions that aims to treat the conditioned association between bed and a state of alertness/anxiety, and improve the automatic relationship between bed and sleep.
Bedtime restriction therapy [150] (Also called: *sleep restriction therapy, and sleep efficiency training*)	Behavioral	Bedtime restriction therapy aims to increase homeostatic sleep pressure by limiting time in bed to match sleep ability. After a patient begins sleeping for the majority of time spent in bed, prescribed time in bed is gradually up-titrated from week-to-week until an equilibrium between time in bed, sleep duration, and daytime sleepiness/alertness is achieved. Standard bedtime restriction therapy protocols may not be appropriate in patients with bipolar, schizophrenia, or unmanaged epilepsy (as reduced sleep duration may exacerbate risks associated with these other conditions).
Relaxation therapies [151, 152]	Behavioral	Numerous evidence-based relaxation therapies exist that aim to reduce stress/anxiety, and increase state of relaxation and rest. It may be more appropriate to practice relaxation therapies during the day or before getting into bed, rather than while in bed (to avoid relaxation therapies becoming a form of “sleep effort” [153]).
Cognitive therapies [154]	Cognitive	Tailored suite of therapies that aim to identify, test, and replace potentially unhelpful beliefs or attitudes about sleep that may cause anxiety/worry associated with sleep, and/or maladaptive behaviors that perpetuate patterns of insomnia.
Cognitive refocusing	Cognitive	Suite of evidence-based strategies to re-focus attention on neutral or positive non-sleep stimuli, to reduce anxiety or worry about sleep.
Adjunct therapies	Adjunct	Other components may be included in CBTi programs, including: Paradoxical intention therapy. Light therapy (for management of comorbid circadian rhythm disruption). Mindfulness-based therapies. Imagery rehearsal therapy. Behavioral activation. Acceptance and Commitment Therapy.

#### CBTi access and modalities

Although CBTi is the recommended “first-line” treatment for insomnia, it is presently accessed by only approximately 1% of adults with insomnia in Australia [33, 57]. To improve access to CBTi, a range of implementation strategies have commenced in Australia and worldwide [58, 92–96].

CBTi has traditionally been delivered by psychologists with training and expertise in the management of sleep disorders. Although this is considered the “gold-standard” modality to access CBTi, there is a shortage of psychologists with expertise in sleep medicine in Australia [58, 97]. Currently, seeking treatment from psychologists with experience in insomnia management often results in long wait lists and/or high costs to patients (including costs exceeding MBS Mental Health Treatment Plan rebates for sessions, see above). Therefore, accessing CBTi from a psychologist with training and expertise in sleep disorders management may not be feasible for many people with insomnia. Although CBTi can be provided via telehealth (with support by the Better Access initiative), limited access issues are still believed to be compounded in rural and remote locations of Australia [98].

Training programs have been developed to up-skill more Australian clinicians in CBTi delivery. In 2021, the ASA “psychologist education subcommittee” conducted an audit which identified only 65 psychologists in Australia with training and expertise in the management of sleep disorders (with very few in rural/remote locations) [58]. The ASA and APS have been collaborating on a range of CBTi education articles, webinars, events, and development of an interactive 6-hour CBTi education module. This has substantially increased the number of psychologists with CBTi training [93]. As of 2024, an online CBTi provider directory [99] (https://sleepcentral.org.au/Central/Contents/Clinicians-CBTi.aspx) can be used by patients and clinicians to identify local clinicians with CBTi training and expertise. This registry now includes clinicians in Australia and New Zealand with training and expertise in CBTi delivery, and/or those that have completed a 6-hour interactive CBTi education program developed by the ASA and APS. As of August 2025, >180 clinicians are listed on the registry.

Primary care clinicians can also deliver CBTi programs, brief versions of CBTi, or single components of CBTi such as Stimulus Control Therapy [100–102]. A 2019 systematic review [103] of 13 studies investigating the delivery of CBTi in primary care settings reported that multi-component CBTi is an effective treatment in primary care. Ongoing Australian research aims to investigate the delivery of CBTi by Australian practice nurses [104]. A small number of studies have also investigated digital CBTi referral pathways in Australian primary care [75, 105, 106]. Between 2022 and 2026, the ASA led two Commonwealth-funded grant programs that provided sleep health and insomnia education to over 35 000 general practitioners, psychologists, nurses, and pharmacists, to improve access and use of evidence-based sleep disorder resources, including access to CBTi.

To improve access to CBTi, clinicians may also deliver treatment via tele-health (e.g. to reduce access issues related to rurality/location) or deliver CBTi concurrently to small groups of patients. CBTi delivered via tele-health is an effective modality [107]. However, this does not improve equitable access to CBTi for all people in rural/remote locations (e.g. those with limited internet access, sleep interventions tailored for First Nations Peoples in rural/remote locations; see Supplement).

#### Digital CBTi

CBTi programs delivered by trained therapists have also been translated into digital CBTi programs with empirical support [69, 71, 108]. Meta-analyses of digital CBTi programs have generally reported moderate improvements in insomnia severity, depressive symptoms, and sleep parameters, sustained by long-term follow-up [69, 71, 108, 109]. A 2016 meta-analysis by Zachariae and colleagues and a 2020 meta-analysis by Soh and colleagues reported that digital CBTi is similarly effective to face-to-face CBTi [71, 108]. Recent network meta-analyses have compared different modalities of CBTi delivery (including face-to-face, group, and digital CBTi programs with/without a real or virtual therapist) [110–112]. CBTi modalities were found to be more effective overall than control conditions, with few significant differences between specific modalities across settings [112], and the important role of digital CBTi was recognized due to opportunities for scalability and cost-effectiveness [110–112]. It is important to support and scale up access to all CBTi modalities in the Australian health system.

There are several Australian digital CBTi programs with emerging scientific evidence that are available to consumers online or via research studies (Table 6). Australian-developed programs with peer-reviewed evidence include This Way Up: Insomnia Program [72, 113], Bedtime Window [76, 77, 114], Sleep Ninja [115–117], Sleep Fix [74, 75], A Mindful Way [78], and the Sleep Course [118]. These programs differ in; cost, availability, tailored vs static delivery of CBTi components guided by underlying algorithms, the specific CBTi components that are included, the sequence they are presented in, the degree of digital interactive and personalized components, and requirement for clinician involvement versus self-guided availability. There is also a very large number of apps/digital programs available online that claim to treat insomnia symptoms, which have little or no scientific evidence [119, 120]. It is important to consider digital CBTi programs with specific scientific evidence (rather than programs that are “based on” scientific evidence or CBTi principles).

**Table 6:** Digital CBTi programs available in Australia

Program	Peer-reviewed evidence	CBTi sessions and component/s	Target age	Tailored/personalized recommendations ^*^	Guided vs unguided^	Access	Cost
**Australian programs**							
A Mindful Way	Kennett et al. [78]	6-session static mindfulness + CBTi program.	Adults	Static	Both	Website	At cost
Bedtime Window	Sweetman et al. [77] Sweetman et al. [76] Sweetman et al. [114]	5-session interactive multi-component CBTi program.	Adults	Personalized	Both	Website and clinical trials	Free via research studies
Sleep Fix	Aji et al. [74] Aji et al. [155] Gordon et al. [75]	App-based interactive bedtime restriction therapy program.	Adults	Personalized	Self-guided	Clinical trials	Free via research studies
Sleep Ninja	Werner-Seidler et al. [117] Werner-Seidler et al. [115] Werner-Seidler et al. [116]	6-week interactive app-based self-guided CBTi program. Does not include sleep restriction therapy.	Adolescents	Personalized	Self-guided	App stores	Free
The Sleep Course	Scott et al. [118]	4-session static CBTi program with weekly clinician guidance.	Adults	Static	Clinician-guided	Website and clinical trials	Free
This Way Up: Insomnia Program	Mason et al. [113] Mahoney et al. [73] Grierson et al. [72]	4-session static multi-component CBTi program.	Adults	Static	Both	Website	Free
**International programs available in Australia**							
CBT-I Coach	Kuhn et al. [156] Koffel et al. [157]	App-based CBTi program intended to be used alongside care from a health professional.	Adults	Personalized	Clinician-guided	App stores	Free
Sleep Reset	Gorovoy et al. [158]	12-week app-based interactive digital CBTi program.	Adults	Personalized	Clinician-guided	Website and app stores	At cost
Headspace Sleep App^**^	Staiano et al. [159]	18 daily sessions. Mindfulness and relaxation content combined with CBTi. Does not include sleep restriction therapy.	Children, Adolescents, Adults	Static	Self-guided	App stores	At cost

Australian and international programs listed by alphabetical order.

This information will be kept up to date on https://sleepcentral.org.au/Central/Central/Contents/Referral-digital-CBTi.aspx.

Users are encouraged to conduct their own research of the above programs for safety, effectiveness, costs, and availability.

There may be other evidence-based digital CBTi programs available in Australia.

^*^Tailored/personalized recommendations refers to programs that include algorithms to guide automatic personalized recommendations (e.g. tailored bedtime restriction therapy).

^Guided CBTi indicates programs that require or include optional involvement of a healthcare provider.

^**^The Headspace sleep application listed here is produced by a different organization from Headspace National Young Mental Health Foundation (Australia).

Although digital CBTi programs are expected to rapidly increase availability of CBTi, they may not be appropriate for all people with insomnia disorder. For example, some people with insomnia may be more appropriate for management with a clinician-guided digital CBTi program, while others may be more appropriate for personalized treatment from a clinician with CBTi training (e.g. those with contra-indications to self-guided bedtime restriction therapy, such as unmanaged epilepsy, bipolar, schizophrenia, and/or excessive daytime sleepiness).

### When psychological and behavioral approaches are not effective

Although CBTi is recommended as the first line treatment for chronic insomnia, approximately 20%–30% of patients do not report clinically significant improvement of insomnia severity following CBTi, and approximately 40% do not achieve complete remission of insomnia [87, 121–123]. Before “switching” to an alternative “second-line” psychological/behavioral therapy or commencing a sleep medication, it can be helpful to consider the underlying reasons for inadequate response to CBTi.

First, careful assessment of additional underlying precipitating factors that are contributing to persistent insomnia may be appropriate in patients that do not respond to CBTi (e.g. the onset of comorbid conditions, injury, hospitalization, or travel between time zones and jet lag experienced during CBTi may reduce adequate treatment response). Recommendation for a sleep physician or sleep study referral may be indicated in patients with a suspected high risk of a comorbid sleep disorder that could be presenting as insomnia symptoms. Some comorbid sleep conditions (e.g. sleep apnea, circadian rhythm sleep–wake disorders) may have initially been presenting as insomnia, and CBTi may not be indicated [124].

Second, limited treatment-response may also depend on the specific treatment that the patient has accessed and used. For example, CBTi is often conflated with simple “sleep hygiene” recommendations (see supplement and Table 5) [63, 124]. Recommendations about healthy sleep behaviors are not an effective stand-alone treatment for chronic insomnia [83], despite being widely used for this purpose [33]. In patients who report limited benefit from CBTi, it can therefore be important to discuss the specific CBTi strategies/treatments that were used, including whether bedtime restriction therapy or stimulus control therapy recommendations were provided. If it becomes apparent that “sleep hygiene” recommendations were provided in the absence of these behavioral treatment components, it may be appropriate to provide education and refer patients to access a multi-component CBTi intervention.

Finally, some patients who are referred for CBTi may find it difficult to enact treatment recommendations or may not complete full CBTi programs, resulting in a blunted response to CBTi. For example, patients may not enact specific behavioral therapy recommendations to reduce time in bed (bedtime restriction therapy) or get out of bed at a consistent time each morning regardless of the amount of sleep on the previous night (stimulus control therapy). Some patients referred to clinician-delivered or digital CBTi programs may not complete/attend all recommended sessions. This reduced adherence/engagement/completion of CBTi may limit therapeutic response. Clinicians may work with patients on a case-by-case basis to identify any barriers to engagement, access, and adherence, and consider whether motivational support, tailored CBTi approaches, treatment from another clinician/program, involvement of bed partners/family members, or other troubleshooting support may be beneficial, before considering alternative second-line therapies.

Selected patients who do not respond to multi-component CBTi may benefit from recommendations for alternative psychological and behavioral therapies (see Supplement), or sleep medication use. If sleep medications are used, the required duration of use may not be known initially; therefore, ongoing monitoring and periodic medication withdrawal and/or dose minimization is recommended. Regular review and monitoring for side effects and adherence to the prescribed dosage is essential. Medication tolerance may develop and the magnitude of this problem is not established in controlled trials. Limited controlled trials for “z-drugs” and dual orexin receptor antagonists over a 6-month period have shown favorable durability [125, 126]. Choice of hypnotic should be individualized; however, shorter acting agents and those with evidence for more extended usage may be considered more appropriate [127, 128].

More research is required regarding the effectiveness and safety of long-term sleep medication use, and combination of pharmacological approaches with psychological and behavioral approaches. A 2026 clinical practice guideline of the American Academy of Sleep Medicine (AASM) [129] investigated the evidence in adults for the efficacy of combination treatment for chronic insomnia with CBTi and pharmacotherapy, compared with pharmacotherapy or CBTi alone. The evidence supported the use of combination therapy over pharmacotherapy alone; however, the evidence did not support the use of combination therapy over CBTi alone.

## Pharmacological Management

Information about medications commonly used for insomnia in Australia from the Australian Medicines Handbook (AMH), Therapeutic Guidelines (TG), Therapeutic Goods Administration (TGA), and PBS is presented in Table 7. The overall ASA recommendations for pharmacological management of insomnia disorder are presented below and in Table 1. Detailed information about the mechanisms, scientific evidence, and recommendations for benzodiazepines, “z-drugs,” melatonin, dual orexin receptor antagonists, antidepressants, antipsychotics, sedating antihistamines, cannabinoids, and complementary medications is presented in the Supplement. Although not recommended for insomnia, information on antidepressants, antipsychotics, and sedating antihistamines are included as these medications are commonly used for insomnia management (e.g. in the context of comorbid insomnia and mental health symptoms). The information provided in this resource refers to the use of these medications for management of insomnia (i.e. not for other indications).

**Table 7:** Guideline recommendations for medications commonly used for insomnia management in Australia

Name (available tablet strength)	Indication for insomnia (AMH, TG, TGA)	Category (schedule)	PBS listing (adult insomnia)	Therapeutic dose range for insomnia	Common side effects in AMH	Recommended duration for insomnia in AMH
**Benzodiazepines**						
Temazepam (10 mg)	AMH: Yes (short-term) TG: Yes (<2 weeks)	Prescription only (Schedule 4)	25 tablets 0 repeats	Adult, 10–20 mg at night Elderly, 5–10 mg at night	Relevant to all benzodiazepines: Drowsiness, oversedation, light-headedness, hypersalivation, ataxia, slurred speech, effects on vision (e.g. blurred vision, impaired tracking) Dependence; use of benzodiazepines can lead to physical and psychological dependence (even if used for short periods), tolerance, and misuse	Reserve for short-term (2–4 weeks) or intermittent use only; they should be part of a broader treatment plan, not a first or sole treatment Withdrawal syndrome may occur on stopping, particularly if taking high doses; prevent or alleviate by gradual dose reduction
Nitrazepam (5 mg)	AMH: Yes TG: No	Prescription only (Schedule 4)	25 tablets 0 repeats	Adult, 5–10 mg at night Elderly, 2.5–5 mg at night		
Lorazepam (0.5 mg, 1 mg, 2.5 mg)	AMH: Yes (short-term, associated with anxiety) TG: No	Prescription only (Schedule 4)	No	Adult, oral 1–2 mg at night		
Clobazam (10 mg)	AMH: Yes (short-term, associated with anxiety) TG: No	Prescription only (Schedule 4)	No	Adult, 10–30 mg at night Elderly, 10–20 mg at night		
Flunitrazepam (1 mg)	AMH: Yes TG: No *Specific TGA detail: Severe cases*	Controlled drugs (Schedule 8)	No	Adult, 0.5–2 mg at night Elderly, 0.5–1 mg at night		As above. Withdrawal can be severe if used for >1 week
Oxazepam (15 mg, 30 mg)	No	Prescription only (Schedule 4)	No	NA		NA
Diazepam (2 mg, 5 mg)	No	Prescription only (Schedule 4)	No	NA		NA
Bromazepam (3 mg, 6 mg)	No	Prescription only (Schedule 4)	No	NA		NA
Alprazolam (0.25 mg, 0.5 mg, 1 mg, 2 mg)	No	Controlled drugs (Schedule 8)	No	NA		NA
**Z-drugs**						
Zolpidem (10 mg IR) (6.25 mg, 12.5 mg CR)	AMH: Yes (short-term) TG: Yes (<2 weeks)	Prescription only (Schedule 4)	No	Adult, 5–10 mg IR or 6.25–12.5 mg CR immediately before bed See AMH for dosing in special populations	Diarrhea, impaired alertness the next Morning; dependence, tolerance, and misuse comparable to benzodiazepines can occur	Use the lowest possible dose Short-term only (zopiclone specified as up to 2 weeks) Withdrawal syndrome may occur on stopping, particularly if taking high doses; prevent or alleviate by gradual dose reduction
Zopiclone (7.5 mg)	AMH: Yes (short-term) TG: Yes (<2 weeks) *Specific TGA detail:* *7–14 days*	Prescription only (Schedule 4)	Repatriation only (short-term for insomnia) 14 tablets (1 repeat) or 30 tablets (0 repeats)	Adult, 3.75–7.5 mg at night See AMH for dosing in special populations	Taste disturbance, dry mouth, drowsiness, impaired alertness the next morning; dependence, tolerance, and misuse comparable to benzodiazepines can occur	
**Dual orexin receptor antagonists**						
Suvorexant (15 mg, 20 mg)	AMH: Chronic insomnia TG: Yes (up to 3 months then review)	Prescription only (Schedule 4)	No	Adult <65 years, 20 mg (30 min before bed) Adult >65 years, 15 mg (30 min before bed)	Somnolence, headache	Assess response to treatment after 7–10 days; reassess need for continuation after 3 months
Lemborexant (5 mg, 10 mg)	AMH: Chronic insomnia TG: Not listed	Prescription only (Schedule 4)	No	Adult, 5–10 mg at night *See AMH for dosing in special populations*	Somnolence, headache, nightmare or abnormal dreams, sleep paralysis	Use at the lowest dose for the shortest possible duration
**Melatonin**						
Melatonin (2 mg CR)	AMH: Yes (short-term) TG: Yes (for up to 13 weeks then review)	Pharmacist only (Schedule 3) if ≥55 years. Prescription only (Schedule 4). *See current Poisons Standard for details*	No	>55 years, 2 mg at night, 1–2 hours before bed	Back pain, arthralgia	Up to 13 weeks
**Antihistamines**						
Doxylamine (25 mg)	AMH: Yes (for short-term use) TG: No	Pharmacist only (Schedule 3)	No	Adult, 25–50 mg (30 min before bed)	Sedation, psychomotor impairment, dizziness, confusion, headache, blurred vision, mydriasis, dry eyes, constipation, dry mouth, urinary retention	Sedating antihistamines are generally not recommended for the treatment of insomnia; do not use for over 10 consecutive days, sedative effect declines quickly with continued use
Diphenhydramine (50 mg)	AMH: Yes (for short-term use) TG: No	Pharmacist only (Schedule 3)	No	Adults, 50 mg at night		
**Antidepressants (not indicated for insomnia)**						
Mirtazapine	No	Prescription only (Schedule 4)	No	NA	See Supplement	NA
Amitriptyline	No	Prescription only (Schedule 4)	No	NA	See Supplement	NA
**Antipsychotics (not indicated for insomnia)**						
Quetiapine	No	Prescription only (Schedule 4)	No	NA	See Supplement	NA
Olanzapine	No	Prescription only (Schedule 4)	No	NA	See Supplement	NA

AMH: Australian Medicines Handbook, TG: Therapeutic Guidelines (Insomnia in adults) TGA: Therapeutic Goods Administration (only included if specific detail not captured otherwise by AMH or TG), PBS: Pharmaceutical Benefits Scheme. IR: immediate release, CR: controlled release. Not exhaustive list of all side-effects or less common side effects. For full list of side effects, see product information for each medication. See Supplement for further information on the effectiveness, side effects, and overall recommendations for each medicine class. AMH, TG, TGA, and PBS information are developed independently and may be contradictory in specific circumstances. Information from AMH, TG, and TGA accessed from June 2025 to February 2026.

Although CBTi is the recommended “first-line” treatment for insomnia, it does not result in immediate improvements in sleep, can be difficult to access, and is not effective in all people with insomnia. Consequently, alternative or concurrent treatment with sleep medications may be appropriate in some patients. For example, people experiencing significant distress or functional impairment due to their insomnia (which requires more immediate resolution), short-term insomnia resulting from an obvious underlying precipitating factor (as opposed to psycho-behavioral perpetuating factors that are a primary target of CBTi), and in cases where CBTi is not appropriate or effective.

Sleep medications may be prescribed after careful consideration of potential benefits and risks of side-effects, adverse events, informed patient treatment preferences and treatment priorities, and previous use of sleep medications, against the potential health risks of not treating insomnia [54]. When prescribed, sleep medications should ideally be limited to the lowest effective dose, for the shortest possible duration (see Table 7), and with specific tapering and withdrawal strategies in place, including access to CBTi to support discontinuation of medications.

Sleep medications do not address the underlying psychological and behavioral factors thought to perpetuate chronic insomnia. Sleep medications are generally not recommended for long-term use due to risks of side-effects, adverse events, and dependence [54]. Patients should be fully informed of the potential risks of dependence and side effects of specific medications before they are prescribed. People presenting for management of chronic insomnia in Australia may also have a history of sleep medication use and be resistant to deprescribing approaches or psychological and behavioral therapies. For example, sleep medications which may have initially caused rapid symptom relief may be perceived as an effective long-term management approach, some patients may fear insomnia relapse in the presence of attempts to reduce medication use (that may be conflated with withdrawal symptoms), and other patients may not have knowledge or confidence in CBTi as an effective treatment in the presence of sleep medications [130]. Each consultation represents an opportunity to support gradual changes in readiness to consider psychological and behavioral approaches for insomnia in patients with long-standing patterns of sleep medication use for insomnia. See the Resources and Training section below for guidelines and clinical resources to support deprescribing of medications used for insomnia.

## Insomnia in Specific Populations

Insomnia may occur more frequently in specific populations in Australia, who may also require tailored assessment and management approaches. The Supplement includes specific information on the prevalence and management of insomnia in the following peoples/contexts; First Nations Peoples, shift workers, perinatal insomnia, comorbid insomnia and obstructive sleep apnea, perimenopause and menopause, older adults, and insomnia occurring in the context of cancer. This is not an exhaustive list of specific populations/contexts that more frequently present with insomnia. The information presented in each section of the supplement aims to provide brief context and peer-review references, to facilitate further investigation.

## Resources and Training

Several evidence-based resources on insomnia assessment and management are freely available for Australian consumers and clinicians (Table 8). The *Australasian Sleep Association* is a not-for-profit peak scientific body in Australia and New Zealand representing clinicians, scientists, and researchers working in sleep health and sleep medicine, which promotes and provides education and training to members and the broader health community. The ASA also fosters scientific research and establishes best-practice clinical guidelines. The *Sleep Health Foundation* is a community-facing not-for-profit health promotion charity that aims to raise community awareness about the value of sleep, how to improve it, and how to address common sleep disorders. The *Australian Psychological Society* is the peak body for psychologists in Australia, committed to advancing mental health and wellbeing through advocacy, innovation, and excellence in psychological science and practice. The APS promotes CBTi as the most effective psychological treatment for chronic insomnia and supports its use through professional development, clinical resources, and public education. The *Royal Australian College of General Practitioners* is the peak professional body for Australian general practitioners. The RACGP and ASA have collaborated on several general practitioner education initiatives including articles, online modules, conference sessions, online guidelines, and brief on-the-spot resources. The *Australian Primary Health Care Nurses Association* is the peak professional body for nurses working in primary health care and has collaborated with the ASA on nurse sleep education resources, conference exhibitions, and workshops. The *Pharmaceutical Society of Australia* is the peak professional body representing Australian pharmacists. The ASA and PSA have also collaborated on a 2-year pharmacist insomnia education program that has resulted in the development and implementation of numerous sleep education resources for Australian pharmacists.

**Table 8:** Insomnia resources for consumers and clinicians

Resources for consumers	
	Sleep Health Foundation, https://www.sleephealthfoundation.org.au/ Sleep Central, CBTi provider directory, https://sleepcentral.org.au/Central/Contents/Clinicians-CBTi.aspx Digital CBTi programs with scientific evidence (see Table 6 and https://sleepcentral.org.au/Central/Central/Contents/Referral-digital-CBTi.aspx). Patient decision aids may support shared decisions between patients and clinicians when discussing different insomnia management approaches (e.g. see Cheung et al. [160] Patient Decision Aid for insomnia medication, face-to-face CBTi, and digital CBTi).
**Resources for clinicians**	
Australasian Sleep Association	
	ASA Learning Centre includes many webinars and presentations on insomnia management, https://www.sleep.org.au/Public/Public/TopClassAccess/LearningCentre.aspx?hkey=f0504aa8-fac5-46a4-9fde-65ebe555bf45 Sleep Central, www.sleepcentral.org.au ∘ CBTi provider directory, https://sleepcentral.org.au/Central/Contents/Clinicians-CBTi.aspx ∘ Sleep Health Action Plan: https://sleep.org.au/Central/Central/Contents/SH-action-plan/SH-Action-Plan-landing.aspx?hkey=dfb33c37-d37e-44cc-a13f-003ce62ce1bb ∘ Digital CBTi programs: https://sleepcentral.org.au/Central/Central/Contents/Referral-digital-CBTi.aspx
Australian Psychological Society	
	The APS and the Australasian Sleep Association offer a 6-hour CBTi certified skills course (for APS members and non-members), https://psychology.org.au/event/24371 The APS *Evidence-based psychological interventions in the treatment of mental disorders: A literature review* (insomnia chapter), https://psychology.org.au/for-the-public/psychology-topics/evidence-based-psychological-interventions Insomnia disorder practice guide (for APS members) https://psychology.org.au/for-members/resource-finder/resources/assessment-and-intervention/clinical-guide/insomnia-disorder-practice-guide Sleep diary (for APS members), https://psychology.org.au/for-members/resource-finder/resources/for-your-clients/tools-and-worksheets/sleep-diary-worksheet
Royal Australian College of General Practitioners	
	RACGP: Prescribing drugs of dependence in general practice: Benzodiazepines guideline: https://www.racgp.org.au/clinical-resources/clinical-guidelines/key-racgp-guidelines/view-all-racgp-guidelines/drugs-of-dependence/part-b Australian Journal of General Practice, special issue on sleep medicine, https://sleep.org.au/Public/Public/News/Articles/June/AJGP.aspx RACGP on-demand gpLearning modules (accessible to RACGP members). Brief behavioral therapy for insomnia resource: https://www.racgp.org.au/clinical-resources/clinical-guidelines/handi/handi-interventions/cogntive-and-behavioural-therapies/brief-behavioural-therapy-insomnia-in-adults General Practice Mental Health Standards Collaboration training: https://gpmhsc.org.au/InfoSection/Index/c4dbd3ef-e7bd-48c3-9a73-0e0f85372111
Pharmaceutical Society of Australia	
	Quality use of medicines for insomnia and sleep health education curriculum: https://www.psa.org.au/qum-qumish/ The Australian Pharmaceutical Formulary and Handbook – Insomnia treatment guideline for pharmacists. https://apf.psa.org.au/treatment-guidelines-pharmacists/insomnia
American Academy of Sleep Medicine	
	American Academy of Sleep Medicine: Insomnia Toolkit for Clinicians. https://aasm.org/clinical-resources/insomnia-toolkit/
HealthEd	
	Podcasts series on insomnia management, https://www.healthed.com.au/clinical_articles/insomnia-assessment-and-management-podcast-series/
Other sleep medication resources	
	Australian Medicines Handbook (login required). https://amhonline.amh.net.au/auth Therapeutic Guidelines (login required). https://www.tg.org.au/ The Maudsley deprescribing guidelines [161]

The Sleep Health Foundation (SHF) plays a vital role in raising public awareness about the importance of sleep and provides accessible, evidence-based information about sleep, sleep disorders, and pathways to treatment across Australia. With over 50 000 monthly website visitors and more than 300 000 annual downloads of fact sheets, the SHF is a trusted and widely used resource. Its weekly national media outreach further amplifies its impact, ensuring accurate sleep health messages reach diverse audiences. As sleep-deprived individuals are especially vulnerable to misinformation [131, 132], the SHF’s independence is crucial in maintaining impartiality. Government funding is essential to sustain this trusted source of consumer-focused information. Supporting the foundation aligns with public health priorities by promoting sleep health literacy and empowering individuals to improve sleep, improve their health, and reduce the burden of disease in Australia. All levels of government should recognize the immense value of this work and the importance of ensuring its continuity.

Since 2022, the ASA has led two government grants to improve the management of insomnia in Australian primary care. Grants received through the Health Peak and Advisory Bodies (HPAB) program and the Quality Use of Diagnostics, Therapeutics and Pathology program have enabled ASA to work in collaboration with the RACGP, APS, APNA, PSA, SHF, and university and primary care organizations to improve the knowledge and confidence of primary care clinicians in identifying, assessing, and managing insomnia [133]. During these programs, approximately 35 000 Australian primary care clinicians have accessed evidence-based information on preventive sleep health and clinical sleep disorder management, an online primary care sleep health education website (sleepcentral.org.au) has been visited over 90 000 times, and an estimated 40 000 clinicians have accessed primary care sleep education articles.

## Conclusion

Insomnia is a prevalent and debilitating disorder in Australian adults that can be experienced for an acute or chronic duration. Insomnia commonly co-occurs alongside other mental, physical, and sleep disorders; however, there is strong evidence that insomnia should be viewed as a “comorbid disorder” that warrants specific assessment and treatment attention. CBTi is a safe, effective, and durable approach to manage insomnia in a diverse range of patient presentations and when delivered via different modalities. Treatment of insomnia not only improves sleep and daytime function but also often results in improved outcomes for comorbid mental and physical conditions. Without treatment, insomnia is a chronic condition that leads to inordinate health and economic burden. Accordingly, we appeal to health professionals to prioritize insomnia assessment and management in their clinical practice. Policy-makers are also encouraged to consider pathways to improve access to CBTi in the Australian health system.

Specific recommendations include using screening questionnaires, sleep diaries, and structured interviews for assessing and diagnosing insomnia. Overnight sleep studies are indicated when comorbid sleep disorders are suspected.

Clinicians should consider CBTi as the “first-line” treatment option for insomnia, and discuss CBTi with all patients with insomnia. In-person CBTi delivered by clinicians such as psychologists with training and expertise in insomnia management is considered the “gold standard”; however, access to clinicians with sleep expertise is limited by long waitlists, high costs, or other barriers [58, 134]. CBTi delivered by a psychologist or general practitioner via tele-health is supported by the Better Access initiative, or treatment can be accessed online through several evidence-based Australian digital CBTi programs. Other psychological and behavioral treatments (e.g. mindfulness, acceptance and commitment therapy) show promise as an adjunct or alternative to CBTi following non-response.

If acute insomnia is causing significant distress and functional impairment, is caused by an obvious underlying precipitating factor, and if CBTi is not available, effective, or appropriate, then short-term use of an indicated sleep medication may be appropriate. Before sleep medications are prescribed, clinicians should work with patients to collaboratively weigh the potential risks of pharmacotherapy dependence, side-effects, and adverse events, against the health risks of not treating insomnia. If used, specific benzodiazepines and “z-drugs” are indicated for short-term use, dual orexin receptor antagonists and melatonin have longer-term safety and efficacy data (see Table 7). CBTi is effective in patients using sleep medications and may facilitate deprescribing.

To improve insomnia outcomes in Australia, clinicians and consumers should make use of evidence-based resources on insomnia assessment and management that have been developed by, and made freely available though, the Australasian Sleep Association, the Sleep Health Foundation, the Australian Psychological Society, the Royal Australian College of General Practitioners, the Australian Primary Health Care Nurses Association, the Pharmaceutical Society of Australia, and other sleep and primary care organizations.

## Supplementary Material

Sup_clean_zpag086

## Data Availability

This is a review manuscript—no primary data were collected or are available.

## Abbreviations

AMH, Australian Medicines Handbook; APS, Australian Psychological Society; APNA, Australian Primary Health Care Nurses Association; ASA, Australasian Sleep Association; CBTi, Cognitive behavioral therapy for insomnia; GP, General practitioner; MBS, Medicare Benefits Schedule; PBS, Pharmaceutical Benefits Scheme; PSA, Pharmaceutical Society of Australia; RACGP, Royal Australian College of General Practitioners; SHF, Sleep Health Foundation; TGA, Therapeutic Goods Administration; TG, Therapeutic Guidelines
