# Supplementary material for: Management of adult insomnia in Australia: a joint position statement of the Australasian Sleep Association, Sleep Health Foundation, Royal Australian College of General Practitioners, Australian Psychological Society, Pharmaceutical Society of Australia, and Australian Primary Health Care Nurses Association
Source: Sleep Adv. 2026 Jul 29;7(3):zpag086. doi: 10.1093/sleepadvances/zpag086 (PMC13600392; doi:10.1093/sleepadvances/zpag086)
Supplement: Supplementary file 1 [file sup_clean_zpag086.docx]

Supplementary materials

**Title:**

Management of adult insomnia in Australia.

A joint position statement of the Australasian Sleep Association, Sleep Health Foundation, Royal Australian College of General Practitioners, Australian Psychological Society, Pharmaceutical Society of Australia, and Australian Primary Health Care Nurses Association.

**Authors:**

Alexander Sweetman^a,b,c*^, Stacey Putland^a^, Hailey Meaklim^e,d,f^, Sara Winter^g,h,i^, Gerard Kennedy^j^, Leon Lack^f^, Delwyn Bartlett^k^, James Szeto^l^, Sarah Blunden^m,n^, Henrique Salles^o,l^, Vikki White, Amelia Scott^k,p^, Cele Richardson^b^, Nicole Grivell^e,f,q^, Jenny Haycock^f^, Daniel Sullivan^i,r,s^, Erin Oldenhof^l,t^, Shani Pickering^u^, Moira Junge^v,w^, Bianca Cannon^x,y^, Linda De George-Walker^z^, Yaqoot Fatima^aa^, Wayne Williams^bb^, Eron Cripps^cc^, Janet M.Y. Cheung^dd^, Camilla Hoyos^k^, Darren Mansfield^ee^, Joel Aizenstros^w,ff^, David Cunnington^gg^, Melissa Ree^hh^

**Affiliations:**

a. Australasian Sleep Association, Sydney Australia.
b. School of Psychological Science, University of Western Australia, Australia.
c. Bond University, Gold Coast, Queensland, Australia.
d. University of Melbourne, Melbourne, Victoria, Australia.
e. Institute for Breathing and Sleep, Austin Health, Melbourne, Victoria, Australia
f. Flinders University, Flinders Health and Medical Research Institute, Sleep Health, Bedford Park, South Australia, Australia.
g. Allied Health Research Collaborative, The Prince Charles Hospital, Queensland, Australia.
h. Faculty of Medicine, The University of Queensland, Queensland, Australia.
i. School of Applied Psychology, Griffith University, Queensland, Australia.
j. School of Health and Biomedical Sciences, College of Science, Engineering & Health, RMIT University, Bundoora, Victoria, Australia.
k. Centre for Sleep and Chronobiology, Woolcock Institute of Medical Research, Macquarie University, Sydney, New South Wales, Australia
l. Reconnexion, Melbourne, Victoria, Australia.
m. Appleton Institute of Behavioural Science, Central Queensland University, Australia.
n. School of Educational Psychology and Counselling, Monash University, Melbourne, Australia.
o. Level Up Psychology, Melbourne, Victoria, Australia.
p. School of Psychological Sciences, Macquarie University, Sydney, New South Wales, Australia.
q. Department of Physiotherapy, Melbourne School of Health Sciences, University of Melbourne, Heidelberg, Victoria, Australia.
r. Thompson Institute, University of the Sunshine Coast, Queensland, Australia.
s. Child Health Research Centre, University of Queensland, Queensland, Australia.
t. School of Psychology, Deakin University, Melbourne, Victoria, Australia.
u. Pharmaceutical Society of Australia, Australia.
v. Sleep Health Foundation, Australia.
w. Monash University, Melbourne, Victoria, Australia.
x. Royal Australian College of General Practitioners, Australia.
y. University of Sydney, Sydney, New South Wales, Australia.
z. Australian Psychological Society, Australia.
aa. University of the Sunshine Coast, Queensland, Australia.
bb. Elder, Specialist Principal Supervisor Indigenous Health Education, Academic Lead Indigenous Health, The University of Queensland Rural Clinical School, Australia.
cc. Consumer representative, Australia.
dd. School of Pharmacy, Faculty of Medicine and Health, The University of Sydney, Sydney, New South Wales, Australia.
ee. Epworth Sleep Centre, Melbourne, Victoria, Australia.
ff. Victoria Clinic, Melbourne, Victoria, Australia.
gg. Sunshine Coast Respiratory and Sleep, Queensland, Australia.
hh. Sleep Matters, Perth, Western Australia, Australia.

***Corresponding Author:**

Dr Alexander Sweetman, Senior Program Manager, Australasian Sleep Association.
Email: [alexander.sweetman@sleep.org.au](mailto:alexander.sweetman@sleep.org.au); [dr.alexander.sweetman@gmail.com](mailto:dr.alexander.sweetman@gmail.com).

**Contents**

[1.	Development and aims of this position statement	4](#_Toc235947240)

[2.	Regulatory bodies and universal health system	4](#_Toc235947241)

[3.	Differential diagnoses	5](#_Toc235947242)

[3.1.	Comorbid insomnia	8](#_Toc235947243)

[3.1.1.	Comorbid mental health conditions	8](#_Toc235947244)

[3.1.2.	Comorbid chronic pain and physical health conditions	8](#_Toc235947245)

[3.1.3.	Comorbid sleep disorders	8](#_Toc235947246)

[4.	Cognitive Behavioral Therapy for insomnia (CBTi)	9](#_Toc235947247)

[4.1.	CBTi for acute insomnia	9](#_Toc235947248)

[4.2.	CBTi effects in comorbid insomnia	10](#_Toc235947249)

[4.3.	CBTi in patients using sleep medications	11](#_Toc235947250)

[4.4.	CBTi risks and side-effects	11](#_Toc235947251)

[4.5.	Stepped care models for insomnia	12](#_Toc235947252)

[4.6.	Other psychological and behavioral approaches	13](#_Toc235947253)

[4.6.1.	Healthy sleep habits (Sleep hygiene)	13](#_Toc235947254)

[4.6.2.	Mindfulness based therapy for insomnia	14](#_Toc235947255)

[4.6.3.	Intensive sleep retraining	14](#_Toc235947256)

[4.6.4.	Acceptance Commitment Therapy	15](#_Toc235947257)

[5.	Pharmacological management of insomnia	15](#_Toc235947258)

[5.1.	Benzodiazepines	16](#_Toc235947259)

[5.2.	Z-drugs	17](#_Toc235947260)

[5.3.	Melatonin	17](#_Toc235947261)

[5.4.	Dual orexin receptor antagonists	19](#_Toc235947262)

[5.5.	Sedating antihistamines	19](#_Toc235947263)

[5.6.	Antidepressant medications	20](#_Toc235947264)

[5.7.	Antipsychotic medications	21](#_Toc235947265)

[5.8.	Cannabinoids	21](#_Toc235947266)

[5.9.	Complementary and alternative medications	22](#_Toc235947267)

[6.	Insomnia in specific populations	23](#_Toc235947268)

[6.1.	First Nations Peoples	23](#_Toc235947269)

[6.2.	Shift work	24](#_Toc235947270)

[6.3.	Perinatal insomnia	25](#_Toc235947271)

[6.4.	Comorbid insomnia and sleep apnea	26](#_Toc235947272)

[6.5.	Older adults	26](#_Toc235947273)

[6.6.	Perimenopause and Menopause	27](#_Toc235947274)

[6.7.	Cancer	27](#_Toc235947275)

[7.	References	29](#_Toc235947276)

# 1. Development and aims of this position statement

This Position Statement was developed in 2024-2026 by representatives of the Australasian Sleep Association (ASA), Sleep Health Foundation (SHF), Royal Australian College of General Practitioners (RACGP), Australian Psychological Society (APS), Pharmaceutical Society of Australia (PSA), and Australian Primary Health Care Nurses Association (APNA). Development of this Position Statement was supported by an Australian Commonwealth Government Health Peak and Advisory Bodies grant and a Quality Use of Diagnostics, Therapeutics and Pathology grant coordinated by the ASA. Perspectives of sleep researchers, psychologists, sleep and respiratory physicians, general practitioners, pharmacists, nurses, advocacy and implementation experts, and people with lived experience of insomnia are represented.

This Position Statement aims to update and expand on a ASA Insomnia Position Statement published in 2017^1^, and provide Australian healthcare staff with updated information on the prevalence, aetiology, consequences, assessment and management of insomnia in adults. Although international evidence-based insomnia guidelines and consensus statements have been developed^2-5^, there is a need for an updated Australian-specific insomnia position statement. This Position Statement provides contextualized information, addressing the unique features of insomnia management in Australia, including; the current landscape of insomnia management in Australia^6-8^, perspectives of people with insomnia^9,10^, experiences and scope of practice of different Australian clinicians^11-14^, the Australian health system (e.g., Medicare, the universal publicly-funded health system in Australia that provides rebates for medical services and procedures), Australian regulatory bodies overseeing availability and access to diagnostics and treatments (e.g., Therapeutic Goods Administration [TGA], Medical Services Advisory Committee, Pharmaceutical Benefits Advisory Committee, etc.), Australian organisations for professional sleep and primary care education^6^, advocacy, and consumer representation, specific socio-demographic and cultural groups in Australia, and clinician education programs^15,16^ and treatment options specific to the Australian health system. Literature searches occurred from November 2024 to February 2026, prioritizing evidence from existing guidelines and position statements, systematic reviews and meta-analyses, randomized controlled trials, recent peer-reviewed literature, and Australian-specific data where available. The statement was initially reviewed by the ASA Clinical Committee and ASA Board. Following ASA approval, the statement was independently reviewed by the SHF, RACGP, APS, PSA, and APNA and feedback was provided and integrated over several rounds of revision. The ASA Board and author team (including representatives of all organisations) were responsible for consolidating feedback into the final version.

# 2. Regulatory bodies and universal health system

The Therapeutic Goods Administration (TGA) is a division of the Australian Government Department of Health, Disability and Ageing that is responsible for ensuring the safety, efficacy, and quality of medicines, and medical devices. It evaluates and monitors these products before they can be legally supplied, setting standards for manufacturing, labelling, and advertising (See description of TGA approval for medications listed in the Supplement).

The Medicare Benefits Schedule (MBS) is a government program that lists medical services subsidized by Medicare, Australia’s public healthcare system. It covers a wide range of consultations, diagnostic tests, and procedures, allowing patients to access essential healthcare at reduced cost or no cost. In the context of insomnia, MBS items may subsidise the cost of consultations with general practitioners, psychologists, and other clinicians (e.g., the Better Access initiative, described below).

The Pharmaceutical Benefits Scheme (PBS) is an Australian Government program that provides subsidized access to prescription medications for residents. Not all TGA-approved insomnia medications are listed on the PBS. By covering part of the cost of approved medicines, the PBS supports Australians to access medications in an affordable and equitable way. Medications included on the PBS are assessed for clinical effectiveness and cost-effectiveness, ensuring that patients receive medications that are both safe and reasonably priced.

# 3. Differential diagnoses

Having difficulty sleeping as well as the daytime consequences outlined in criteria A and B of Table 2 does not always satisfy the criteria for insomnia disorder. Particular attention needs to be given to criteria C-F as many people with disturbed and/or inadequate sleep due to their role as caregivers, shift workers, etc., and those not creating enough time for sleep most likely do not have insomnia disorder. Careful assessment should be conducted in people with insomnia symptoms and other mental/physical health conditions to confirm diagnostic criteria for comorbid insomnia disorder. It is also important to be aware of conditions such as Delayed Sleep-Wake Phase Disorder (See Table S1), which can be mistaken for insomnia. People with these other conditions or circumstances could develop comorbid insomnia disorder and require tailored assessment, advice, education and management regarding their disturbed or lack of sleep.

- **Caregivers**: Consider referral for social supports, as well as tailored problem-solving support about how to create more opportunities for sleep, and referral for sleep problems in recipients of care if needed.
- **Shift workers**: Shift workers with a sleep disorder may experience substantial delays in accessing a diagnosis and care. This may be especially common if any sleep difficulties are attributed to the shift-work schedule, rather than the presence of a potential underlying sleep disorder. Consider referral to a specialist sleep doctor/institute, and specific shift work guidelines for consumers (e.g., fact sheets from the Sleep Health Foundation website).
- **Medical conditions including mental health conditions**: Ensure that comorbid conditions (e.g. depression, anxiety, pain, etc.) are also addressed and treated. Insomnia and these comorbid conditions tend to share a bi-directional relationship where treatment of either disorder will result in improvement of the other (see Comorbid insomnia sections).
- **Inadequate opportunities for sleep**: Consider providing advice, support and/or referral for managing unhelpful and/or unhealthy work or study patterns, bedtime procrastination, potential substance misuse or addiction to TV/gaming/technology.
- **Other sleep disorders**: Several sleep disorders share similar presentations to insomnia (Table S1). For example, Delayed Sleep-Wake Phase Disorder often presents as severe difficulties initiating sleep and daytime impairment^17^. Alternatively, obstructive sleep apnea (OSA) can present as general dissatisfaction with sleep quality, non-restorative sleep, and impaired daytime feeling and functioning^18^. Careful consideration of differential diagnoses may be warranted and appropriate in some cases, however, insomnia can also occur comorbid with other sleep disorders (e.g., comorbid insomnia and sleep apnea). If a comorbid sleep disorder is suspected, referral for a sleep study or referral to a specialist sleep clinician/institute may be considered (See the Assessment section for more detail of assessment tools for other sleep disorders).

##### Table S1. Comorbid sleep disorders and differential diagnoses often associated with insomnia.

| **Disorder** | | **Description and association with insomnia** |
| --- | --- | --- |
| **Sleep-related breathing disorders** | | |
|  | Obstructive sleep apnea | Characterized by repeated episodes of upper airway narrowing or collapse during sleep. Associated with increased risks of chronic mental and physical health conditions (e.g., depression, cardiovascular disease), and can cause daytime feeling and functioning impairment resulting in increased risks of motor-vehicle and workplace accidents. Approximately 30-40% of people with insomnia have comorbid sleep apnea (COMISA)^19^. See specific section on COMISA below. |
| **Movement-related sleep disorder** | | |
|  | Restless Legs Syndrome | Characterized by self-reported strong urge to move legs, typically in the evenings and during periods of rest. Often described as sensation of “restlessness”, “leg discomfort”, “twitchy sensation”, and that “legs want to move on their own”. Often co-occurs with difficulties initiating and/or maintaining sleep, and may be a differential diagnosis of insomnia *or* comorbid with insomnia. |
|  | Periodic limb movement disorder | Frequent limb movements during sleep (especially in lower extremities) that cause sleep disturbance and/or impaired daytime function, mental or physical health. High overlap of periodic limb movements and restless legs syndrome. Often co-occurs with difficulties initiating and/or maintaining sleep, and may be a differential diagnosis of insomnia *or* comorbid with insomnia. |
| **Circadian rhythm sleep-wake disorders** | | |
|  | Delayed Sleep-Wake Phase Disorder | Sleep-wake time that is delayed by approximately 2 hours or more, compared to conventional or societal norms of individual. Overrepresented in adolescents and young adults. May be mis-diagnosed as insomnia with predominant sleep onset difficulties. Differentiate from insomnia by assessing whether sleep onset difficulties persist when patient is able to sleep at desired times (i.e., if ‘insomnia’ symptoms are not present when going to bed at a later time in the evening/early morning). |
|  | Advanced Sleep Wake Phase Disorder | Sleep-wake time that is advanced by approximately 2 hours or more, compared to conventional or societal norms of individual. May present as insomnia with predominant early morning awakening and difficulties returning to sleep, accompanied by early afternoon/evening sleepiness (e.g., falling asleep in front of the TV before bed). Consider differential diagnosis, or comorbidity with insomnia disorder. |
|  | Shift work sleep disorder | Disruption of circadian rhythm due to working long or irregular hours. May present as general sleep complaint, reduced alertness, insomnia or excessive sleepiness in context of shift work. Sleep disorders can co-occur with shift work and should be screened with standardized instruments, rather than attributing all sleep symptoms to shift work context/circumstances. |
| This is not an exhaustive list of sleep disorders. A selection of sleep disorders that share similar presenting features and/or a high comorbid prevalence rate with insomnia are listed. For a full list of clinical sleep disorders, see the International Classification of Sleep Disorders schema^20^.  Insomnia symptoms may initially result from the presence of another un-treated sleep disorder, but develop functional dependence and become a self-maintaining comorbid disorder over time (See Aetiology section). | | |

## 3.1. Comorbid insomnia

### 3.1.1. Comorbid mental health conditions

Approximately 50% of people with insomnia report depression symptoms, and up to 90% of people with major depression report sleep difficulties^21,22^. Insomnia and mental health symptoms are bi-directionally related^21,23^. There is strong evidence that insomnia is a risk factor for incident depression, anxiety, alcohol abuse, and psychosis^24,25^, and that treatment of insomnia improves symptoms of both insomnia and depression in people with both conditions^26,27^. There is emerging evidence that non-pharmacological treatment of insomnia may reduce rates of incident depression^28^. That is, early identification and treatment of insomnia in people without depression may reduce a person’s risk of developing depression.

Insomnia also frequently co-occurs with other mental health conditions including anxiety, bipolar disorder, post-traumatic stress disorder, and schizophrenia, among others^29^. There is a large amount of literature suggesting that bi-directional relationships between insomnia and several comorbid mental health disorders can develop, and there is often benefit in treating insomnia symptoms directly^27,29^. In some cases (e.g., bipolar disorder, schizophrenia), adapted cognitive behavioral therapy for insomnia (CBTi) interventions may be appropriate (especially tailored delivery of sleep restriction therapy that may exacerbate symptoms of some psychiatric conditions)^30,31^. If untreated, insomnia may contribute to relapse of psychiatric conditions, while treatment of insomnia often improves symptoms/management of psychiatric comorbidities^27,29^.

Consequently, when insomnia and mental health symptoms/conditions co-occur, it is important to consider the insomnia as a ‘comorbid’ condition that may warrant specific diagnostic and management attention in addition to the mental health condition, rather than routinely view insomnia as a ‘secondary symptom’ of the mental health condition.

### 3.1.2. Comorbid chronic pain and physical health conditions

Sleep disturbance and insomnia symptoms are associated with several physical health conditions (e.g., acute illness, cardio-metabolic, respiratory, and musculoskeletal conditions, cancer, etc.)^32,33^. Symptoms or management of physical conditions may directly disrupt sleep and precipitate episodes of insomnia, thereby initiating the process of chronic insomnia development. Alternatively, some physical health conditions may continually disturb sleep and maintain symptoms of insomnia over time (e.g., see differential diagnosis, and Table 2 footnotes). Bidirectional relationships between insomnia and physical health conditions may rapidly develop, whereby each condition can exacerbate or impact the other. Clinical trials have also demonstrated that treatment of insomnia in the presence of comorbid physical health conditions is effective, and in some cases can improve symptoms of the comorbidity^34-37^. Therefore, when insomnia and physical health conditions co-occur, it is important to consider a person-centred approach to the management of sleep and physical health symptoms, including targeted assessment and management approaches for the insomnia where appropriate.

### 3.1.3. Comorbid sleep disorders

Insomnia symptoms often co-occur with other sleep disorders (see Table S1). In many cases, insomnia may occur as a comorbid condition with other sleep disorders, and management of both the insomnia and the comorbid sleep disorder should be considered. In other cases, a differential diagnosis may be more suitable if the insomnia symptoms are a direct manifestation of an underlying sleep disorder. It can be difficult to distinguish between patterns of ‘comorbid insomnia’ versus insomnia symptoms occurring as a direct symptom in the presence of other sleep disorders, according to assessment of baseline symptoms alone. To distinguish between these patterns of insomnia (and the need for specific treatment of insomnia) it may be helpful to assess for the presence of insomnia-specific perpetuating factors, or a state of conditioned insomnia. In many cases, insomnia symptoms that have persisted for more than 1-3 months may have developed partial functional independence of the other sleep disorder, and psycho-behavioral perpetuating factors may have formed that partially reinforce the insomnia (e.g., spending excessive time in bed awake, resulting in a state of ‘conditioned insomnia’). Cognitive Behavioral Therapy for insomnia (CBTi) has been reported to improve insomnia symptoms in the presence of comorbid treated/untreated obstructive sleep apnea^38^, and restless legs syndrome^39^.

Population-based studies indicate high rates of undiagnosed sleep disorders in Australian adults^40,41^. Assessing for other sleep disorders such as sleep apnea and restless legs syndrome when managing patients with insomnia may be an important opportunity to identify other previously undiagnosed sleep disorders (see Assessment section).

# 4. Cognitive Behavioral Therapy for insomnia (CBTi)

## 4.1. CBTi for acute insomnia

Approximately 30-50% of adults experience acute insomnia (< 3 months) at any given time, of whom approximately 2-20% develop chronic insomnia disorder^42-44^. Acute insomnia typically occurs in response to precipitating factors (psychological or physical stressors or environmental conditions)^43,45^, and may warrant clinical intervention^20,46^. In many cases, reassurance that sleep patterns will return to normal after the precipitating factor has subsided may be all that is required. It could be suggested that CBTi is less appropriate for acute insomnia if the underlying perpetuating factors that maintain chronic insomnia (and are a target of CBTi) have not yet fully developed. Indeed, in clinical practice, pharmacotherapy is the most frequently prescribed treatment in the case of acute insomnia^47^. However, the 2-20% rate of transition from acute to chronic insomnia, and risks associated with pharmacological management (side effects and dependence)^48^ suggest a role for CBTi in acute presentations.

CBTi may play an important role in preventing the transition from acute to chronic insomnia:

- Perpetuating factors for insomnia may develop before the (arbitrary) 3-month threshold used to define ‘chronic insomnia’. Therefore, CBTi may be indicated before a patient experiences a transition to ‘chronic insomnia disorder’.
- By addressing unhelpful behaviors and cognitions before they become firmly established, the early use of CBTi may prevent the entrenchment of unhelpful cognitions and behaviors known to perpetuate insomnia.
- Access to CBTi may prevent the need for sleep medications, thereby avoiding potential for patterns of dependence and medication use which may further perpetuate the long-term insomnia condition.
- In people with acute insomnia, brief CBTi interventions are associated with moderate improvements in insomnia severity, and reduced rates of transition to ‘chronic insomnia’^49,50^.

Pharmacotherapy also plays a role in the management of acute insomnia. Clinical judgement suggests that short-term pharmacotherapy may be appropriate where there is an obvious underlying precipitating stressor (e.g., bereavement, acute pain, work/study stress), combined with a high level of distress or functional impairment requiring rapid relief, and benefits of treatment with sleep medications outweigh the potential harms^51^. In cases where unhelpful sleep related cognitive and behavioral perpetuating factors have developed, CBTi should be considered alone or in combination with pharmacotherapy^52^.

## 4.2. CBTi effects in comorbid insomnia

CBTi has been extensively studied across various **physical health conditions**, including chronic pain, cancer, cardiovascular disease, gastrointestinal disorders, and neurological disorders^34^. Most interventions employ standard CBTi protocols with minor modifications or additions to address condition-specific factors. Modifications commonly include psychoeducation on perpetuating factors related to the health condition (e.g., pain behaviors^53^, gastrointestinal-related thoughts^54^, or napping during haemodialysis^55^). Some studies also add symptom-specific strategies like activity pacing for chronic pain and post-stroke fatigue^53,56^). In some cases, modifications are made to bedtime restriction therapy, such as allowing post-chemotherapy naps^57^ or creating a higher time in bed limit of 6 hours to mitigate seizure risks in epilepsy^58^. Meta-analytic evidence confirms that CBTi leads to large and clinically meaningful improvements to insomnia, with outcomes comparable to non-comorbid samples^34^.

CBTi has demonstrated effectiveness in treating insomnia among individuals with comorbid mental health conditions, including depression and anxiety disorders, alcohol dependency, bipolar disorder and psychosis/schizophrenia^27^. In these populations, insomnia not only contributes to poor sleep but can also exacerbate psychiatric symptoms, making it a key target for intervention^23,24^. CBTi significantly reduces insomnia symptoms and comorbid psychiatric symptoms, enhancing overall mental health outcomes^27^. However, adaptations may be necessary to account for specific psychiatric challenges in some populations. For example, patients with bipolar disorder require careful monitoring to avoid triggering manic episodes, and benefit from interpersonal, social and chronobiological adaptations^30^, while individuals with schizophrenia benefit from a more personalized approach that addresses cognitive impairments and delusional thinking^59^. In patients with PTSD, CBTi has been found to yield durable improvements in sleep and overall psychosocial functioning up to six months post-treatment^60^. Additionally, therapies such as Imagery Rescripting and Imaginal Exposure have shown promise in reducing nightmare symptoms in this population^61^. This suggests that comprehensive treatment for PTSD should include behavioral sleep interventions. Overall, the evidence demonstrates that CBTi is an adaptable and effective intervention for improving both sleep and psychiatric outcomes in individuals with complex mental health conditions.

Insomnia also frequently co-occurs in the presence of comorbid sleep disorders (e.g., OSA^62^, restless legs syndrome^63^, circadian rhythm sleep-wake disorders^64^ [see *Differential diagnoses* section]). Although insomnia symptoms may initially be precipitated by other sleep disorders, psycho-behavioral perpetuating factors and a state of conditioned insomnia can rapidly develop and maintain the insomnia condition over time (See *Aetiology* section). In this way, insomnia can become a functionally independent comorbid disorder that shares bi-directional relationships, and can impact the course and treatment-response of the other sleep disorder. With adaptations for specific features of comorbid conditions (and concurrent treatments), CBTi is an effective treatment for insomnia in the presence of other sleep disorders including treated and untreated OSA^38^ (see *Comorbid insomnia and sleep apnea* section), restless legs syndrome^39^, and circadian rhythm sleep-wake disorders^17^. Evans and colleagues provide a comprehensive review of adapting CBTi for patients with comorbid circadian rhythm disruption^17^. Psychologists with expertise in insomnia management often aim to treat the circadian disruption first in the management of clients with comorbid insomnia and circadian rhythm disorders.

## 4.3. CBTi in patients using sleep medications

Whilst there are no representative national data on the prevalence of sleep medication use (i.e., benzodiazepines/z-drugs) during the course of CBTi, sleep medicines are the mainstay of treatment for insomnia in Australian primary care, despite guidelines consistently recommending them second-line for a minority of patients^7^. Consequently, it stands to reason that a large number of patients treated with CBTi will be using these medicines on an as-needed or regular basis. This can be problematic as up to 80% of individuals taking benzodiazepines or ‘z-drugs’ for longer than six months will experience withdrawal symptoms if they stop^65,66^. The most common symptoms of withdrawal are insomnia and anxiety, but also nightmares, flu-like symptoms, sensory sensitivities, tremor, memory difficulties, and nausea^47^. Accordingly, any attempt to reduce or stop sleep medications that have been used long-term needs careful consideration. This should include screening for dependence (both physiological and psychological), education on safe tapering and strategies for withdrawal management. The most successful taper plan is likely to be one that is patient-guided and uses a collaborative approach^67^.

Notwithstanding, CBTi is an effective insomnia treatment in adults using sleep medicines^68,69^. Several studies have specifically examined the effect of CBTi on facilitating patients to reduce or cease sleep medication use. A narrative review reported that CBTi is associated with a 30-40% rate of sleep medication cessation in people with insomnia^69^. A systematic review and meta-analysis of 8 randomized controlled trials (RCTs) found that CBTi combined with gradual tapering was associated with significantly greater rates of sleep medication cessation at short-term, but not long-term follow-up, compared to tapering alone^70^. Returning to sleep medication use is not unusual in follow up studies. A recent RCT revealed that while combining CBTi with a supervised taper of sleep medication was effective, patients reported a 2-fold increase in the use of alternative sleep medicines or substances at 3-month follow-up, suggesting the reliance on a sleeping aid did not resolve, it simply shifted^71^. Given long-term use of benzodiazepines and ‘z-drugs’ is common, it would be expected that many individuals could take months or even years to successfully stop their sleep medicine. Booster CBTi sessions may then be required to maintain the short-term improvements^72^ following CBTi. Possible mechanisms by which CBTi facilitates cessation of sleep medication include; reducing rebound insomnia symptoms experienced during sleep medication withdrawal, providing patients with the confidence that their sleep can improve without medicines, providing evidence-based information on the factors that control the timing and quality of sleep independently of medicines, or treating the psycho-behavioral causes of insomnia and replacing the need for medicines. See Simpson and Manber for a detailed review of the integration of CBTi and sleep medication taper interventions^67^.

## 4.4. CBTi risks and side-effects

Clinical trials of pharmacotherapy interventions for insomnia measure side-effects and adverse events associated with medications reasonably consistently. However, comparatively few clinical trials of CBTi have investigated side-effects or risks of adverse events in a systematic way.

CBTi may be associated with a short-term increase in daytime sleepiness and corresponding reduction in alertness. Specifically, this increase in sleepiness is likely associated with the first 1-3 weeks of bedtime restriction therapy and stimulus control therapy (see Table 5), when time in bed is reduced to its lowest point, and the patient is instructed to get out of bed if not asleep, but the conditioned insomnia response has not been ameliorated. This can result in a temporary reduction in sleep duration, before sleep pressure gradually increases and starts to reduce sleep onset latency and nocturnal wake time. Kyle and colleagues used a mixed-methods approach to investigate side-effects of bedtime restriction therapy in 18 patients with insomnia, and observed high levels of fatigue/exhaustion, extreme sleepiness, reduced motivation/energy, and headache/migraine^73^. In a related quantitative study, Kyle and colleagues^74^ also observed that bedtime restriction therapy is associated with a short term reduction in sleep duration, and corresponding increase in daytime sleepiness and reduced objective vigilance. Sweetman and colleagues^75^ similarly reported a short-term and small increase in self-report symptoms of daytime sleepiness during the first week of bedtime restriction therapy in patients with comorbid insomnia and sleep apnea. Turner and colleagues^76^ reported that CBTi is associated with impairment of some neurocognitive performance tests at post-treatment in patients with comorbid insomnia and sleep apnea, raising the need for careful delivery of CBTi in this group. Future clinical trials of CBTi should aim to systematically document treatment side-effects and adverse events, and temporal changes in the prevalence and severity of these side-effects as bedtime restriction therapy is initiated, maintained, and gradually increased. It is important to warn patients beginning CBTi (and specifically bedtime restriction therapy) of the potential for these short-term side effects, and strategies to mitigate risks associated with sleepiness or alertness failure. Measures of additional side-effects and adverse events should be collected in future clinical trials of CBTi. Patients should be pre-warned of potential side-effects, and care should be taken to reinforce the likely long-term benefits of CBTi despite these short-term side-effects.

## 4.5. Stepped care models for insomnia

Stepped-care models for insomnia are being actively researched in Australia^77-80^. Theoretically, stepped-care models for insomnia represent a treatment approach that include multiple evidence-based CBTi modalities into a single model of care, which maximises the efficient use of available CBTi resources/modalities to support a greater number of patients to access CBTi in a cost-effective way. Different interventions are generally ordered based on their level of ascending cost and resource requirements, often with interventions involving a greater degree of clinician time placed at higher ‘levels’. For example, stepped-care models may begin with less resource intensive and more accessible self-guided digital CBTi options, before progressing to more intensive group-format, or guided-digital CBTi options, before progressing to individualized treatment with trained/experienced/specialist sleep clinicians. Stepped-care models may be designed for all patients to commence treatment at lower levels and ‘step-up’ if they do not respond, or a hybrid stepped-care approach may be used to screen patients at baseline and allocate them to different starting ‘levels’ of the CBTi based on their preferences, insomnia severity, characteristics, work/study/lifestyle factors, comorbidities, and other parameters^81,82^.

Theoretically, stepped-care models may be more cost-effective than current treatment models if most patients are effectively managed at lower levels. There is also potential to reduce burden on specialist resources, thereby allowing specialists to support patients with more complex needs. For example, in a recent RCT, Sweetman and colleagues found that approximately 80% of Australian participants screened for a clinical trial of self-guided digital CBTi were appropriate for self-guided management, while 20% presented with symptoms that were more appropriate for specialist care^80^. Of the 80% that were recruited to the study, most participants were effectively treated with digital CBTi, and only a minority of participants did not respond to digital CBTi, and were also provided recommendations for further care at higher-intensity levels^80^. Although attractive from an economic and scalability perspective, stepped-care models may also increase burden on clinicians and patients. Specialist sleep clinicians may manage an increasingly higher proportion of patients with ‘complex’ insomnia that progress to higher levels (assuming that patients with less complex needs are appropriately triaged and managed at lower levels). Furthermore, stepped-care models may also create burden for patients – especially those that progress through multiple ‘steps’ without experiencing improvement if the initial triaging approach did not allocate them to an appropriate starting ‘level’. Patients may also experience difficulty navigating a complex system with multiple stages of entry, repeated assessment of symptoms after each intervention, and engaging with different levels of a seemingly similar intervention (E.g., “Why do I need to try sleep restriction yet again?”). Stepped-care models of CBTi represent an exciting opportunity to maximise the reach and cost-effectiveness of CBTi, and to efficiently treat a large number of patients. However, mixed-methods effectiveness and implementation research in Australia is needed to progress this area and ensure that models of care best meet the needs of consumers, clinicians, and the health system.

## 4.6. Other psychological and behavioral approaches

### 4.6.1. Healthy sleep habits (Sleep hygiene)

Information about healthy sleep habits^83^ (also called “sleep hygiene”, or “healthy sleep practices”) refer to generic recommendations about healthy sleep behaviors, routines, or environmental factors. For example, avoiding caffeine after 2pm, limiting mobile phone use in bed, avoiding large meals or strenuous exercise immediately before bed, and maintaining a bedroom environment that is cool, dark, quiet and comfortable. This information is often freely available online and in print material and is frequently provided as a treatment to people with chronic insomnia.

Information about healthy sleep practices is different from CBTi. Although ‘sleep hygiene’ information is included in many CBTi programs, it is not a sufficient stand-alone treatment for insomnia^4^. In fact, many people with chronic insomnia presenting for CBTi report that they have already accessed and used sleep hygiene recommendations to limited benefit^80^. Many clinicians may believe that CBTi and ‘sleep hygiene’ are the same thing^11^, and consequently form a belief that CBTi is not effective if they have provided patients with sleep hygiene recommendations that have not alleviated insomnia symptoms. It is therefore important to provide education to clinicians to understand the differences between ‘sleep hygiene’ material and multi-component CBTi.

Although not an effective stand-alone treatment for chronic insomnia, information about healthy sleep practices may be a useful preventive health intervention^84,85^. For example, maintaining healthy sleep practices may prevent poor sleep habits from forming, or the likelihood of experiencing a transition from acute to chronic insomnia. A recent Australian policy evidence review led by the Mitchell Institute^85^ describes the many ways that public sleep health is strongly and bi-directionally related to many other areas of physical and mental wellbeing, and the importance of promoting preventive sleep health practices.

### 4.6.2. Mindfulness based therapy for insomnia

Mindfulness-based therapy for insomnia refers to a suite of interventions that integrate sleep medicine principles, behavior therapies for insomnia, and meditation practices to improve insomnia. These therapies use metacognitive processes (thinking about thinking) to increase awareness and acceptance of mental and physical states associated with chronic insomnia. Patients work to recognise emotional reactions to sleep disturbance and threats to sleep, and then accept these reactions. The result is reduced struggle with insomnia which facilitates down-regulation of arousal and improved sleep^86^. Mindfulness-based therapy for insomnia may be delivered in combination with ‘traditional’ behavioral components of CBTi such as stimulus control therapy and bedtime restriction therapy for insomnia, and may be delivered in an individual or group-therapy format^87,88^.

Initial pilot studies indicated promising effects of mindfulness-based therapies on reducing wake time and improving global insomnia severity^89^. Several meta-analyses of mindfulness-based interventions for insomnia have subsequently reported promising results^90-92^.

A head-to-head comparison of mindfulness-based stress reduction strategies versus CBTi in 327 patients with comorbid insomnia and cancer found that CBTi was associated with greater post-treatment improvements in insomnia symptoms^93^.

Mindfulness-based therapies for insomnia may represent an appropriate alternative non-pharmacological insomnia treatment where CBTi is difficult to access, or a second-line treatment for insomnia if CBTi is not effective^88,94^. This would also provide an additional non-pharmacological alternative to CBTi before commencing sleep medication. Mindfulness-based therapies for insomnia may be particularly beneficial for patients who experience persistent insomnia symptoms associated with elevated cognitive arousal following CBTi^88^. Although CBTi is a very effective treatment, bedtime restriction therapy can also be difficult, or potentially unsafe in some situation/occupations. Therefore, commencing with mindfulness-based interventions may be beneficial before introducing the behavioral components of CBTi in some patients. Further research is needed to guide which patients with insomnia are more suitable initially for CBTi, mindfulness-based therapies, or other treatment approaches. An Australian digital intervention combining mindfulness and CBTi content is also available^95^.

### 4.6.3. Intensive sleep retraining

Intensive sleep retraining is a behavioral treatment for sleep onset insomnia symptoms developed in Australia^96^, in which a patient experiences repeated opportunities to fall asleep for a short period, before being woken, over a 24-hour period^97^. The experience of progressively more rapid sleep onset times in the presence of increasing homeostatic sleep pressure and circadian drive to sleep, and provision of feedback about sleep onset following each opportunity aims to gradually re-associate the bed with rapid sleep onset and reduce any conditioned association between bed and wake^98^. It follows the same principles as the well-established behavioral therapy of Stimulus Control Therapy^99^ except it produces the same outcome after 24 hours instead of several weeks^100^. Intensive sleep retraining was initially developed as a sleep laboratory intervention including a sleep onset opportunity every 30 minutes throughout a 24-hour period. Polysomnography was used to record sleep/wake patterns in real-time and precisely identify sleep onset during each opportunity. If a patient fell asleep, they were woken after approximately 3 minutes, provided feedback on their sleep onset latency, and remained awake until the subsequent trial.

Initial studies reported promising effects of intensive sleep retraining on sleep onset latency and sleep duration when administered in a laboratory setting^97,98^. Due to high costs and increasing demand for in-laboratory studies, intensive sleep retraining has also been translated to self-guided digital interventions^101^. A recent systematic review and meta-analysis including the three trials above reported significant post-treatment improvements in diary-measured sleep onset latency, sleep duration and sleep efficiency, noting that larger controlled trials are required to definitively determine the effectiveness of the intervention^102^. Such a trial is already underway with results expected in 2026^103^.

### 4.6.4. Acceptance Commitment Therapy

Acceptance Commitment Therapy (ACT) has emerged as a promising treatment for insomnia, addressing both the psychological and behavioral aspects of the disorder. Two systematic reviews of ACT support its efficacy in treating insomnia (and insomnia symptoms), both as a standalone intervention and in combination with a brief CBTi intervention^104,105^. ACT focuses on fostering psychological flexibility through interventions designed to increase acceptance, mindfulness, and values-driven action. This results in reduced avoidance of insomnia-related thoughts and emotions and an increase in engagement with meaningful daytime activities that are consistent with the patient’s life values. The resulting reduction in counterproductive sleep effort fosters a healthier relationship with sleep. ACTs focus on reducing experiential avoidance (the tendency to suppress or avoid distressing thoughts or feelings) is particularly relevant for individuals who experience a cycle of worry and frustration about their sleep.

As a standalone therapy, ACT improves sleep quality, and quality of life, and reduces insomnia severity, depression and anxiety symptoms. One small study supported the effectiveness of ACT in participants who had not responded to CBTi^106^. An RCT evaluating digital ACT (N=86) found a medium effect size for self-reported sleep compared to waitlist^107^. ACT has been associated with a slower treatment response than CBTi, but with improvements that grow and sustain over the follow-up period^108^. There is emerging evidence that, as a standalone therapy, ACT may be less effective than CBTi^109^ but equal in effectiveness when combined with behavioral interventions^110^. One small RCT found that ACT was more effective than CBTi in reducing anxiety^111^. When combined with brief CBTi, ACT may facilitate adherence to sleep restriction and stimulus control by increasing tolerance of short-term discomfort^104^. ACT is a promising treatment in patients with low adherence to CBTi, or as an adjunct to CBTi.

# 5. Pharmacological management of insomnia

Medications commonly used for insomnia are described below, and in Table 7 of the manuscript. This includes medications that are accessible over the counter and via a prescription, and medicines with and without an indication for insomnia.

Specific sleep medication for insomnia are associated with rapid small-to-moderate effects on subjective/objective outcomes (see below). However, small-to-moderate placebo effects also occur. For example, a 2015 meta-analysis of RCTs investigating the effect of sleep medications on objective sleep parameters reported that approximately 64% of the therapeutic response to sleep medications is observed in placebo-control groups^112^.

In general, it is recommended that clinicians weigh the potential for dependence, side-effects, and adverse events associated with the below medications, against health risks of not treating insomnia. If used, medications should be prescribed at the lowest effective dose for the shortest possible duration (see Table 7 of manuscript). Support to discontinue sleep medication should be provided from the outset, including information about dependence risks, a structured withdrawal plan, and concurrent CBTi to facilitate withdrawal. Previous clinical practice guidelines have reviewed evidence regarding the effectiveness and safety of specific medicines commonly used for insomnia (including a European Insomnia Guideline^2^, and a American Academy of Sleep Medicine (AASM) Clinical Practice Guideline^113^). Meta-analytic evidence of the effectiveness and safety of medicines available in Australia is described below.

## 5.1. Benzodiazepines

Benzodiazepines are central nervous system depressants that enhance the effect of the brain's main inhibitory neurotransmitter, gamma-aminobutyric acid^114^. By reducing neuronal activity, benzodiazepines produce a calming effect on the brain and body which can include a sedative-hypnotic effect. They are often prescribed in the management of insomnia, anxiety, and seizures^114^. The most common benzodiazepines prescribed for insomnia in Australia are temazepam, oxazepam, nitrazepam, and diazepam (see Table 7 of manuscript).

A 2000 meta-analysis of RCTs reported a 4.2-minute improvement in subjective sleep latency, and a 61.8-minute increase in overall sleep duration^115^. A 2007 systematic review of benzodiazepines for individuals with chronic insomnia similarly reported significant reductions in sleep onset latency, both through objective measures (polysomnography) by 10 minutes and subjective self-report by 19.6 minutes^116^. A 2023 systematic review^117^ investigated the effect of benzodiazepines on sleep architecture (proportion of time in different ‘sleep stages’), and found that improvements in sleep duration are largely due to increased stage 2 (moderate) sleep, and that benzodiazepines are in fact associated with reduced stage 3-4 (deep) and Rapid Eye Movement sleep.

Benzodiazepines are associated with increased risks of adverse events including somnolence, dizziness, nausea, fatigue, but also increased risk of road accidents and falls in older adults^116,118^. Benzodiazepines have also been shown to impact cognitive abilities. One meta-analysis^119^ found that long-term benzodiazepine users (>1 year) performed worse in cognitive domains compared to controls, such as but not limited to sensory processing, psychomotor speed, nonverbal memory, and visuospatial ability. A systematic review also found both amnestic and non-amnestic cognitive impacts, with a dose-response relationship^120^.

Most clinical guidelines recommend that benzodiazepine prescriptions for insomnia should be limited to short-term use (i.e., less than 4 weeks; RACGP^47^, RANZCP^121^, AMH^114^, NPS^122^, TG^123^). While data demonstrating the clinical effects of benzodiazepines for insomnia is not available from long-term trials, the evidence of the risks is clear^47,124^. Long-term use of benzodiazepines carries a risk of dependence, possible tolerance even after a couple of weeks, and abrupt cessation increases the risk of withdrawal and more acute symptoms, such as seizures^65,125^. Short-acting benzodiazepines are reported to be associated with a significantly greater risk of withdrawal than long-acting benzodiazepines^126^. Recommendations from an expert consensus group^127^ were that patients experiencing insomnia gradually taper from benzodiazepines at a rate of 10-25% per week while receiving concurrent CBTi. However, the authors noted that a more gradual tapering process could be considered, based on the characteristics of the patient and presence of withdrawal symptoms. More recently, the Maudsley Deprescribing Guidelines outlined a range of risk factors to identify the appropriate rate of reduction (for benzodiazepines and ‘z-drugs’), ranging from 5-20% every 1-4 weeks based on factors such as age, previous withdrawal episodes, and duration of use^128^.

## 5.2. Z-drugs

‘Z-drugs’ are a group of non-benzodiazepine hypnotic medicines which (like benzodiazepines) act on the gamma-aminobutyric acid receptor but do so with greater specificity to subunits linked to sedation. Zolpidem and zopiclone are both TGA approved in Australia (see Table 7 of manuscript).

A 2012 meta-analysis of controlled trials of ‘z-drugs’ in the treatment of insomnia found a significant 22-minute improvement in sleep onset latency as measured by polysomnography and a 7-minute improvement in subjective sleep latency^129^. A 2021 meta-analysis of Zolpidem reported significantly greater improvements than placebo in sleep duration and sleep onset latency, but no difference in wake after sleep onset at 1 month follow-up^130^. Subgroup analyses indicated that these results occurred only in the ‘elderly’ sub-group (defined as age >65 years). A 2023 network meta-analysis reported that ‘z-drug’s improved sleep onset by 12.1 minutes on objective measures (polysomnography) and by 10.1 minutes on subjective measures, increased total sleep time by 22.3 minutes on objective measures, and reduced nighttime wakings by 16.7 minutes on subjective self-reporting^131^.

When comparing the efficacy of individual ‘z-drugs’, zolpidem is reported to have a significantly higher rate of improvement in sleep latency than zopiclone^132,133^, perhaps due to its quicker onset, whereas zopiclone improves total sleep time and reduces wakings^134,135^.

However, ‘z-drugs’ are associated with adverse effects including dizziness, nervousness, falls, anxiety, memory impairments, hallucinations, confusion, and fatigue^136^. Exposure to ‘z-drugs’ is reportedly associated with more than 60% increase in the risk of fractures, and exposure to zolpidem is associated with a doubling of the odds of injury^137^. Zopiclone was associated with negatively impacted verbal memory on the next day, and zolpidem was associated with negatively impacted attention, verbal memory, and psychomotor speed on the next day^138^. Furthermore, systematic reviews suggest zolpidem has an uncommon but probable association with complex sleep behaviors (e.g., sleepwalking, sleep eating), occurring in around 5% of patients^139,140^.

Several studies show that the rates of dependence observed with ‘z-drugs’ is similar to that seen in benzodiazepines^141-143^, with higher doses and longer duration of use associated with a higher risk of dependency. There has been some suggestion that zolpidem has higher rates of dependence than zopiclone^144^. Similar to benzodiazepines, a slow taper is recommended, with a general rule of reductions of no more than 5-20% of the dose per week tailored to each patient (see benzodiazepine section above)^127^.

## 5.3. Melatonin

Melatonin is a naturally occurring hormone synthesized in the pineal gland, that is associated with light-dark exposure (e.g., day-night cycles), levels of sleepiness and the internal circadian rhythm. Melatonin production is generally reduced with bright light exposure, and increased at night. Melatonin is potentially the most common medication used to manage insomnia in Australia^145^. While it is only available on prescription (Schedule 4) or as a Pharmacist only (Schedule 3) medicine in Australia, it is an unregulated product overseas and people report purchasing melatonin online^146^, resulting in concern that many people will self-manage their insomnia with melatonin without appropriate oversight by a health professional^147,148^.

A 2017 AASM clinical practice guideline^113^ recommended against the use of melatonin for the management of insomnia in adults, based on limited evidence of effectiveness at the time. A 2022 meta-analysis by Choi and colleagues^149^ of 24 RCTs reported no effect of melatonin on sleep onset latency, sleep duration, or sleep efficiency in adults. However, a more recent 2023 meta-analysis by Maruani and colleagues^150^ reporting on 15 randomized placebo-controlled studies investigating the effect of melatonin in people with insomnia disorder found that melatonin resulted in improved short-term sleep onset latency measured *subjectively* (4.7 minutes), and *objectively* (4.8 minutes), but not other sleep-wake parameters or frequency of awakenings. Subgroup analyses additionally indicated significant effects of prolonged release melatonin on objective sleep efficiency (percentage of time in bed spent asleep) in those aged ≥55 years (3% difference, versus placebo). A 2023 European Insomnia Guideline^2^ acknowledged the small short-term improvements identified in some meta-analyses, but conflicting results across the literature. It was recommended that immediate-release melatonin is not appropriate for the management of insomnia (in the absence of circadian rhythm disruption), however, prolonged release melatonin may be used for up to 3 months for insomnia management in people aged ≥55 with consideration of advantages and disadvantages. This recommendation aligns with the availability of melatonin as a Schedule 3 (Pharmacist only) medicine to people aged ≥55 years in Australia (see Table 7 of manuscript)^151^.

Insomnia symptoms may co-occur with circadian rhythm disturbance. For example, difficulties initiating sleep may occur in the context of a delayed circadian rhythm, and difficulties with early morning awakenings may occur in the context of an advanced circadian rhythm. The effect of melatonin on reducing sleep onset latency may be arising from the inclusion of many people in clinical trials who have mainly sleep onset insomnia and potentially some circadian rhythm phase delay, and when instructed to take 2 mg melatonin at the desired bed time it reduces sleep onset latency because melatonin does have a sedating effect if administered before endogenous dim light melatonin onset^152^. The use of melatonin for insomnia symptoms in the context of circadian rhythm disorders, jet lag, and shift work may be more appropriate than for the management of insomnia in the absence of circadian rhythm disturbance^153^.

Risks of dependence, side effects and adverse events are lower for melatonin than benzodiazepines and ‘z-drugs’^154^. However, effect sizes on insomnia symptoms are also comparatively smaller, resulting in a compromised risk-benefit ratio^154^. Side effects including drowsiness, daytime sleepiness, headaches and nausea are reported by the TGA at higher doses of Melatonin^155^. The TGA in September 2025 issued a statement on safety concerns regarding imported unregistered melatonin products^156^. Internationally purchased melatonin products have also been found to contain vastly different dosages of melatonin from the advertised amount (-83% to +478% of the labelled content)^147,148^. Consequently, it is important to encourage patients considering melatonin products to purchase TGA-approved or compounded products from an Australian pharmacy. Purchasing from an Australian pharmacy ensures the product falls under Australian legislative and regulatory requirements for quality and safety. Melatonin should be used under direction and prescription from a medical practitioner, and not sourced independently (e.g., online).

## 5.4. Dual orexin receptor antagonists

Dual orexin receptor antagonists selectively bind to orexin receptors 1 and 2, to inhibit the effects of the arousal system and promote sleep onset and maintenance. Suvorexant and lemborexant are TGA approved for management of chronic insomnia in Australia (see Table 7 of manuscript).

A 2017 meta-analysis^157^ of four randomized placebo controlled trials of suvorexant reported a 1.4-point greater improvement in the Insomnia Severity Index in the intervention group at 3-months, and a 16.4 minute increase in subjective sleep duration in the intervention group at 12-months, but no significant difference in subjective sleep onset latency by 12-month follow-up. Suvorexant was associated with increased risks of daytime sleepiness, fatigue, abnormal dreams and excessive daytime sleepiness, compared to placebo. Effect sizes on insomnia severity and side-effect profiles are similar to a 2015 meta-analysis of placebo-controlled trials of suvorexant^158^ and a 2022 meta-analysis of the effects of suvorexant and lemborexant^159^. A 2020 network meta-analysis^160^ of the comparative efficacy of lemborexant versus suvorexant reported the lemborexant was associated with greater short-term improvements in subjective sleep parameters at 1-week, however these differences did not persist by 1-month follow-up. A 2022 systematic review and network meta-analysis of 13 double-blind randomized placebo-controlled trials found that dual orexin receptor antagonists were associated with statistically significant improvement in subjective and objective sleep parameters, but were associated with increased risks of daytime sleepiness, abnormal dreams, fatigue, and dry mouth, among other side effects^161^. A 2023 network meta-analysis^162^ investigating the efficacy and tolerability of 20 medications for insomnia reported that orexin receptor antagonists were more efficacious than ‘z-drugs’, placebo, and melatonin receptor agonists at improving wake after sleep onset and sleep efficiency. Effect sizes for some outcomes are negligible in meta-analyses. For example, lemborexant was associated with a 1.52 point difference (95%CI = 1.92-1.11) in the Insomnia Severity Index, while suvorexant was associated with a 1.70 point difference (2.61-0.79), both below the 6-point minimum clinically important difference threshold^161,163^. Several meta-analyses cited above reported greater risks of daytime sleepiness, fatigue, abnormal dreams, dry mouth, and back pain compared to placebo.

Dual orexin receptor antagonists appear to have a favourable dependence and risk profile compared to benzodiazepines and ‘z-drugs’, and are generally recommended for management of chronic insomnia^114,123^. Compared to placebo, these medications are associated with increased risks of several of the daytime impairments that often motivate patients to initially seek treatment (e.g., daytime sleepiness, fatigue, and headaches)^10^. Statistically significant effects on sleep parameters are observed, however effect sizes from several meta-analyses fall short of accepted thresholds for clinically meaningful improvement in global insomnia severity^161^. Clinicians need to carefully weigh potential benefits versus harms in any decision to prescribe dual orexin receptor antagonists.

## 5.5. Sedating antihistamines

Sedating antihistamines are not recommended for the management of insomnia due to insufficient evidence of benefit and risk of significant adverse effects^123^. Sedating (or ‘first generation’) antihistamines cross the blood-brain barrier and bind to H1 receptors in the central nervous system reducing the effects of histamine, causing sedative and anticholinergic effects^164,165^. Doxylamine, and diphenhydramine are available as Schedule 3 (Pharmacist Only) medicines for the short-term management of sleeplessness (see Table 7 of manuscript).

A 2022 systematic review and meta-analysis found diphenhydramine and doxylamine showed some efficacy in terms of quality of sleep in the short term. However, there was no evidence to suggest their benefit in terms of numbers of dropouts and adverse effects, and there was no data for long-term treatment of insomnia^124^.

A 2016 meta-analysis found overall evidence was weak for the effectiveness of diphenhydramine for improving sleep onset and total sleep time^113^, which informed the AASM Clinical Practice Guideline for the Pharmacologic Treatment of Chronic Insomnia in Adults recommendation against the use of diphenhydramine for the treatment of sleep onset and sleep maintenance insomnia in adults^113^.

Based on a 2022 systematic review reporting no high-quality randomized placebo-controlled trials demonstrating evidence of the effectiveness of sedating antihistamines for insomnia^166^, the 2023 European insomnia guideline does not recommend antihistamines for insomnia management^2^.

Overall, sedating antihistamines are not recommended for the treatment of insomnia. This is due to insufficient evidence they are beneficial, risk of significant adverse effects (including anticholinergic effects, daytime sedation and cognitive and psychomotor impairment) and rapid tolerance to sedating effects^47,123,164,167^. If they are used by adults for the management of insomnia, use should be short term, up to a maximum of 10 consecutive days^164,168^. Common adverse effects include sedation, dizziness, psychomotor impairment (which may persist the next day and in the absence of sedation), confusion and impaired cognition^123,164^. Anticholinergic adverse effects are common (e.g., urinary retention, dry mouth, blurred vision and constipation). Older people and children have increased risk of adverse effects^123,164^. Tolerance can quickly develop to sedative effects, however withdrawal symptoms are usually mild^123,168^.

## 5.6. Antidepressant medications

Antidepressant medications are not recommended for the management of insomnia due to insufficient high-quality evidence of long-term effectiveness and risk of significant adverse effects^123^. Amitriptyline and mirtazapine are the most common antidepressants used (off-label [i.e., not TGA-approved for this indication]) for insomnia management in Australia (see Table 7 of manuscript). Antidepressants (and antipsychotics, see below) may be indicated for comorbid mental health conditions co-existing with insomnia.

Amitriptyline is a tricyclic antidepressant that exerts its therapeutic effect by inhibiting the reuptake of noradrenaline and serotonin^114^. Tricyclic antidepressants also block cholinergic, histaminergic and alpha_1_-adrenergic and serotonergic receptors, which is thought to be unrelated to their therapeutic effect, but contributes to adverse effects such as sedation, dry mouth, blurred vision, constipation, weight gain, orthostatic hypotension, sinus tachycardia, urinary hesitancy or retention, reduced gastrointestinal motility, anticholinergic delirium, sexual adverse effects, tremor, dizziness, sweating, agitation, paradoxical insomnia, anxiety and confusion^114^.

Mirtazapine is a tetracyclic antidepressant that acts by postsynaptic blockade of serotonin (5HT_2_ and 5HT_3_ receptors) and presynaptic blockade of central alpha_2_-adrenergic inhibitory autoreceptors^114^. It is also a potent H_1_ antagonist which accounts for its sedative effects. Common side effects include increased appetite, weight gain, sedation, weakness and peripheral oedema^114^.

A systematic review and meta-analysis by Everitt and colleagues in 2018^169^ identified 23 RCTs comparing antidepressant medication to active or inactive control, for the management of insomnia. Based on the small samples, short-term follow-up and methodological limitations, there was insufficient evidence to recommend that antidepressants are used in the management of insomnia. Consequently, based on limited evidence, antidepressant medications are not recommended for the management of insomnia in Australia.

## 5.7. Antipsychotic medications

Antipsychotic medications are not recommended for the management of insomnia, due to insufficient high-quality evidence of long-term effectiveness and risk of significant adverse effects^123^. Antipsychotics are thought to exert their effect by blocking dopaminergic transmission, with all agents likely blocking D_2_ receptors, and variability in D_1_ and 5HT_2_ blockade influencing different therapeutic and adverse effects. Antipsychotics are associated with potential side effects including sedation, anxiety, agitation, dystonia, akathisia, parkinsonism, orthostatic hypotension, tachycardia and blurred vision^114^.

Quetiapine and olanzapine are the most common antipsychotics used (off-label) for insomnia management in Australia (see Table 7 of manuscript). In addition to the above side effects, olanzapine has been associated with hyperglycaemia and peripheral oedema^114^. Quetiapine is specifically associated with somnolence, hence its off-label use in insomnia^114^. Based on limited available evidence of effectiveness in clinical trials, and risks of side effects, antipsychotic medications are not recommended for the management of insomnia in Australia^123^.

## 5.8. Cannabinoids

Medicinal cannabis is not currently recommended for the management of insomnia in adults and there are no TGA approved products currently available for this indication^123^. Despite this, sleep problems are one of the most common reasons that people in Australia use medicinal cannabis^170^. The cannabis plant contains around 100 cannabinoids and roughly 300 non-cannabinoid chemicals. The two main cannabinoids used for medicinal purposes are delta-9-tetrahydrocannabinol (THC) and cannabidiol (CBD). Medicinal cannabis products are available in various formulations, containing different ratios of THC and CBD, ranging from predominantly THC or CBD to balanced combinations. There are only currently two TGA approved medicinal cannabis products available in Australia, neither of which are indicated for the management of sleep. In Australia, prescribers can access unapproved medicinal cannabis products through the Special Access Scheme or Authorized Prescriber Scheme. Since access to medicinal cannabis started via Special Access Scheme in 2016 in Australia, sleep/insomnia has become the third most common indication for medicinal cannabis prescription^171^.

THC is associated with experiencing a “high”, whilst CBD is non-intoxicating and has more of an anxiolytic effect^172^. Cannabinol (CBN) is an oxidative of THC and is marketed as the “sleepy cannabinoid”. Both THC and CBD are believed to exert influence on sleep-wake patterns through interaction with the endocannabinoid system. In a systematic review of medicinal cannabis and cannabinoids^173^, a small improvement in sleep quality was evident in non-cancer chronic pain individuals and only a very small improvement for chronic cancer patients. Cannabinoids have potential utility in treating insomnia, but these studies are limited by small sample sizes and lack controlled study designs^174^. Among four placebo-controlled cross-over RCTs, only one has reported a statistically significant improvement in insomnia severity^175-177^. Walsh and colleagues^175^ reported no improvement in polysomnography measured sleep parameters, but reduced actigraphy-measured wake after sleep onset and increased sleep duration, and a statistically significant 5.1 point improvement in the Insomnia Severity Index (below the Minimum Clinically Important Difference delta of 6 points^163^) with 2 weeks of combined THC 10mg/CBN 1mg/CBD 0.5mg (option to double dose from the fourth night) compared to placebo. Alternatively, Ried and colleagues^176^ reported no effect of 2 weeks of combined THC 10mg/CBD 15mg on the Insomnia Severity Index compared to placebo, Suraev and colleagues^178^ reported that combined 10mg THC/200mg CBD reduced sleep duration and Rapid Eye Movement sleep measured via polysomnography acutely compared to placebo-control, Narayan and colleagues^177^ reported no effect of 150mg cannabidiol on insomnia severity or sleep parameters after 2 weeks, but improved self-reported wellbeing compared to placebo, and finally, Wang and colleagues^179^ reported that CBD 300mg/terpenes over a 4-week period resulted in an increase in the proportion of slow wave sleep and rapid eye movement sleep, but not significant change in sleep duration.

Studies investigating ‘next day’ impairment following use of oral medicinal cannabis have reported no noticeable deficits in cognitive and psychomotor function (e.g., simulated driving performance, neurocognitive testing, etc.)^180^. This is supported by a recent systematic review reporting limited overall evidence that cannabis use impairs next day performance^181^. More research is needed to investigate the effect of long-term Cannabinoids on other physiological side-effects and interactions with other medications.

More research is required to investigate the specific cannabinoids (including different doses, and maintenance of effects) that are most effective in the management of insomnia. Despite this, the use of cannabis to manage sleep problems is already common practice^170^.

## 5.9. Complementary and alternative medications

Complementary and Alternative Medications (CAM) refer to pharmacological and nonpharmacological products and practices that are external to standard medical care. CAMs represent a rapidly growing area of the Australian healthcare system, and many CAMs are promoted for the management of insomnia disorder. A 2007 study^182^ of 1,067 older adults found that 59% had used a CAM; primarily clinical nutrition, chiropractic, massage therapy, meditation, and herbal medications. A 2019 cross-sectional survey of 2,025 Australian adults found that 68.8% of those with a sleep disorder used complementary and alternative medications, compared to 52% of those without a sleep disorder^183^. The most common CAM practitioners visited by those with a sleep disorder were; massage therapists, chiropractors, acupuncturists, naturopaths, yoga teachers, osteopaths, traditional Chinese medicine practitioners, aromatherapists, homeopaths, and other natural health practitioners. The most common products used by those with a sleep disorder were; Vitamins/mineral supplements, aromatherapy oils, Western or Chinese herbal medicines, and Homeopathy.

Systematic reviews of several CAM therapies indicate potential efficacy of valerian^184^, meditative movement (including yoga, tai chi qi gong)^185^, and acupuncture^186,187^, however they also acknowledge significant methodological issues and potential for publication bias. Systematic reviews in 2010^188^ and 2017^189^ investigating the effect of chiropractic interventions for insomnia noted minimal overall evidence of effectiveness, with significant methodological limitations. Several insomnia clinical practice guidelines have also attempted to integrate evidence of CAM therapies from previous systematic reviews and meta-analyses, however, significant variability in guideline recommendations exists. For example, a 2021 systematic review documented the frequency and quality of evidence for CAM therapies included in insomnia clinical practice guidelines^190^. Nine insomnia clinical practice guidelines mentioned CAM approaches for insomnia, and 6 made specific CAM recommendations. The quality of evidence used to inform different insomnia clinical practice guidelines, and consequently, the recommendations for/against specific CAM therapies differed considerably between guidelines. For example herbal medication were recommended in some guidelines^191,192^, were specifically not recommended in another^193^, or often not mentioned. In a separate review of evidence-based guidelines, Zhao and colleagues also conducted a 2023 systematic review and critical assessment of complementary and alternative medicine recommendations for the treatment of insomnia in 17 insomnia guidelines^194^. Of the 22 CAM therapies that were identified across guidelines, synthesized evidence and recommendations were “mostly unclear, … uncertain, or conflicting”^194^. While some findings were consistent (e.g., none of the guidelines endorsed the use of valerian, chamomile, kava, hops, melissa, passiflora, tart cherry juice, mindfulness, or music therapy due to insufficient high quality evidence supporting efficacy and safety), there was conflicting recommendations for several CAM therapies. Among the guidelines that did not provide definitive recommendations, most acknowledged that CAM therapies might have potential benefits, however, it was widely acknowledged that the original studies underlying this evidence were methodologically poor and thus it is difficult to reach clear and unambiguous conclusions^194^.

# 6. Insomnia in specific populations

## 6.1. First Nations Peoples

Aboriginal and Torres Strait Islanders are the first people of Australia, making up approximately 3% of the Australian population^195^. In Australian First Nations communities, health is often viewed through the lens of Social Emotional Well Being, a multidimensional model of health encompassing several key domains (e.g., mental health, physical health, connection to land or ‘country’, culture, spirituality, ancestry, family, and community)^196^. Sleep health for First Nations peoples may therefore be considered within this context, especially given First Nations Australians suffer systematic disadvantage in all areas of health and wellbeing due to the ongoing impact of colonisation, racism and dispossession of culture and land^197^.

There is no specific data on the prevalence of chronic insomnia disorder in different samples of First Nations Australians and certainly none that encompass the Social Emotional Well Being focus on sleep health. However, a 2022 systematic review^195^ reported that First Nations Australian adults were more likely than non-Indigenous Australians to report sleep disturbance/symptoms (including difficulties initiating sleep, maintaining sleep, short sleep, and/or disrupted/restless sleep or daytime sleepiness; 35%), including inadequate sleep duration (15-34%), fragmented, irregular, and non-restorative sleep (22%). First Nations Australians experience a higher prevalence of chronic conditions which may further predispose them to sleep difficulties^198^ or exacerbate sleep problems. For example, the prevalence of OSA is between 39-46% which is significantly higher than non-Indigenous Australians^195^. First Nations Australians may experience additional risk factors that predispose them to the development of insomnia^199^, such as overcrowded and unsafe sleep spaces, and remoteness coupled with reduced access to evidence-based and/or culturally adapted treatment approaches^195^. Even sleep hygiene adjustments, which can be helpful in ameliorating symptoms of insomnia (e.g., regulating sleep/wake timing or manipulating bedroom environments) may be harder to manage for First Nations communities. To date, there are no clinical trials specifically focusing on the management of insomnia symptoms, or guidance for adaptations to CBTi programs for First Nations people. Co-designing these programs and adaptations is imperative, but as noted in the above review^195^ there is limited inclusion of First Nations peoples in the design, development, coordination, or implementation of sleep health research studies. One tailored questionnaire to assess daytime sleepiness has been developed especially for remote communities^200^, and other culturally grounded sleep diaries and sleep health assessments have been developed^201^. Significant progress has also been made on developing culturally tailored and co-designed sleep health programs delivered by and for Australian First Nations peoples. The Let’s Yarn About Sleep^201^ (<https://letsyarnaboutsleep.org/>), study is the first sleep health program based on strengths-based, culturally informed approaches to promoting sleep health in First Nations communities addressing initially sleep in adolescents expanding to younger children and adults. However, no culturally tailored questionnaire to assess for insomnia symptoms or severity has been co-designed or validated in First Nations people. The Royal Australian College of General Practitioners “National Guide to preventive healthcare for Aboriginal and Torres Strait Islander people” includes specific information on sleep health recommendations for First Nations people including advice for healthy sleep behaviors, and lists of culturally adapted screening tools^202^. The Australasian Sleep Association and Sleep Health Foundation have also established a joint working party to progress sleep health awareness and resource development for First Nations people. Considerable work remains to be done in this area so the prevalence, assessment and treatment of insomnia in this population is better understood.

## 6.2. Shift work

Shift work is broadly defined as work occurring outside typical 8AM to 6PM hours^203^. Approximately 16% of the working Australian population are employed in shift work^204^. Shift workers may experience an increased risk of sleep difficulties and insomnia symptoms, partly due to a mistimed sleep opportunity relative to the internal circadian rhythm^205^. Shift work may also co-occur with additional stressors that can pre-dispose to the development of insomnia, independently of circadian factors (e.g., workplace stress, injury, trauma, reduced social engagement, increased risks of mental and physical health conditions, etc.). The timing, duration, frequency, and regularity of shift work schedules, in addition to specific tasks, demands, and associated stressors differ considerably between shift work contexts, settings, and occupations.

The prevalence of insomnia appears to be higher in shift workers compared to insomnia prevalence in the general population. A 2020 systematic review and meta-analysis of 5 studies reported that the prevalence of insomnia in shift workers ranges from 13-76%^206^.

Shift workers with an undiagnosed sleep disorder can experience delays in accessing diagnosis and care for sleep disorders^207^. This may be especially common if any sleep difficulties are entirely (mis)attributed to the shift-work schedule or context, rather than the presence of a potential underlying sleep disorder^208^. It is therefore important for healthcare providers to access information on sleep disorder screening tools, routinely screen for sleep disorders in shift workers^209^, and consider differential or comorbid diagnoses.

A 2020 Australian systematic review on sleep hygiene (healthy sleep habits) among shift workers, reported significant variability in types of sleep hygiene practices used by shift workers^205^. More research is warranted to determine the effect of comprehensive recommendations for healthy sleep behaviors for shift work populations^205^. For shift workers with insomnia, more intensive management approaches may be required. For example, sleep hygiene alone is not recommended for the management of insomnia disorder^4^. Two recent meta-analyses of 10 RCTs, and 33 non-randomized controlled trials found no clinically meaningful effects of CBTi on insomnia symptoms in shift workers^210,211^. The blunted response to CBTi in shift workers may be due to the impracticalities or limited feasibility to enact some recommendations of CBTi components while engaged in shift work. For example, some shift work occupations may make it difficult, impractical or impossible to maintain a consistent sleep schedule or adhere to specific bed/wake times over multiple successive weeks during bedtime restriction therapy. Furthermore, increased sleep pressure and exacerbation of any impairment of alertness while working shifts during bedtime restriction therapy^75^ may be an unacceptable side-effect in some shift work settings that require sustained alertness (e.g., emergency settings)^212^. Relapse prevention strategies may also be less effective, if shift work contributes to continued exposure to precipitating factors for insomnia. Adapted insomnia assessment and management approaches specifically tailored for the management of insomnia/sleep disturbance in shift workers that incorporate tailored recommendations for tailored timing of light/dark exposure, psychoeducation about chronobiology, tailored sleep-wake scheduling (with reference to specific shifts and rotations), timing of caffeine/stimulants, and adapted components of CBTi (e.g., identifying barriers and finding alternatives to ‘standard’ sleep restriction therapy protocols that may not be appropriate in shift workers)^212,213^. Shift workers with insomnia may be appropriate candidates for referral to specialist sleep clinicians experienced in managing insomnia in shift workers^214-216^.

## 6.3. Perinatal insomnia

Sleep disturbance, disruption, and insomnia symptoms are frequently reported during the perinatal period^217^. Up to 75% of people report changes in sleep patterns and 20-60% of people report ‘insomnia symptoms’^217^. However, most people reporting sleep disturbance during the perinatal period do not meet criteria for insomnia disorder^20,46^, and consequently, are not suitable for CBTi^217^. Specifically, insomnia diagnostic criteria (See Table 2) indicate that the sleep disturbance should occur *despite adequate opportunity for sleep*. For example, if someone is waking multiple times per night due to infant awakenings and/or feeding, they would not meet insomnia diagnostic criteria. Management approaches may also be different for people with inadequate sleep opportunity. For example, it would not be appropriate to use bedtime restriction therapy to further reduce sleep opportunity and increase sleep pressure/sleepiness in an attempt to consolidate sleep in people reporting sleep difficulties due to limited opportunity for sleep. Similarly, if a client is experiencing awakenings during pregnancy due to leg/back pain, restless legs symptoms, or foetal movements, bedtime restriction therapy would not be indicated (as it would have little impact on these precipitating factors). Instead, tailored sleep education with involvement of bed partner and family may be more appropriate. It is important to distinguish sleep disturbance resulting from insomnia disorder versus inadequate opportunity for sufficient sleep duration and quality during the perinatal period^217^. Most people reporting sleep disturbance during the perinatal period have a reduced opportunity for sleep^217^.

To systematically determine the prevalence of insomnia disorder, versus sleep disruption during the perinatal period, Quin and colleagues^217^ used the question “Do you have this sleep difficulty even when you have the time to sleep, and your sleep environment is comfortable and safe?” and discussed potential threats to sleep opportunity. After taking sleep opportunity into account only 25-50% of those reporting sleep disturbance during the perinatal period met criteria for insomnia disorder^217^.

For patients experiencing insomnia during the perinatal period, clinical trials have found that adapted versions of CBTi are safe and effective^218-221^. Adapted sleep education may include advice about sleep during the perinatal period, understanding the difference between sleep deprivation and insomnia occurring due to different environmental factors, and involvement of family members^220,222^. Stimulus control therapy may be introduced before bedtime restriction therapy, and minimum time in bed recommendations may be increased to account for wakings for caring responsibilities^222^.

## 6.4. Comorbid insomnia and sleep apnea

Insomnia and obstructive sleep apnea (OSA) frequently co-occur^62^. Approximately 30-50% of patients with OSA report insomnia symptoms, and 30-40% of patients with insomnia have comorbid OSA^19,223^. People with comorbid insomnia and sleep apnea (COMISA) generally experience worse sleep, daytime function, mental health, physical health, and quality of life, compared to people with neither disorder, and often compared to people with either insomnia-alone and OSA-alone^18,224^. Three recent population-based cohort studies have reported that COMISA is associated with a 50-70% increased risk of all-cause mortality over 10-20 years of follow-up compared to people with neither disorder, after controlling for socio-demographic characteristics, behavioral factors, and chronic conditions^225-227^.

Patients with COMISA require nuanced assessment and management considerations. Accurate diagnosis of COMISA can be difficult due to the shared symptoms of insomnia and sleep apnea, and difficulty in identifying the underlying causal relationships between insomnia and OSA that may guide the most appropriate treatment sequence and approach^62^. For example, insomnia symptoms may create a barrier to the acceptance and use of continuous positive airway pressure therapy for the management of OSA^228^. Alternatively, a shortage of CBTi providers and perceptions that insomnia symptoms are often a ‘secondary symptom’ of untreated OSA may make it difficult to access CBTi for patients with COMISA^229^. A recent meta-analysis reported that CBTi leads to large improvements in insomnia in patients with comorbid treated and untreated OSA^38^. Two^230,231^ of four^232,233^ recent RCTs have found that CBTi may improve subsequent acceptance and use of positive airway pressure therapy in patients with COMISA, and two recent Australian clinical trials have reported improvement in OSA severity following CBTi^234,235^. An Australian digital CBTi program, Bedtime Window, has been co-designed with individuals with insomnia-alone^80^ and COMISA^236^, and clinical trials have reported large and sustained improvements in insomnia, mental health and daytime function following Bedtime Window in patients with insomnia-alone^80^, insomnia with a high-risk of OSA^237^, and insomnia with confirmed OSA^236^. Ongoing research is required to determine the most appropriate combination and sequence of insomnia and OSA treatments for different patients with COMISA, and to implement tailored management approaches throughout the health system^229^.

## 6.5. Older adults

There are natural changes to sleep as we age. Yet people often believe that sleep patterns and duration should remain constant and unchanging with age. Patterns of sleep-related anxiety and protective behaviors may develop in individuals who become increasingly aware and concerned about age-related changes to sleep including; more frequent or prolonged awakenings, earlier waking time, more light sleep and less deep sleep. If people perceive these age-related changes as abnormal, it may cause concern and anxiety about sleep, and the impacts of sleep on reduced health. When paired with media attention highlighting the ‘catastrophic’ consequences of sleep loss, this sleep-related anxiety and protective behaviors increase a person’s likelihood of developing insomnia. It is important to disseminate impartial and evidence-based information about normal changes in sleep across the lifespan (See Resources for Consumers). A 2022 systematic review and meta-analysis of 13 articles investigating the effectiveness of CBTi in older adults reported improvements in sleep onset latency (9.3 minute reduction), wake after sleep onset (23.4 minute reduction), total sleep time (12.4 minute increase), and sleep efficiency (8.4% increase)^238^. Adaptations to CBTi for older adults may include specific information to help clients understand normal age-related changes, adjusting bedtime restriction therapy protocols to recommend initially reducing time in bed to total sleep time plus 30 minutes, administering sleep compression therapy instead of bedtime restriction, and development of realistic expectations of sleep need/patterns^239^. Incorporation of adjunct therapies for an advanced circadian rhythm (e.g., light therapy) may be beneficial in some older adults with an advanced body clock, however more research is needed^240^.

## 6.6. Perimenopause and Menopause

Perimenopausal *symptoms* impact 80–90% of women and are associated with vasomotor, urogenital, cognitive, and psychiatric symptoms as hypo-oestrogenic symptoms become prevalent and intense^241^. The onset or worsening of sleep disturbances also occurs during menopausal transition. The quality of sleep is affected by hormonal fluctuations whilst hot flushes wake the individual. Other normal aging factors and circadian factors (decreased melatonin) further reduce the continuity of sleep. Treatments include CBTi along with hormone therapy and other medication for insomnia as per individual needs and risk factors^242^. A scoping review of 8 studies^243^ investigating CBTi in menopausal women, and sub-group analyses of a systematic review^244^ of 16 RCTs of behavioral interventions for sleep problems in menopausal women both reported consistent improvements in sleep and insomnia severity following CBTi. Suggested adaptations to CBTi include psychoeducation about normal age-related changes to sleep, education about menopausal transition, and physiology of hot flushes, potential benefits of bedtime restriction therapy on moderating the arousal threshold to hot flushes, a greater focus on environmental and bedroom temperature, and identifying and altering responses to hot flush triggers^245^. Standardisation of treatment for the management of insomnia in menopause is still not available.

## 6.7. Cancer

Sleep disturbances are frequently experienced by people with cancer. Prevalence rates vary between sub-groups of patients, timing of cancer diagnosis/treatment/remission, different types of cancer/treatments, and definitions of insomnia (e.g., insomnia symptoms vs [short-term/chronic] insomnia disorder)^246,247^. The 3P (or 4P) model of insomnia aetiology has been used to understand the development of insomnia in the context of cancer^248^. Numerous factors associated with cancer symptoms, diagnosis and treatment side-effects may precipitate insomnia symptoms. However, as with other comorbid conditions, psycho-behavioral perpetuating factors can develop and maintain the insomnia independently of cancer symptoms/treatment over time. Insomnia symptoms may then interact with cancer related factors or management and further reduce quality of life and mental health.

Meta-analyses have investigated several interventions for insomnia in the context of cancer (and interventions accessed at different stages of cancer diagnosis/management), including CBTi^249-251^, complementary medications^250^, exercise, mindfulness-based therapies^252^, melatonin^253^ and other medications for insomnia^252,254^. Meta-analyses have most consistently found that CBTi is the most effective treatment for insomnia in patients with cancer^250-252,255^. A 2022 network meta-analysis found that group-based CBTi may promote the greatest post-treatment and short-term effects on insomnia severity and sleep parameters of different modalities^249^.

Adaptations to CBTi in the context of cancer may be important^256,257^. For example, applying flexibility to rigid stimulus control therapy/bedtime restriction therapy recommendations in the context of cancer-related pain/fatigue, hospitalisations, and other treatment-related factors may be needed. Close coordination between patients, families/carers and healthcare providers responsible for managing insomnia and cancer is important to prevent conflicting recommendations. For example, patients may be instructed to remain in bed for longer to increase their opportunity for rest during cancer treatment, while traditional Stimulus Control Therapy recommendations include avoiding excessive wake time in bed and long daytime naps. Cognitive therapy may be adapted to include specific cancer-related beliefs/attitudes, and the relationship that these may have with sleep/ruminations at night (for example, strongly held beliefs about the association between sleep duration and mortality, that may perpetuate anxiety about sleep and create a barrier to engagement with behavioral therapies for insomnia). Stepped-care models for insomnia management in the context of comorbid cancer may be one approach to increase access to CBTi in patients with cancer/in remission^258-260^.

# 7. References

1.	Ree M, Junge M, Cunnington D. Australasian Sleep Association position statement regarding the use of psychological/behavioral treatments in the management of insomnia in adults. *Sleep Medicine*. 2017;36:S43-S47. doi:<http://dx.doi.org/10.1016/j.sleep.2017.03.017>

2.	Riemann D, Espie CA, Altena E, et al. The European Insomnia Guideline: An update on the diagnosis and treatment of insomnia 2023. *Journal of sleep research*. 2023;32(6):e14035.

3.	Qaseem A, Kansagara D, Forciea MA, Cooke M, Denberg TD. Management of chronic insomnia disorder in adults: a clinical practice guideline from the American College of Physicians. *Annals of internal medicine*. 2016;165(2):125-133. doi:<https://doi.org/10.7326/M15-2175>

4.	Edinger JD, Arnedt JT, Bertisch SM, et al. Behavioral and psychological treatments for chronic insomnia disorder in adults: an American Academy of Sleep Medicine systematic review, meta-analysis, and GRADE assessment. *Journal of Clinical Sleep Medicine*. 2021;17(2):263-298.

5.	Wilson S, Anderson K, Baldwin D, et al. British Association for Psychopharmacology consensus statement on evidence-based treatment of insomnia, parasomnias and circadian rhythm disorders: An update. *Journal of Psychopharmacology*. 2019;33(8):923-947. doi:<https://doi.org/10.1177/0269881119855343>

6.	Sweetman A, McEvoy RD, Frommer MS, et al. Promoting sustained access to cognitive behavioral therapy for insomnia in Australia: a system-level implementation program. *Journal of Clinical Sleep Medicine*. 2024:jcsm. 11374.

7.	Miller CB, Valenti L, Harrison CM, et al. Time trends in the family physician management of insomnia: the Australian experience (2000–2015). *J Clin Sleep Med*. 2017;13(06):785-790. doi:<https://doi.org/10.5664/jcsm.6616>

8.	David Gonzalez-Chica, Mumtaz Begum, Carla Bernardo, Elizabeth Hoon, Alexander Sweetman, Stocks N. Trends and patterns of benzodiazepines and Z-drugs prescriptions in Australian general practice: A national study (2011–2018). *Drug and Alcohol Review*. 2022;42(2):427-438. doi:<https://doi.org/10.1111/dar.13561>

9.	Cheung JM, Bartlett DJ, Armour CL, Laba T-L, Saini BJBsm. To drug or not to drug: a qualitative study of patients’ decision-making processes for managing insomnia. 2018;16(1):1-26.

10.	Cheung JM, Bartlett DJ, Armour CL, Glozier N, Saini B. Insomnia patients' help-seeking experiences. *Behavioral sleep medicine*. 2014;12(2):106-122.

11.	Haycock J, Hoon E, Sweetman A, Lack L, N L. The management of insomnia by Australian psychologists: a qualitative study. *Aus Psychologist*. 2022;57(5):290-300. doi:<https://doi.org/10.1080/00050067.2022.2089544>

12.	Haycock J, Grivell N, Redman A, et al. Primary care management of chronic insomnia: a qualitative analysis of the attitudes and experiences of Australian general practitioners. *BMC Family Practice*. 2021;22(158):1-11. doi:<https://doi.org/10.1186/s12875-021-01510-z>

13.	Kippist C, Wong K, Bartlett D, Saini B. How do pharmacists respond to complaints of acute insomnia? A simulated patient study. *International journal of clinical pharmacy*. 2011;33:237-245.

14.	Grivell N, Feo R, Vakulin A, et al. An Interpretive Description of the Knowledge, Attitudes and Experiences of Family Practice Nurses Towards Sleep Health Care Within Australia. *American Thoracic Society*. 2020:A4631-A4631.

15.	Meaklim H, Rehm IC, Junge MF, et al. Development of a Novel Behavioral Sleep Medicine Education Workshop Designed to Increase Trainee Psychologists’ Knowledge and Skills in Insomnia Management. *Behavioral Sleep Medicine*. 2023:1-15.

16.	ASA. Over 22,000 primary care staff provided sleep education through ASA grants. March, 2024, <https://www.sleep.org.au/Public/News/F-ASA-news/Feb/ASA-grants.aspx>

17.	Evans MA, Hasler BP. CBT-I for patients with phase disorders or insomnia with circadian misalignment. *Adapting cognitive behavioral therapy for insomnia*. Elsevier; 2022:63-95.

18.	Sweetman A, Lack L, Bastien C. Co-Morbid Insomnia and Sleep Apnea (COMISA): Prevalence, Consequences, Methodological Considerations, and Recent Randomized Controlled Trials. *Brain Sciences*. 2019;9(12):371. doi:<http://doi.org/10.3390/brainsci9120371>

19.	Sweetman A, Lack L, McEvoy RD, et al. Bi-directional relationships between co-morbid insomnia and sleep apnea (COMISA). *Sleep Med Rev*. 2021:101519. doi:<https://doi.org/10.1016/j.smrv.2021.101519>

20.	AASM. *International Classification of Sleep Disorders, 3rd Ed, Text Revision*. 3 ed. 2023.

21.	Staner L. Comorbidity of insomnia and depression. *Sleep Medicine Reviews*. 2010;14(1):35-46.

22.	Ohayon MM, Shapiro CM, Kennedy SH. Differentiating DSM-IV anxiety and depressive disorders in the general population: comorbidity and treatment consequences. *The Canadian Journal of Psychiatry*. 2000;45(2):166-172.

23.	Sweetman A, Van Ryswyk E, Vakulin A, et al. Co-occurring depression and insomnia in Australian primary care: a narrative review of recent scientific evidence. *Medical Journal of Australia*. 2021;215(5):230-236. doi:<https://doi.org/10.5694/mja2.51200>

24.	Hertenstein E, Feige B, Gmeiner T, et al. Insomnia as a predictor of mental disorders: A systematic review and meta-analysis. *Sleep medicine reviews*. 2019;43:96-105.

25.	Baglioni C, Battagliese, G., Feige, B., Spiegelhalder, K., Nissen, C., Voderholzer, U., Lombardo, C. & Riemann, D. Insomnia as a predictor of depression: A meta-analytic evaluation of longitudinal epidemiological studies. *Journal of Affective Disorders*. 2011;135(1-3):10-19. doi:<https://doi.org/10.1016/j.jad.2011.01.011>

26.	Gebara MA, Siripong N, DiNapoli EA, et al. Effect of insomnia treatments on depression: A systematic review and meta‐analysis. *Depression anxiety*. 2018;35(8):717-731.

27.	Hertenstein E, Trinca E, Wunderlin M, et al. Cognitive behavioral therapy for insomnia in patients with mental disorders and comorbid insomnia: A systematic review and meta-analysis. *Sleep medicine reviews*. 2022:101597.

28.	Boland EM, Goldschmied JR, Gehrman PR. Does insomnia treatment prevent depression? *Sleep*. 2023;46(6):zsad104.

29.	Khurshid KA. Comorbid insomnia and psychiatric disorders: an update. *Innovations in clinical neuroscience*. 2018;15(3-4):28.

30.	Harvey AG, Soehner AM, Kaplan KA, et al. Treating insomnia improves mood state, sleep, and functioning in bipolar disorder: a pilot randomized controlled trial. *Journal of consulting and clinical psychology*. 2015;83(3):564.

31.	Kaplan KA, Harvey AG. Behavioral treatment of insomnia in bipolar disorder. *American Journal of Psychiatry*. 2013;170(7):716-720.

32.	Shapiro CM, Devins GM, Hussain M. ABC of sleep disorders. Sleep problems in patients with medical illness. *BMJ: British Medical Journal*. 1993;306(6891):1532.

33.	Taylor DJ, Mallory, L. J., Lichstein, K. L., Durrence, H., Riedel, B. W. & Bush, A. J. Comorbidity of chronic insomnia with medical problems. *Sleep*. 2007;30(2)

34.	Wu JQ, Appleman, E. R., Salazar, R. D., & Ong, J. C. Cognitive behavioral therapy for insomnia comorbid with psychiatric and medical conditions: A meta-analysis. *JAMA*. 2015;

35.	Andersen SG, Sæterhagen H, Pacheco AP, et al. The effects of cognitive behavioral therapy for insomnia in patients with cardiovascular disease: a systematic review and meta-analysis. *Journal of Clinical Sleep Medicine*. 2025:jcsm. 11656.

36.	Selvanathan J, Pham C, Nagappa M, et al. Cognitive Behavioral Therapy for Insomnia in Patients with Chronic Pain-A Systematic Review and Meta-Analysis of Randomized Controlled Trials. *Sleep Medicine Reviews*. 2021:101460.

37.	Salazar-Méndez J, Viscay-Sanhueza N, Pinto-Vera C, et al. Cognitive behavioral therapy for insomnia in people with chronic musculoskeletal pain. A systematic review and dose-response meta-analysis. *Sleep Medicine*. 2024;

38.	Sweetman A, Farrell S, Wallace D, Crawford M. The effect of cognitive behavioural therapy for insomnia in people with co-morbid insomnia and sleep apnoea: A systematic review and meta-analysis. *Journal of Sleep Research*. 2023;32(6)doi:10.1111/jsr.13847

39.	Song ML, Park KM, Motamedi GK, Cho YW. Cognitive behavioral therapy for insomnia in restless legs syndrome patients. *Sleep medicine*. 2020;74:227-234.

40.	Appleton SL, Gill TK, Lang CJ, et al. Prevalence and comorbidity of sleep conditions in Australian adults: 2016 Sleep Health Foundation national survey. *Sleep health*. 2018;4(1):13-19.

41.	McArdle N, Reynolds AC, Hillman D, et al. Prevalence of common sleep disorders in a middle-aged community sample. *Journal of clinical sleep medicine*. 2022;18(6):1503-1514.

42.	Reynolds A, Appleton S, Gill T, Adams R. Chronic insomnia disorder in Australia: A report to the Sleep Health Foundation. *Sleep Health Foundation Special Report*. 2019;doi:<https://www.sleephealthfoundation.org.au/news/special-reports/chronic-insomnia-disorder-in-australia.html>

43.	Perlis ML, Vargas I, Ellis JG, et al. The Natural History of Insomnia: the incidence of acute insomnia and subsequent progression to chronic insomnia or recovery in good sleeper subjects. *Sleep*. 2019;

44.	Morin CM, Jarrin DC, Ivers H, Mérette C, LeBlanc M, Savard J. Incidence, persistence, and remission rates of insomnia over 5 years. *JAMA network open*. 2020;3(11):e2018782-e2018782.

45.	Ellis JG, Perlis ML, Neale LF, Espie CA, Bastien CH. The natural history of insomnia: focus on prevalence and incidence of acute insomnia. *Journal of psychiatric research*. 2012;46(10):1278-1285.

46.	APA. *Diagnostic and statistical manual of mental disorders (5th ed, text rev.)*. 2022.

47.	Royal Australian College of General Practitioners (RACGP). *Prescribing drugs of dependence in general practice, Part B: Benzodiazepines*. 2019;

48.	Glass J, Lanctôt KL, Herrmann N, Sproule BA, Busto UE. Sedative hypnotics in older people with insomnia: meta-analysis of risks and benefits. *BMJ*. 2005;331(7526):1169. doi:<https://doi.org/10.1136/bmj.38623.768588.47>

49.	Barroso D, Hespanhol L, Siegloch ML, et al. Effect of Minimal Cognitive Behavioral Therapy for Patients with Acute Insomnia: A Systematic Review and Meta-Analysis. *Sleep Medicine*. 2024;

50.	Ellis JG, Cushing T, Germain AJS. Treating acute insomnia: a randomized controlled trial of a “single-shot” of cognitive behavioral therapy for insomnia. 2015;38(6):971-978.

51.	Sweetman A, Andronis C, Hancock K, Stocks N, Lack L, McEvoy RD. General practitioner assessment and management of insomnia in adults. *Aust J Gen Pract*. 2023;52(10):691-698.

52.	Buysse DJ, Arnedt JT, Buenaver L, et al. Combination treatment for chronic insomnia disorder in adults: an American Academy of Sleep Medicine systematic review, meta-analysis, and GRADE assessment. *Journal of Clinical Sleep Medicine*. 2026;22(1):58.

53.	Wiklund T, Molander P, Lindner P, Andersson G, Gerdle B, Dragioti E. Internet-delivered cognitive behavioral therapy for insomnia comorbid with chronic pain: randomized controlled trial. *Journal of Medical Internet Research*. 2022;24(4):e29258.

54.	Yang Y-Y, Jun S. The effects of cognitive behavioral therapy for insomnia among college students with irritable bowel syndrome: a randomized controlled trial. *International Journal of Environmental Research and Public Health*. 2022;19(21):14174.

55.	Mehrotra R, Cukor D, McCurry SM, et al. Effectiveness of Existing Insomnia Therapies for Patients Undergoing Hemodialysis: A Randomized Clinical Trial. *Annals of Internal Medicine*. 2024;177(2):177-188.

56.	Nguyen S, McKay A, Wong D, et al. Cognitive behavior therapy to treat sleep disturbance and fatigue after traumatic brain injury: a pilot randomized controlled trial. *Archives of physical medicine and rehabilitation*. 2017;98(8):1508-1517. e2.

57.	Palesh O, Solomon N, Hofmeister E, et al. A novel approach to management of sleep-associated problems in patients with breast cancer (MOSAIC) during chemotherapy: a pilot study. *Sleep*. 2020;43(10):zsaa070.

58.	Paardekooper D, Thayer Z, Miller L, Nikpour A, Gascoigne MB. Group-based cognitive behavioral therapy program for improving poor sleep quality and quality of life in people with epilepsy: A pilot study. *Epilepsy & Behavior*. 2020;104:106884.

59.	Waters F, Chiu VW, Dragovic M, Ree M. Different patterns of treatment response to Cognitive-Behavioural Therapy for Insomnia (CBT-I) in psychosis. *Schizophrenia Research*. 2020;221:57-62.

60.	Talbot LS, Maguen S, Metzler TJ, et al. Cognitive behavioral therapy for insomnia in posttraumatic stress disorder: a randomized controlled trial. *Sleep*. 2014;37(2):327-341.

61.	Committee SoP, Aurora RN, Zak RS, et al. Best practice guide for the treatment of nightmare disorder in adults. *Journal of clinical sleep medicine*. 2010;6(4):389-401.

62.	Sweetman A, Lack L, Catcheside P, et al. Developing a successful treatment for co-morbid insomnia and sleep apnoea. *Sleep Med Rev*. 2017;33:28-38. doi:<http://doi.org/10.1016/j.smrv.2016.04.004>

63.	Allen RP, Walters AS, Montplaisir J, et al. Restless legs syndrome prevalence and impact: REST general population study. *Archives of internal medicine*. 2005;165(11):1286-1292.

64.	Richardson CE, Gradisar M, Barbero SC. Are cognitive “insomnia” processes involved in the development and maintenance of delayed sleep wake phase disorder? *Sleep Medicine Reviews*. 2016;26:1-8.

65.	Roy-Byrne PP, Hommer D. Benzodiazepine withdrawal: overview and implications for the treatment of anxiety. *The American journal of medicine*. 1988;84(6):1041-1052.

66.	Jobert A, Laforgue E-J, Grall-Bronnec M, et al. Benzodiazepine withdrawal in older people: what is the prevalence, what are the signs, and which patients? *European Journal of Clinical Pharmacology*. 2021;77(2):171-177.

67.	Simpson N, Manber R. CBT-I in patients who wish to reduce use of hypnotic medication. *Adapting cognitive behavioral therapy for insomnia*. Elsevier; 2022:437-456.

68.	Jan Y-W, Yang C-M, Huang S-H, Lee H-C. Treatment effect of cognitive-behavior therapy for insomnia combined with usual medication. *Sleep and Biological Rhythms*. 2019;17(3):311-321.

69.	Sweetman A, Putland S, Lack L, et al. The effect of cognitive behavioural therapy for insomnia on sedative-hypnotic use: A narrative review. *Sleep Medicine Reviews*. 2020;56doi:<https://doi.org/10.1016/j.smrv.2020.101404>

70.	Takaesu Y, Utsumi T, Okajima I, et al. Psychosocial Intervention for Discontinuing Benzodiazepine Hypnotics in Patients with Chronic Insomnia: A Systematic Review and Meta-analysis. *Sleep Medicine reviews*. 2019:101214.

71.	Edinger JD, Wamboldt F, Johnson RL, et al. Use of a blinded hypnotic tapering strategy to promote hypnotic discontinuation. *Sleep*. 2025;48(3):zsab270.

72.	Morin CM, Bélanger L, Bastien C, Vallières A. Long-term outcome after discontinuation of benzodiazepines for insomnia: a survival analysis of relapse. *Behaviour research therapy*. 2005;43(1):1-14.

73.	Kyle SD, Morgan, K., Spiegelhalder, K., & Espie, C. A. No pain, no gain: An exploratory within-subjects mixed-methods evaluation of the patient experience of sleep restriction therapy (SRT) for insomnia. *Sleep Medicine*. 2011;12(8):735-747. doi:<https://doi.org/10.1016/j.sleep.2011.03.016>

74.	Kyle SD, Miller, C. B., Rogers, Z., Siriwardena, A. N., MacMahon, K. M. & Espie, C. A. Sleep restriction therapy for insomnia is associated with reduced objective total sleep time, increased daytime somnolence, and objectively-impaired vigilance: Implications for the clinical management of insomnia disorder. *Sleep*. 2014;37(2):229-237.

75.	Sweetman A, McEvoy R, Smith S, et al. The effect of cognitive and behavioral therapy for insomnia on week-to-week changes in sleepiness and sleep parameters in insomnia patients with co-morbid moderate and severe sleep apnea: A randomized controlled trial. *Sleep*. 2020;43(7)doi:<https://doi.org/10.1093/sleep/zsaa002>

76.	Turner A, Ong J, Jones A, Tu A, Salanitro M, Crawford M. Neurocognitive functioning in COMISA patients is better after PAP therapy, but worse after CBT-I: Exploratory analysis of Cognitive Outcomes from the MATRICS Study. *Sleep*. 2022;In pressdoi:<https://doi.org/10.1093/sleep/zsad128>

77.	Winter S. Psychology service model redesign: direct to psychology and stepped care in the multidisciplinary sleep service. *Australian Psychologist*. 2023:1-6.

78.	Management of sleep apnea and insomnia in primary care (MOSIP) trial. <https://www.mosiptrial.org.au/>

79.	Newman P, Oldenhof E, Swierzbiolek B, Andersonwurf J, Szeto J, Staiger P. P082 Lesson's learnt from an AOD team delivering a stepped-care intervention for sleep. *Sleep Advances: A Journal of the Sleep Research Society*. 2022;3(Suppl 1):A56.

80.	Sweetman A, Reynolds C, Richardson C. Digital cognitive behavioural therapy for insomnia versus digital sleep education control in an Australian community‐based sample: a randomised controlled trial. *Internal Medicine Journal*. 2024;54(11):1838-1848.

81.	Forsell E, Jernelöv S, Blom K, et al. Proof of concept for an adaptive treatment strategy to prevent failures in internet-delivered CBT: A single-blind randomized clinical trial with insomnia patients. *American Journal of Psychiatry*. 2019;doi:<https://doi.org/10.1176/appi.ajp.2018.18060699>

82.	Baglioni C, Espie CA, Altena E, et al. Cognitive behavioural therapy for insomnia disorder: Extending the stepped care model. *Journal of Sleep Research*. 2023;32(6):e14016.

83.	Stepanski EJ, & Whyatt, J. K. Use of sleep hygiene in the treatment of insomnia. *Sleep Medicine Reviews*. 2003;7(3):215-225.

84.	Hale L, Troxel W, Buysse DJ. Sleep health: an opportunity for public health to address health equity. *Annual review of public health*. 2020;41:81-99.

85.	McNamara S, Nichols T, Dash S, de Courten M, Calder R. *Sleep: A Core Pillar of Health and Wellbeing. Improving Population Sleep Health to Reduce Preventable Illness and Injury – A Policy Evidence Brief*. 2023.

86.	Ong J, Sholtes D. A mindfulness‐based approach to the treatment of insomnia. *Journal of clinical psychology*. 2010;66(11):1175-1184.

87.	Ong JC, Shapiro SL, Manber R. Mindfulness meditation and cognitive behavioral therapy for insomnia: a naturalistic 12-month follow-up. *Explore*. 2009;5(1):30-36.

88.	Kalmbach DA, Cheng P, Ong JC, et al. Mindfulness-based therapy for insomnia alleviates insomnia, depression, and cognitive arousal in treatment-resistant insomnia: a single-arm telemedicine trial. *Frontiers in Sleep*. 2023;2:1072752.

89.	Ong JC, Shapiro SL, Manber R. Combining mindfulness meditation with cognitive-behavior therapy for insomnia: a treatment-development study. *Behavior therapy*. 2008;39(2):171-182.

90.	Gong H, Ni C-X, Liu Y-Z, et al. Mindfulness meditation for insomnia: A meta-analysis of randomized controlled trials. *Journal of psychosomatic research*. 2016;89:1-6.

91.	Rash JA, Kavanagh VA, Garland SN. A meta-analysis of mindfulness-based therapies for insomnia and sleep disturbance: moving towards processes of change. *Sleep medicine clinics*. 2019;14(2):209-233.

92.	Chen T-L, Chang S-C, Hsieh H-F, Huang C-Y, Chuang J-H, Wang H-H. Effects of mindfulness-based stress reduction on sleep quality and mental health for insomnia patients: a meta-analysis. *Journal of psychosomatic research*. 2020;135:110144.

93.	Garland SN, Carlson LE, Stephens AJ, Antle MC, Samuels C, Campbell TS. Mindfulness-based stress reduction compared with cognitive behavioral therapy for the treatment of insomnia comorbid with cancer: a randomized, partially blinded, noninferiority trial. *Journal of Clinical Oncology*. 2014;32(5):449-457.

94.	Wang X, Li P, Pan C, Dai L, Wu Y, Deng Y. The effect of mind‐body therapies on insomnia: a systematic review and meta‐analysis. *Evidence‐Based Complementary and Alternative Medicine*. 2019;2019(1):9359807.

95.	Kennett L, Bei B, Jackson ML. A randomized controlled trial to examine the feasibility and preliminary efficacy of a digital mindfulness-based therapy for improving insomnia symptoms. *Mindfulness*. 2021;12(10):2460-2472.

96.	Lack LC, Baraniec M. Intensive sleep onset training for sleep onset insomnia. *Sleep*. 2002;25

97.	Harris J, Lack, L., Kemp, K., Wright, H., & Bootzin, R. A randomized controlled trial of intensive sleep retraining (ISR): A brief conditioning treatment for chronic insomnia. *Sleep*. 2012;35(1):49-60.

98.	Harris J, Lack, L., Wright, H., Gradisar, A., & Brooks, A. Intensive sleep retraining treatment for chronic primary insomnia: A preliminary investigation. *Journal of Sleep Research*. 2007;16:276-284.

99.	Bootzin RR. Stimulus control for the treatment of insomnia. *Proceedings of the American Psychological Association*. 1972;7:395-396.

100.	Lack L, Scott H, Micic G, Lovato N. Intensive sleep re-training: from bench to bedside. *Brain sciences*. 2017;7(4):33.

101.	Mair A, Scott H, Lack L. Intensive sleep retraining treatment for insomnia administered by smartphone in the home: an uncontrolled pilot study. *Journal of Clinical Sleep Medicine*. 2022;18(6):1515-1522.

102.	Scott H, Bensen‐Boakes DB, Lovato N, Reynolds A, Perlis M, Lack L. The efficacy of intensive sleep retraining for insomnia: A systematic review and research agenda. *Journal of Sleep Research*. 2023;32(6):e13894.

103.	Scott H, Green M, Jones K, et al. Comparing the efficacy of technology-enabled treatments for insomnia: Study protocol for a randomised controlled trial. *Sleep Advances*. 2025:zpaf010.

104.	Paulos-Guarnieri L, Linares IMP, El Rafihi-Ferreira R. Evidence and characteristics of Acceptance and Commitment Therapy (ACT)-based interventions for insomnia: A systematic review of randomized and non-randomized trials. *Journal of contextual behavioral science*. 2022;23:1-14.

105.	Salari N, Khazaie H, Hosseinian-Far A, et al. The effect of acceptance and commitment therapy on insomnia and sleep quality: A systematic review. *BMC neurology*. 2020;20:1-18.

106.	Hertenstein E, Thiel N, Lüking M, et al. Quality of life improvements after acceptance and commitment therapy in nonresponders to cognitive behavioral therapy for primary insomnia. *Psychotherapy and psychosomatics*. 2014;83(6):371-373.

107.	Lappalainen P, Langrial S, Oinas-Kukkonen H, Muotka J, Lappalainen R. ACT for sleep-internet-delivered self-help ACT for sub-clinical and clinical insomnia: A randomized controlled trial. *Journal of Contextual Behavioral Science*. 2019;12:119-127.

108.	Rafihi-Ferreira E, Hasan R, Toscanini AC, et al. Acceptance and commitment therapy versus cognitive behavioral therapy for insomnia: A randomized controlled trial. *Journal of Consulting and Clinical Psychology*. 2024;92(6):330.

109.	Linares IMP, Jernelöv S, El Rafihi‐Ferreira R. Cognitive and psychological factors associated with treatment response in ACT‐I and CBT‐I for insomnia. *Journal of Sleep Research*.e14473.

110.	Martin JL, Carlson GC, Kelly MR, et al. Novel treatment based on acceptance and commitment therapy versus cognitive behavioral therapy for insomnia: A randomized comparative effectiveness trial in women veterans. *Journal of consulting and clinical psychology*. 2023;91(11):626.

111.	Shin J-W, Kim S, Shin YJ, Park B, Park S. Comparison of acceptance and commitment therapy (ACT) and cognitive behavior therapy (CBT) for chronic insomnia: A pilot randomized controlled trial. *Nature and Science of Sleep*. 2023:523-531.

112.	Winkler A, Rief W. Effect of placebo conditions on polysomnographic parameters in primary insomnia: a meta-analysis. *Sleep*. 2015;38(6):925-931.

113.	Sateia M, Buysse D, Krystal A, Neubauer D, Heald J. Clinical Practice Guideline for the Pharmacologic Treatment of Chronic Insomnia in Adults: An American Academy of Sleep Medicine Clinical Practice Guideline. *Journal of clinical sleep medicine: JCSM: official publication of the American Academy of Sleep Medicine*. 2016;

114.	Australian Medicines Handbook (online). Accessed April 2025, <https://amhonline.amh.net.au/>

115.	Holbrook AM, Crowther R, Lotter A, Cheng C, King DJC. Meta-analysis of benzodiazepine use in the treatment of insomnia. 2000;162(2):225-233.

116.	Buscemi N, Vandermeer B, Friesen C, et al. The efficacy and safety of drug treatments for chronic insomnia in adults: a meta-analysis of RCTs. *Journal of general internal medicine*. 2007;22:1335-1350.

117.	de Mendonça FM, de Mendonça GPRR, Souza LC, et al. Benzodiazepines and sleep architecture: a systematic review. *CNS & Neurological Disorders-Drug Targets-CNS & Neurological Disorders)*. 2023;22(2):172-179.

118.	Brandt J, Leong C. Benzodiazepines and Z-drugs: an updated review of major adverse outcomes reported on in epidemiologic research. *Drugs in R&D*. 2017;17(4):493-507.

119.	Barker MJ, Greenwood KM, Jackson M, Crowe SF. Cognitive effects of long-term benzodiazepine use: a meta-analysis. *CNS drugs*. 2004;18:37-48.

120.	Tannenbaum C, Paquette A, Hilmer S, Holroyd-Leduc J, Carnahan R. A systematic review of amnestic and non-amnestic mild cognitive impairment induced by anticholinergic, antihistamine, GABAergic and opioid drugs. *Drugs & aging*. 2012;29:639-658.

121.	RANZCP. *Guidance for the use of benzodiazepines in psychiatric practice*. 2019. *Profession Practice Guideline 5*.

122.	NPS. Benzodiazepine dependence: reduce the risk. Accessed June 2025, <https://www.nps.org.au/benzodiazepine-dependence>

123.	TG. *Insomnia in Adults: Therapeutic Guidelines*. 2021. *Therapeutic Guidelines*. Accessed October 2024. <https://tgldcdp.tg.org.au/viewTopic?etgAccess=true&guidelinePage=Psychotropic&topicfile=insomnia-adults>

124.	De Crescenzo F, D'Alò GL, Ostinelli EG, et al. Comparative effects of pharmacological interventions for the acute and long-term management of insomnia disorder in adults: a systematic review and network meta-analysis. *The Lancet*. 2022;400(10347):170-184.

125.	Lader M. Benzodiazepines revisited—will we ever learn? *Addiction*. 2011;106(12):2086-2109.

126.	Hallfors DD, Saxe L. The dependence potential of short half-life benzodiazepines: a meta-analysis. *American Journal of Public Health*. 1993;83(9):1300-1304.

127.	Palagini L, Brugnoli R, Dell’Osso BM, et al. Clinical practice guidelines for switching or deprescribing hypnotic medications for chronic insomnia: results of European neuropsychopharmacology and sleep expert’s consensus group. *Sleep Medicine*. 2025;

128.	Horowitz M, Taylor DM. *The Maudsley deprescribing guidelines: antidepressants, benzodiazepines, gabapentinoids and Z-drugs*. John Wiley & Sons; 2024.

129.	Huedo-Medina TB, Kirsch I, Middlemass J, Klonizakis M, Siriwardena AN. Effectiveness of non-benzodiazepine hypnotics in treatment of adult insomnia: meta-analysis of data submitted to the Food and Drug Administration. *Bmj*. 2012;345

130.	Xiang T, Cai Y, Hong Z, Pan J. Efficacy and safety of Zolpidem in the treatment of insomnia disorder for one month: a meta-analysis of a randomized controlled trial. *Sleep medicine*. 2021;87:250-256.

131.	Pan B, Ge L, Lai H, et al. The comparative effectiveness and safety of insomnia drugs: A systematic review and network meta-analysis of 153 randomized trials. *Drugs*. 2023;83(7):587-619.

132.	Dündar Y, Dodd S, Strobl J, Boland A, Dickson R, Walley T. Comparative efficacy of newer hypnotic drugs for the short‐term management of insomnia: a systematic review and meta‐analysis. *Human Psychopharmacology: Clinical and Experimental*. 2004;19(5):305-322.

133.	Hasan F, Lee H-C, Chen P-Y, et al. Comparative efficacy of hypnotics in young and middle-aged adults with insomnia: A systematic review and network meta-analysis. *Sleep and Breathing*. 2023;27(5):2021-2030.

134.	Louzada LL, Machado FV, Quintas JL, et al. The efficacy and safety of zolpidem and zopiclone to treat insomnia in Alzheimer’s disease: a randomized, triple-blind, placebo-controlled trial. *Neuropsychopharmacology*. 2022;47(2):570-579.

135.	Nakajima T, Sasaki T, Nakagome MK, et al. Comparison of the effects of zolpidem and zopiclone on nocturnal sleep and sleep latency in the morning: a cross-over study in healthy young volunteers. *Life Sciences*. 2000;67(1):81-90.

136.	Scharner V, Hasieber L, Sönnichsen A, Mann E. Efficacy and safety of Z-substances in the management of insomnia in older adults: a systematic review for the development of recommendations to reduce potentially inappropriate prescribing. *BMC geriatrics*. 2022;22(1):87.

137.	Treves N, Perlman A, Kolenberg Geron L, Asaly A, Matok I. Z-drugs and risk for falls and fractures in older adults—a systematic review and meta-analysis. *Age and ageing*. 2018;47(2):201-208.

138.	Stranks EK, Crowe SF. The acute cognitive effects of zopiclone, zolpidem, zaleplon, and eszopiclone: a systematic review and meta-analysis. *Journal of clinical and experimental neuropsychology*. 2014;36(7):691-700.

139.	Dumont S, Bloch V, Lillo‐Lelouet A, Le Beller C, Geoffroy PA, Veyrier M. Parasomnias and sleep‐related movement disorders induced by drugs in the adult population: a review about iatrogenic medication effects. *Journal of Sleep Research*. 2025;34(2):e14306.

140.	Mittal N, Mittal R, Gupta M. Zolpidem for insomnia: a double-edged sword. A systematic literature review on Zolpidem-induced complex sleep behaviors. *Indian Journal of Psychological Medicine*. 2021;43(5):373-381.

141.	Curado DF, de Barros VV, Noto AR, Opaleye ES. Dependence on hypnotics: a comparative study between chronic users of benzodiazepines and Z-drugs. *Brazilian Journal of Psychiatry*. 2021;44(3):248-256.

142.	Guerlais M, Grall-Bronnec M, Feuillet F, Gérardin M, Jolliet P, Victorri-Vigneau C. Dependence on prescription benzodiazepines and Z-drugs among young to middle-aged patients in France. *Substance use & misuse*. 2015;50(3):320-327.

143.	Siriwardena AN, Qureshi MZ, Dyas JV, Middleton H, Orner RJBJGP. Magic bullets for insomnia? Patients' use and experiences of newer (Z drugs) versus older (benzodiazepine) hypnotics for sleep problems in primary care. 2008;58(551):417-422.

144.	Yen C-F, Yen C-N, Ko C-H, et al. Correlates of dependence and beliefs about the use of hypnotics among zolpidem and zopiclone users. *Substance use & misuse*. 2015;50(3):350-357.

145.	K S, A R, R A. *Aussie Sleep Snapshot, June 2025*. 2025.

146.	Waldron AY, Spark MJ, Dennis CM. The use of melatonin by children: parents' perspectives. *Journal of Clinical Sleep Medicine*. 2016;12(10):1395-1401.

147.	Erland LA, Saxena PK. Melatonin natural health products and supplements: presence of serotonin and significant variability of melatonin content. *Journal of Clinical Sleep Medicine*. 2017;13(2):275-281.

148.	Cohen PA, Avula B, Wang Y-H, Katragunta K, Khan I. Quantity of melatonin and CBD in melatonin gummies sold in the US. *Jama*. 2023;329(16):1401-1402.

149.	Choi K, Lee YJ, Park S, Je NK, Suh HS. Efficacy of melatonin for chronic insomnia: Systematic reviews and meta-analyses. *Sleep medicine reviews*. 2022;66:101692.

150.	Maruani J, Reynaud E, Chambe J, Palagini L, Bourgin P, Geoffroy PA. Efficacy of melatonin and ramelteon for the acute and long‐term management of insomnia disorder in adults: A systematic review and meta‐analysis. *Journal of Sleep Research*. 2023;32(6):e13939.

151.	TGA. Notice of final decision to amend the current Poisons Standard in relation to melatonin. Accessed August, 2025. <https://www.tga.gov.au/resources/publication/scheduling-decisions-final/notice-final-decision-amend-current-poisons-standard-relation-melatonin>

152.	Auger RR, Burgess HJ, Emens JS, Deriy LV, Thomas SM, Sharkey KM. Clinical practice guideline for the treatment of intrinsic circadian rhythm sleep-wake disorders: advanced sleep-wake phase disorder (ASWPD), delayed sleep-wake phase disorder (DSWPD), non-24-hour sleep-wake rhythm disorder (N24SWD), and irregular sleep-wake rhythm disorder (ISWRD). An update for 2015: an American Academy of Sleep Medicine clinical practice guideline. *Journal of clinical sleep medicine*. 2015;11(10):1199-1236.

153.	Nikles J, Lo V, Giam JA, Saini B. Exploring melatonin prescribing among customers of compounding pharmacies in Australia. *The Medical Journal of Australia*. 2012;196(6):384-385.

154.	Cheung JM, Scott H, Muench A, et al. Comparative short‐term safety and efficacy of hypnotics: A quantitative risk–benefit analysis. *Journal of Sleep Research*. 2024;33(4):e14088.

155.	1.3. Melatonin. Accessed August, 2025. <https://www.tga.gov.au/resources/publication/scheduling-decisions-interim/notice-interim-decisions-proposed-amendments-poisons-standard-acmsaccsjoint-acms-accs-meetings-march-2020/13-melatonin>

156.	TGA. Safety concerns over imported melatonin products. *TGA*. 26/09/2025 2025;

157.	Kuriyama A, Tabata HJSmr. Suvorexant for the treatment of primary insomnia: a systematic review and meta-analysis. 2017;35:1-7.

158.	Kishi T, Matsunaga S, Iwata N. Suvorexant for primary insomnia: a systematic review and meta-analysis of randomized placebo-controlled trials. *PloS one*. 2015;10(8):e0136910.

159.	Khazaie H, Sadeghi M, Khazaie S, Hirshkowitz M, Sharafkhaneh A. Dual orexin receptor antagonists for treatment of insomnia: A systematic review and meta-analysis on randomized, double-blind, placebo-controlled trials of suvorexant and lemborexant. *Frontiers in Psychiatry*. 2022;13:1070522.

160.	Kishi T, Nomura I, Matsuda Y, et al. Lemborexant vs suvorexant for insomnia: a systematic review and network meta-analysis. *Journal of Psychiatric Research*. 2020;128:68-74.

161.	Xue T, Wu X, Chen S, et al. The efficacy and safety of dual orexin receptor antagonists in primary insomnia: A systematic review and network meta-analysis. *Sleep Medicine Reviews*. 2022;61:101573.

162.	Yue J-L, Chang X-W, Zheng J-W, et al. Efficacy and tolerability of pharmacological treatments for insomnia in adults: A systematic review and network meta-analysis. *Sleep medicine reviews*. 2023;68:101746.

163.	Yang M, Morin CM, Schaefer K, Wallenstein GV. Interpreting score differences in the Insomnia Severity Index: using health-related outcomes to define the minimally important difference. *Current medical research and opinion*. 2009;25(10):2487-2494.

164.	Rossi S. Sedating antihistamines. Accessed July 2024, 2024. <https://amhonline.amh.net.au/chapters/allergy-anaphylaxis/antihistamines/sedating-antihistamines?menu=vertical>

165.	Simon FER, Simons KJ. H1 antihistamines: current status and future directions. *World Allergy Organization Journal*. 2008;1(9):145-155.

166.	Griend JPV, Anderson SL. Histamine-1 receptor antagonism for treatment of insomnia. *Journal of the American Pharmacists Association*. 2012;52(6):e210-e219.

167.	ASA. Sleep Health Primary Care Resource: Evidence-based resources and information to assess and manage adult patients with Obstructive Sleep Apnoea and Insomnia. <https://www.sleepprimarycareresources.org.au/>

168.	Sansom L. *Insomnia treatment guideline for pharmacists*. Australian pharmaceutical formulary and handbook.

169.	Everitt H, Baldwin, D. S., Stuart, B., Lipinska, G., Mayers, A., Malizia, A. L., Manson, C. CF. & Wilson, S. Antidepressants for insomnia in adults. *Cochrane Database of Systematic Reviews*. 2018;(5)doi:10.1002/14651858.CD010753.pub2.

170.	Suraev A, Mills L, Abelev SV, Arkell TR, Lintzeris N, McGregor IS. Medical cannabis use patterns for sleep disorders in Australia: results of the cross-sectional CAMS-20 survey. *Nature and Science of Sleep*. 2023:245-255.

171.	MacPhail SL, Bedoya-Pérez MA, Cohen R, Kotsirilos V, McGregor IS, Cairns EA. Medicinal cannabis prescribing in Australia: an analysis of trends over the first five years. *Frontiers in Pharmacology*. 2022;13:885655.

172.	Suraev AS, Marshall NS, Vandrey R, et al. Cannabinoid therapies in the management of sleep disorders: a systematic review of preclinical and clinical studies. *Sleep medicine reviews*. 2020;53:101339.

173.	AminiLari M, Wang L, Neumark S, et al. Medical cannabis and cannabinoids for impaired sleep: a systematic review and meta-analysis of randomized clinical trials. *Sleep*. 2022;45(2):zsab234.

174.	Lavender I, McGregor IS, Suraev A, Grunstein RR, Hoyos CM. Cannabinoids, insomnia, and other sleep disorders. *Chest*. 2022;162(2):452-465.

175.	Walsh JH, Maddison KJ, Rankin T, et al. Treating insomnia symptoms with medicinal cannabis: a randomized, crossover trial of the efficacy of a cannabinoid medicine compared with placebo. *Sleep*. 2021;44(11):zsab149.

176.	Ried K, Tamanna T, Matthews S, Sali A. Medicinal cannabis improves sleep in adults with insomnia: a randomised double‐blind placebo‐controlled crossover study. *Journal of sleep research*. 2023;32(3):e13793.

177.	Narayan AJ, Downey LA, Rose S, Di Natale L, Hayley AC. Cannabidiol for moderate–severe insomnia: a randomized controlled pilot trial of 150 mg of nightly dosing. *Journal of Clinical Sleep Medicine*. 2024;20(5):753-763.

178.	Suraev A, McGregor IS, McCartney D, et al. Acute Effects of Oral Cannabinoids on Sleep and High‐Density EEG in Insomnia: A Pilot Randomised Controlled Trial. *Journal of Sleep Research*. 2026;35(1):e70124.

179.	Wang M, Faust M, Abbott S, et al. Effects of a cannabidiol/terpene formulation on sleep in individuals with insomnia: a double-blind, placebo-controlled, randomized, crossover study. *Journal of Clinical Sleep Medicine*. 2025;21(1):69-80.

180.	Suraev A, McCartney D, Marshall NS, et al. Evaluating possible ‘next day’impairment in insomnia patients administered an oral medicinal cannabis product by night: a pilot randomized controlled trial. *Psychopharmacology*. 2024;241(9):1815-1825.

181.	McCartney D, Suraev A, McGregor IS. The “next day” effects of cannabis use: a systematic review. *Cannabis and cannabinoid research*. 2023;8(1):92-114.

182.	Zhang AL, Xue CC, Lin V, Story DF. Complementary and alternative medicine use by older Australians. *Annals of the New York Academy of Sciences*. 2007;1114(1):204-215.

183.	Malhotra V, Harnett J, McIntyre E, Steel A, Wong K, Saini B. The prevalence and characteristics of complementary medicine use by Australians living with sleep disorders–Results of a cross-sectional study. *Advances in Integrative Medicine*. 2020;7(1):14-22.

184.	Bent S, Padula A, Moore D, Patterson M, Mehling W. Valerian for sleep: a systematic review and meta-analysis. *The American journal of medicine*. 2006;119(12):1005-1012.

185.	Wang F, Lee OE-K, Feng F, et al. The effect of meditative movement on sleep quality: a systematic review. *Sleep medicine reviews*. 2016;30:43-52.

186.	Kim S-A, Lee S-H, Kim J-H, et al. Efficacy of acupuncture for insomnia: a systematic review and meta-analysis. *The American Journal of Chinese Medicine*. 2021;49(05):1135-1150.

187.	Zhao F-Y, Fu Q-Q, Kennedy GA, et al. Can acupuncture improve objective sleep indices in patients with primary insomnia? A systematic review and meta-analysis. *Sleep medicine*. 2021;80:244-259.

188.	Kingston J, Raggio C, Spencer K, Stalaker K, Tuchin PJ. A review of the literature on chiropractic and insomnia. *Journal of chiropractic medicine*. 2010;9(3):121-126.

189.	Goto V, Frange C, Andersen ML, Júnior JM, Tufik S, Hachul H. Chiropractic intervention in the treatment of postmenopausal climacteric symptoms and insomnia: A review. *Maturitas*. 2014;78(1):3-7.

190.	Ng JY, Parakh ND. A systematic review and quality assessment of complementary and alternative medicine recommendations in insomnia clinical practice guidelines. *BMC complementary medicine and therapies*. 2021;21:1-11.

191.	Lam WC, Zhong L, Liu Y, et al. Hong Kong Chinese medicine clinical practice guideline for cancer palliative care: pain, constipation, and insomnia. *Evidence‐Based Complementary and Alternative Medicine*. 2019;2019(1):1038206.

192.	Silvestri R, Aricò I, Bonanni E, et al. Italian Association of Sleep Medicine (AIMS) position statement and guideline on the treatment of menopausal sleep disorders. *Maturitas*. 2019;129:30-39.

193.	Riemann D, Baglioni C, Bassetti C, et al. European guideline for the diagnosis and treatment of insomnia. *Journal of Sleep Research*. 2017;26(6):675-700. doi:<https://doi.org/10.1111/jsr.12594>

194.	Zhao F-Y, Xu P, Kennedy GA, et al. Identifying complementary and alternative medicine recommendations for insomnia treatment and care: a systematic review and critical assessment of comprehensive clinical practice guidelines. *Frontiers in public health*. 2023;11:1157419.

195.	Blunden S, Yiallourou S, Fatima Y. Sleep health and its implications in First Nation Australians: a systematic review. *The Lancet Regional Health–Western Pacific*. 2022;21

196.	Gee G, Dudgeon P, Schultz C, Hart A, Kelly K. Aboriginal and Torres Strait Islander social and emotional wellbeing. *Working together: Aboriginal and Torres Strait Islander mental health and wellbeing principles and practice*. 2014;2:55-68.

197.	Dudgeon P, Gibson C, Bray A. Social and emotional well-being:“Aboriginal health in Aboriginal hands”. *Handbook of rural, remote, and very remote mental health*. 2021:599-621.

198.	AIHW. Health and wellbeing of First Nations people. Accessed June 2025, <https://www.aihw.gov.au/reports/australias-health/indigenous-health-and-wellbeing>

199.	Macniven R, Richards J, Gubhaju L, et al. Physical activity, healthy lifestyle behaviors, neighborhood environment characteristics and social support among Australian Aboriginal and non-Aboriginal adults. *Preventive medicine reports*. 2016;3:203-210.

200.	Benn E, Wirth H, Short T, Howarth T, Heraganahally SS. The Top End Sleepiness Scale (TESS): a new tool to assess subjective daytime sleepiness among indigenous Australian adults. *Nature and Science of Sleep*. 2021:315-328.

201.	Fatima Y, Von Senden R, Bucks RS, et al. A co-designed program for better sleep in Australian First Nations adolescents: protocol for the Let’s Yarn About Sleep adolescent sleep health program. *Sleep Advances*. 2025;6(2):zpaf012.

202.	RACGP. *National Guide to preventive healthcare for Aboriginal and Torres Strait Islander people*. 2024. <https://www.racgp.org.au/clinical-resources/clinical-guidelines/key-racgp-guidelines/national-guide>

203.	Arendt J. Shift work: coping with the biological clock. *Occupational medicine*. 2010;60(1):10-20.

204.	Rajaratnam SM, Howard ME, Grunstein RR. Sleep loss and circadian disruption in shift work: health burden and management. *Medical Journal of Australia*. 2013;199

205.	Shriane AE, Ferguson SA, Jay SM, Vincent GE. Sleep hygiene in shift workers: a systematic literature review. *Sleep Medicine Reviews*. 2020;53:101336.

206.	Brito RS, Dias C, Afonso Filho A, Salles C. Prevalence of insomnia in shift workers: a systematic review. *Sleep Science*. 2021;14(01):47-54.

207.	Reynolds AC, Loffler KA, Grivell N, Brown BW, Adams RJ. Diagnosis and management of sleep disorders in shift workers, with patient informed solutions to improve health services research and practice. *Sleep Medicine*. 2024;113:131-141.

208.	Reynolds AC, Lechat B, Melaku YA, et al. Shift work, clinically significant sleep disorders and mental health in a representative, cross-sectional sample of young working adults. *Scientific reports*. 2022;12(1):16255.

209.	Reynolds AC, Lovato N, Sletten TL, et al. Assessment and management of sleep disorders in shift workers: Challenges and considerations for general practice. *Australian Journal of General Practice*. 2024;53(6):389-393.

210.	Reynolds AC, Sweetman A, Crowther ME, et al. Is cognitive behavioral therapy for insomnia (CBTi) efficacious for treating insomnia symptoms in shift workers? A systematic review and meta-analysis. *Sleep Medicine Reviews*. 2022:101716.

211.	Takano Y, Ibata R, Machida N, Ubara A, Okajima I. Effect of cognitive behavioral therapy for insomnia in workers: A systematic review and meta-analysis of randomized controlled trials. *Sleep medicine reviews*. 2023;71:101839.

212.	Cheng P. CBT-I for patients with shift work disorder. *Adapting Cognitive Behavioral Therapy for Insomnia*. 2022:97-113.

213.	Brückner HA, Ell J, Kalon L, et al. Effectiveness of digital cognitive behavioral therapy for insomnia in nurses with shift work sleep disorder: Results of a randomized controlled trial. *International Journal of Nursing Studies*. 2025:105112.

214.	Murray JM, Magee M, Giliberto ES, et al. Mobile app for personalized sleep–wake management for shift workers: A user testing trial. *Digital Health*. 2023;9:20552076231165972.

215.	Thorne H, Sophocleous RM, Sprajcer M, et al. Examining the Feasibility of an App-based Sleep Intervention for Shiftworkers Using the RE-AIM Framework. *Behavioral Sleep Medicine*. 2025;23(3):369-384.

216.	Shriane AE, Vincent GE, Ferguson SA, Rebar A, Kolbe-Alexander T, Rigney G. Improving sleep health in paramedics through an app-based intervention: a randomised waitlist control pilot trial. *BMC Public Health*. 2024;24(1):2395.

217.	Quin N, Lee JJ, Pinnington DM, Newman L, Manber R, Bei B. Differentiating perinatal Insomnia Disorder and sleep disruption: a longitudinal study from pregnancy to 2 years postpartum. *Sleep*. 2022;45(2)doi:10.1093/sleep/zsab293

218.	Bei B, Pinnington DM, Quin N, et al. Improving perinatal sleep via a scalable cognitive behavioural intervention: findings from a randomised controlled trial from pregnancy to 2 years postpartum. *Psychological medicine*. 2023;53(2):513-523.

219.	Verma S, Quin N, Astbury L, et al. Treating postpartum insomnia: a three arm randomised controlled trial of cognitive behavioural therapy and light dark therapy. *Psychological medicine*. 2023;53(12):5459-5469.

220.	Manber R, Bei B, Simpson N, et al. Cognitive behavioral therapy for prenatal insomnia: a randomized controlled trial. *Obstetrics & Gynecology*. 2019;133(5):911-919.

221.	Bacaro V, Benz F, Pappaccogli A, et al. Interventions for sleep problems during pregnancy: a systematic review. *Sleep medicine reviews*. 2020;50:101234.

222.	Quin N, Tikotzky L, Astbury L, et al. Preventing postpartum insomnia: findings from a three-arm randomized-controlled trial of cognitive behavioral therapy for insomnia, a responsive bassinet, and sleep hygiene. *Sleep*. 2024;47(8):zsae106.

223.	Zhang Y, Ren R, Lei F, et al. Worldwide and regional prevalence rates of co-occurrence of insomnia and insomnia symptoms with obstructive sleep apnea: A systematic review and meta-analysis. *Sleep Medicine Reviews*. 2019;45:1-17. doi:<http://doi.org/10.1016/j.smrv.2019.01.004>

224.	Luyster FS, Buysse, D. J. & Strollo, P. J. Comorbid insomnia and obstructive sleep apnea: Challenges for clinical practice and research. *Journal Of Clinical Sleep Medicine*. 2010;6(2)

225.	Sweetman A, Lechat B, Appleton S, Reynolds A, Adams R, YA M. Association of co-morbid insomnia and sleep apnoea symptoms with all cause mortality: Analysis of the NHANES 2005-2008 data. *Sleep Epidemiology*. 2022;2doi:<https://doi.org/10.1016/j.sleepe.2022.100043>

226.	Bastien Lechat, Kelly A Loffler, Douglas M Wallace, et al. All-cause mortality in people with co-occurring insomnia symptoms and sleep apnea: Analysis of the Wisconsin Sleep Cohort. *Nature and Science of Sleep*. 2022;14:1817-1828. doi:<https://doi.org/10.2147/NSS.S379252>

227.	Lechat B, Appleton S, Melaku Y, et al. Co-morbid insomnia and obstructive sleep apnoea is associated with all-cause mortality. *Euro Resp J*. 2021;60(1)doi:<https://doi.org/10.1183/13993003.01958-2021>

228.	Sweetman A, Lack L, Crawford M, D. W. Co-morbid insomnia and sleep apnea (COMISA): Assessment and management approaches. *Sleep Medicine Clinics*. 2021;17(4):597-617.

229.	Sweetman A, Alessi C, Wallace DM, et al. Models of comorbid insomnia and sleep apnea (COMISA). *Cambridge Handbook of Sleep Models and Theories*. Cambridge University Press; 2024.

230.	Sweetman A, Lack L, Catcheside P, et al. Cognitive and behavioral therapy for insomnia increases the use of continuous positive airway pressure therapy in obstructive sleep apnea participants with co-morbid insomnia: A randomized clinical trial. *Sleep*. 2019;42(12)doi:<https://doi.org/10.1093/sleep/zsz178>

231.	Alessi CA, Fung CH, Dzierzewski JM, et al. Randomized Controlled Trial of an Integrated Approach to Treating Insomnia and Improving Use of Positive Airway Pressure Therapy in Veterans with Comorbid Insomnia Disorder and Obstructive Sleep Apnea. *Sleep*. 2021;44(4)doi:<https://doi.org/10.1093/sleep/zsaa235>

232.	Bjorvatn B, Berge T, Lehmann S, Pallesen S, Saxvig I. No Effect of a Self-help Book for Insomnia in Patients with Obstructive Sleep Apnea and Comorbid Chronic Insomnia–a Randomized Controlled Trial. *Frontiers in Psychology*. 2018;9:2413. doi:<https://doi.org/10.3389/fpsyg.2018.02413>

233.	Ong JC, Crawford MR, Dawson SC, et al. A randomized controlled trial of CBT-I and PAP for obstructive sleep apnea and comorbid insomnia: main outcomes from the MATRICS study. *Sleep*. 2020;43(9)doi:<https://doi.org/10.1093/sleep/zsaa041>

234.	Sweetman A, Lack L, McEvoy D, et al. Cognitive behavioural therapy for insomnia reduces sleep apnoea severity: A randomised controlled trial. *ERJ OR*. 2020;6(2)doi:<http://doi.org/10.1183/23120541.00161-2020>

235.	Brooker EJ, Landry SA, Genta PR, Abdelmessih GT, Edwards BA, Drummond SP. Cognitive behavioral therapy for insomnia is associated with reduced sleep apnea severity but not its endotype traits in those with comorbid insomnia and sleep apnea. *Journal of Clinical Sleep Medicine*. 2025;21(6):1041-1051.

236.	Sweetman A, Richardson C, Smith A, Reynolds C. The Effect of Digital Cognitive Behavioural Therapy for Insomnia in People With Co‐Morbid Insomnia and Sleep Apnoea (COMISA): A Pilot Randomised Controlled Trial. *Journal of Sleep Research*. 2025;35(1)

237.	Sweetman A, Reynolds C, Lack L, et al. Effect of high-risk sleep apnea on treatment-response to a tailored digital cognitive behavioral therapy for insomnia program: a quasi-experimental trial. *Frontiers in Sleep*. 2024;3:1355468.

238.	Huang K, Li S, He R, et al. Efficacy of cognitive behavioral therapy for insomnia (CBT-I) in older adults with insomnia: a systematic review and meta-analysis. *Australasian Psychiatry*. 2022;30(5):592-597.

239.	Hughes JM, Martin JL. CBT-I for older adults. *Adapting cognitive behavioral therapy for insomnia*. Elsevier; 2022:347-366.

240.	Alessi C, Vitiello MV. Insomnia (primary) in older people: non-drug treatments. *BMJ clinical evidence*. 2015;2015:2302.

241.	Troìa L, Garassino M, Volpicelli AI, et al. Sleep Disturbance and Perimenopause: A Narrative Review. *Journal of Clinical Medicine*. 2025;14(5):1479.

242.	Attarian H, Hachul H, Guttuso T, Phillips B. Treatment of chronic insomnia disorder in menopause: evaluation of literature. *Menopause*. 2015;22(6):674-684.

243.	Ntikoudi A, Owens DA, Spyrou A, Evangelou E, Vlachou E. The Effectiveness of Cognitive Behavioral Therapy on Insomnia Severity Among Menopausal Women: A Scoping Review. *Life*. 2024;14(11):1405.

244.	Lam CM, Hernandez-Galan L, Mbuagbaw L, Ewusie JE, Thabane L, Shea AK. Behavioral interventions for improving sleep outcomes in menopausal women: a systematic review and meta-analysis. *Menopause*. 2022;29(10):1210-1221.

245.	Meers JM, Dawson DB, Nowakowski S. CBT-I for perimenopause and postmenopause. *Adapting Cognitive Behavioral Therapy for Insomnia*. Elsevier; 2022:333-346.

246.	Savard J, Villa J, Ivers H, Simard S, Morin CM. Prevalence, natural course, and risk factors of insomnia comorbid with cancer over a 2-month period. *Journal of clinical oncology*. 2009;27(31):5233-5239.

247.	Palesh OG, Roscoe JA, Mustian KM, et al. Prevalence, demographics, and psychological associations of sleep disruption in patients with cancer: University of Rochester Cancer Center–Community Clinical Oncology Program. *Journal of clinical oncology*. 2010;28(2):292-298.

248.	Savard J, Savard M-H. Insomnia and cancer: prevalence, nature, and nonpharmacologic treatment. *Sleep Medicine Clinics*. 2013;8(3):373-387.

249.	Gao Y, Liu M, Yao L, et al. Cognitive behavior therapy for insomnia in cancer patients: a systematic review and network meta‐analysis. *Journal of Evidence‐Based Medicine*. 2022;15(3):216-229.

250.	Sun X, Zhang Y, Lu Z, Shu Z, Zhang K, Chen Y. Comparative efficacy of interventions for insomnia in cancer patients: a systematic review and network meta-analysis. *Sleep and Breathing*. 2025;29(2):133.

251.	Squires LR, Rash JA, Fawcett J, Garland SN. Systematic review and meta-analysis of cognitive-behavioural therapy for insomnia on subjective and actigraphy-measured sleep and comorbid symptoms in cancer survivors. *Sleep Medicine Reviews*. 2022;63:101615.

252.	Nissen ER, Neumann H, Knutzen SM, et al. Interventions for insomnia in cancer patients and survivors—a comprehensive systematic review and meta-analysis. *JNCI Cancer Spectrum*. 2024;8(3):pkae041.

253.	Jafari-Koulaee A, Bagheri-Nesami M. The effect of melatonin on sleep quality and insomnia in patients with cancer: a systematic review study. *Sleep medicine*. 2021;82:96-103.

254.	Saeki Y, Sumi Y, Ozaki Y, et al. Proposal for Managing Cancer‐Related Insomnia: A Systematic Literature Review of Associated Factors and a Narrative Review of Treatment. *Cancer Medicine*. 2024;13(22):e70365.

255.	Johnson JA, Rash JA, Campbell TS, et al. A systematic review and meta-analysis of randomized controlled trials of cognitive behavior therapy for insomnia (CBT-I) in cancer survivors. *Sleep medicine reviews*. 2016;27:20-28.

256.	Zhou ES, Suh S, Youn S, Chung S. Adapting cognitive-behavior therapy for insomnia in cancer patients. *Sleep Medicine Research*. 2017;8(2):51-61.

257.	Garland SN. CBT-I during and after a cancer diagnosis. *Adapting Cognitive Behavioral Therapy for Insomnia*. Elsevier; 2022:235-264.

258.	Zhou ES, Michaud AL, Recklitis CJ. Developing efficient and effective behavioral treatment for insomnia in cancer survivors: results of a stepped care trial. *Cancer*. 2020;126(1):165-173.

259.	Diggens J, Bullen D, Maccora J, et al. Feasibility and efficacy of ‘Can-Sleep’: effects of a stepped-care approach to cognitive-behavioral therapy for insomnia in cancer. *Journal of Cancer Survivorship*. 2023:1-9.

260.	Savard J, Ivers H, Savard M-H, et al. Efficacy of a stepped care approach to deliver cognitive-behavioral therapy for insomnia in cancer patients: a noninferiority randomized controlled trial. *Sleep*. 2021;44(11):zsab166.
